# The N-terminus of *Stag1* is required to repress the 2C program by maintaining rRNA expression and nucleolar integrity

**DOI:** 10.1016/j.stemcr.2023.09.004

**Published:** 2023-10-05

**Authors:** Dubravka Pezic, Samuel Weeks, Wazeer Varsally, Pooran S. Dewari, Steven Pollard, Miguel R. Branco, Suzana Hadjur

**Affiliations:** 1 Department of Cancer Biology, Cancer Institute, University College London, 72 Huntley Street, London, UK; 2 Centre for Regenerative Medicine, Institute for Regeneration and Repair, Cancer Research UK Scotland Centre, Edinburgh, UK; 3 Blizard Institute, Faculty of Medicine and Dentistry, QMUL, London, UK

## Abstract

Our understanding of how STAG proteins contribute to cell identity and disease have largely been studied from the perspective of chromosome topology and protein-coding gene expression. Here, we show that STAG1 is the dominant paralog in mouse embryonic stem cells (mESCs) and is required for pluripotency. mESCs express a wide diversity of naturally occurring *Stag1* isoforms, resulting in complex regulation of both the levels of STAG paralogs and the proportion of their unique terminal ends. Skewing the balance of these isoforms impacts cell identity. We define a novel role for STAG1, in particular its N-terminus, in regulating repeat expression, nucleolar integrity, and repression of the two-cell (2C) state to maintain mESC identity. Our results move beyond protein-coding gene regulation via chromatin loops to new roles for STAG1 in nucleolar structure and function, and offer fresh perspectives on how STAG proteins, known to be cancer targets, contribute to cell identity and disease.

## Highlights

• STAG1 is the dominant paralog in mESCs and is required for pluripotency
• mESCs express diverse natural *Stag1* isoforms which together control stem cell identity
• The STAG1 N terminus regulates nucleolar structure and function
• mESCs with N-terminally truncated *Stag1* transcripts convert into 2C-LCs

## Teaser

In this article, Hadjur and colleagues show that STAG1 is the dominant paralog in mouse embryonic stem cells (mESCs) and is required for pluripotency. mESCs express a wide diversity of naturally occurring *Stag1* isoforms and skewing the proportion of these isoforms impacts cell identity. We define a novel role for the STAG1 N terminus in regulating repeat expression, nucleolar integrity, and repression of the two-cell state.

## Introduction

Cohesin is a ubiquitously expressed, multi-subunit protein complex that has fundamental roles in cell biology including sister chromosome cohesion, chromatin topology, and regulation of cell identity (Cuartero et al., 2018; Horsfield et al., 2007; Kline et al., 2018; Leiserson et al., 2015; Romero-Pérez et al., 2019; Viny et al., 2019). Much of our understanding of how cohesin contributes to cell identity has been studied in the context of its roles in protein-coding gene expression and three-dimensional organization of interphase chromatin structure (Hadjur et al., 2009; Kagey et al., 2010; Mishiro and Tsutsumi, 2009; Misulovin et al., 2007; Parelho et al., 2008; Phillips-Cremins et al., 2013; Rao et al., 2014; Vietri Rudan et al., 2015; Wendt et al., 2008). Indeed, loss of cohesin and its regulators results in a dramatic loss of chromatin topology at the level of topologically associated domains (TAD) and chromatin loops, albeit with modest changes to gene expression (Haarhuis et al., 2017; Rao et al., 2017; Schwarzer et al., 2017; Seitan et al., 2013; Sofueva et al., 2013; Wutz et al., 2017; Zuin et al., 2014). This suggests that cohesin’s roles in development and disease extend beyond gene expression regulation and highlight the need to re-evaluate how cohesin regulators shape the structure and function of the genome.

The association of cohesin with chromosomes is tightly controlled by several regulators, including the stromalin antigen protein (known as STAG or SA), which has been implicated in cell identity regulation and disease development (Cuadrado et al., 2019; Lehalle et al., 2017; Leiserson et al., 2015; Soardi et al., 2017; Viny et al., 2019; Yuan et al., 2019). STAG proteins interact with the RAD21 subunit of cohesin and mediate its association with DNA and CTCF (Hara et al., 2014; Li et al., 2020; Orgil et al., 2015; Xiao et al., 2011). Mammalian cells express multiple STAG paralogs, which have >90% sequence conservation in their central domain yet perform distinct functions (Canudas and Smith, 2009; Kojic et al., 2018; Remeseiro et al., 2012a; Winters et al., 2014). It is likely that the divergent N- and C-terminal regions provide functional specificity. For example, the N-terminus of STAG1 contains a unique AT hook (Bisht et al., 2013) that is required for its preferential participation in telomere cohesion (Canudas and Smith, 2009). Why cells have so many STAG proteins and the specific functions that each STAG protein performs to support a given cell state is poorly understood.

The nucleolus is a multifunctional nuclear compartment that coordinates ribosome biogenesis with cell-cycle control and mRNA processing (Boisvert et al., 2007). It forms through self-organization of its constituent proteins and the rDNA gene clusters into a tripartite, phase-separated condensate (Feric et al., 2016; Yao et al., 2019), which is intimately connected to overall nuclear organization (Padeken and Heun, 2014). In line with its liquid-like properties, the nucleolus is itself plastic, undergoing dramatic changes in response to cell cycle, metabolic, or developmental cues. For example, functional nucleoli play an important role in the control of cell identity during early mouse development (Kresoja-Rakic and Santoro, 2019). Two-cell (2C) stage totipotent embryos from mice exhibit “immature” nucleoli with poorly defined structure and low levels of perinucleolar heterochromatin (Aguirre-Lavin et al., 2012; Fulka et al., 2020). This global chromatin accessibility contributes to the expression of the 2C-specific transcription factor DUX and the subsequent activation of MERVL elements (Ishiuchi et al., 2015; Xie et al., 2022). As the mouse embryo reaches the eight-cell stage, cells harbor fully mature phase-separated nucleoli, defined heterochromatin around the nucleolar periphery (Németh et al., 2010), and robust rRNA expression, all of which are essential for cells to commit to differentiation (Gupta and Santoro, 2020; Kresoja-Rakic and Santoro, 2019). In contrast, mouse embryonic stem cells (mESCs) exhibiting nucleolar stress lead to conversion to 2C-like cell (2C-LC) identity *in vitro* (Grow et al., 2021) and nucleolar proteins that control rRNA transcription and processing are essential for 2C-LC repression (Sun et al., 2021), highlighting the tight relationship between rRNA levels, nucleolar structure, and cell identity.

It is known that cohesin is necessary for nucleolar integrity in yeast. Core cohesin subunits have been shown to bind to the non-transcribed region of the rDNA locus (Laloraya et al., 2000) and the 35S and 5S genes form loops that are dependent on Eco1, the cohesin subunit known to acetylate Smc3 and thus stabilize cohesin rings on chromatin (Harris et al., 2014). Consequently, yeast with Eco1 mutations exhibit disorganized nucleolar structure and defective ribosome biogenesis.

Here, we sought to understand how STAG proteins and their divergent ends influence cell identity. We reveal a novel role for STAG1, and in particular its unique N-terminal end, in regulating nucleolar integrity and 2C repression to maintain mESC cell identity. Our results offer fresh perspectives on how STAG proteins, known to be pan-cancer targets (Leiserson et al., 2015), contribute to cell identity and disease. STAG1 binds to repeats associated with nucleolar structure and function including rDNA and LINE-1 and interacts with the Nucleolin/TRIM28 complex that resides within perinucleolar chromatin to maintain nucleolar integrity. Loss of STAG1 or specifically the N-terminus in mESCs leads to reduced nascent rRNA and LINE-1, nucleolar disruption, increased expression of DUX, and conversion of mESCs to 2C-LCs. In addition to presenting a new role for STAG1 in repeat regulation, nucleolar structure, and translation control, our results also reveal a previously unappreciated transcriptional diversity of *Stag1* in stem cells and highlights the complexity of cohesin regulation in mammalian cells. We show that cells change both the levels of STAG paralogs as well as the proportion of their unique terminal ends to control cell identity and point to the importance of the divergent, unstructured ends of STAG1 proteins in nuclear body structure and cell fate control.

## Results

### A functional change in cohesin regulation in cells of different potential

**Figure 1:**
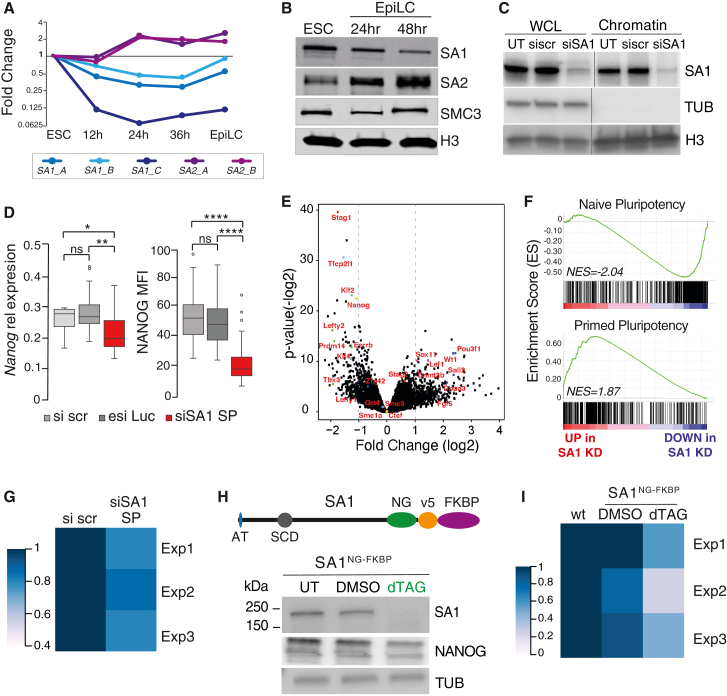
*Stag1* is required for naive pluripotency in mouse ESCs (A) Log2 fold change of *Stag1* (*SA1*) and *Stag* (*SA2*) gene expression assessed by qRT-PCR during *in vitro* mESC differentiation toward EpiLCs. Multiple primer pairs were used for *Stag1* (blue) and *Stag2* (purple) mRNA (see box). Data are derived from two independent replicates. (B) Whole-cell protein extracts (WCL) from naive mESCs and EpiLCs were analyzed by western blot (WB) for levels of SA1, SA2, and SMC3. H3 serves as a loading control. (C) WB analysis of SA1 levels in WCL and chromatin fractions upon treatment with scrambled control siRNAs (si scr) or SmartPool SA1 siRNAs (siSA1) for 24 h in naive mESC cells. Tubulin (TUB) and H3 serve as fractionation and loading controls. (D) Left: relative expression of *Nanog* mRNA by qRT-PCR in naive mESCs upon treatment with si scr, esiLuciferase control, or siSA1. Data are from eight independent replicates. Right, mean fluorescence intensity (MFI) of NANOG protein assessed by immunofluorescence (IF) in naive mESCs treated with same siRNAs as before. Cells were counterstained with DAPI. Data are n > 100 cells/condition across three independent replicates. Whiskers and boxes indicate all and 50% of values, respectively. The central line represents the median. Asterisks indicate a statistically significant difference as assessed using two-tailed t test. ^∗^p < 0.05, ^∗∗^p < 0.005, ^∗∗∗^p < 0.0005, ^∗∗∗∗^p < 0.0001; ns, not significant. (E) Volcano plot displaying the statistical significance (–log2 p value) versus magnitude of change (log2 fold change) from RNA-seq data produced in mESCs treated with si scr or siSA1 for 24 h. Data are from three independent replicates. Vertical blue dashed lines represent changes of 2-fold. Selected genes associated with cohesin, pluripotency, and differentiation have been highlighted in red. (F) Enrichment score (ES) plots from gene set enrichment analysis (GSEA) using curated naive or primed pluripotency gene sets (see experimental procedures). Negative and positive normalized enrichment scores (NES) point to the gene set being over-represented in the top-most down- or upregulated genes in *Stag1* KD mESCs, respectively. Vertical bars refer to individual genes in the gene set and their position reflects the contribution of each gene to the NES. (G) Area occupied by AP^hi^ colonies relative to total colony area in mESCs treated with si scr and si SA1 from 3 independent replicates where n > 50 colonies/condition were counted. (H) CRISPR-Cas9 was used to knock in a NeonGreen-v5-FKBP tag on both alleles of endogenous *Stag1* at the C-terminus (SA1^NG−FKBP^). The resultant STAG1 protein is 42 kDa larger. Shown also are known features of STAG1 including the N-terminal AT-hook (AT) and the stromalin-conserved domain (SCD). WB analysis of STAG1 and NANOG levels in a targeted mESC clone after treatment with DMSO or dTAG. Tubulin (Tub) serves as a loading control. (I) Area occupied by AP^hi^ colonies as above but in wild-type or SA1^NG−FKBP^ mESCs treated with DMSO or dTAG. Data are from three independent replicates where n > 50 colonies/condition were counted. See also Figure S1.

We analyzed the expression levels of cohesin regulators in mESCs by qRT-PCR in different pluripotent populations. During the transition between naive (2i mESCs) and primed epiblast-like pluripotent cells (EpiLCs) *in vitro*, levels of the core cohesin subunits *Smc1* and *Smc3* do not change, while *Stag1* becomes downregulated and *Stag2* becomes upregulated (Figures 1A, 1B, S1A, and S1B). This is supported by western blot (WB) analysis where we observe a 2- to 3-fold higher level of chromatin-associated STAG1 compared with STAG2 in naive (2i) mESCs, while STAG2 levels are 5- to 10-fold higher in EpiLCs (Figures 1B and S1C). These results, together with similar observations (Cuadrado et al., 2019), identify STAG1 as the dominant paralog in naive mESCs and suggest that a switch between *Stag1* and *Stag2* may represent a functionally important change in cohesin regulation at different stages of pluripotency.

### STAG1 is required for pluripotency

To investigate the functional importance of *Stag1* in the regulation of pluripotency, we first established a *Stag1* knockdown (KD) (“siSA1,” experimental procedures) strategy using siRNAs. This resulted in a significant reduction of *Stag1* at the mRNA and protein levels (4- to 5-fold and 8- to 10-fold, respectively), in both serum-grown (FCS) and naive mESCs without affecting the cell cycle (Figures 1C and S1D–S1F). Using Nanog as a marker of naive pluripotency, we observed a significant downregulation of *Nanog* mRNA and protein levels within 24 h of *Stag1* KD in mESCs (Figures 1D and S1G), suggesting that *Stag1* may be required for pluripotency. Global analysis of the mESC transcriptome using RNA sequencing (RNA-seq) upon siRNA-mediated *Stag1* KD revealed that 375 genes were up- and 205 genes were downregulated by at least 2-fold (Figure 1E). Among the downregulated group were genes known to have roles in the maintenance of pluripotency (i.e., *Nanog*, *Tbx3*, *Esrrb*, *Klf4*), while genes associated with exit from pluripotency (*Dnmt3b*, *Fgf5*) and differentiation (i.e., *Pou3f1*, *Sox11*) were upregulated (Figure 1E). Gene set enrichment analysis (GSEA) (Mootha et al., 2003; Subramanian et al., 2005) confirmed a reproducible loss of naive pluripotency-associated gene signature and enrichment for genes associated with primed pluripotency upon *Stag1* KD (Figures 1F and S1H).

The loss of the naive transcriptional program upon *Stag1* KD suggests that mESCs may require *Stag1* for the maintenance of self-renewal. To test this, we plated cells in self-renewal conditions at clonal density and determined the proportion of undifferentiated cells upon *Stag1* KD by measuring the area occupied by the colonies with high alkaline phosphatase activity (AP^hi^). In scrambled siRNA-treated controls, 52% of plated cells retain their naive state, identified by AP^hi^ colonies, which was not significantly different from untreated cells. Upon *Stag1* KD, both the proportion of AP^hi^ colonies and the area they occupy decreased by an average of 20% compared with siRNA controls, indicating that mESCs have a reduced ability to self-renewal in the absence of *Stag1* (Figures 1G and S1I).

We validated these observations by using CRISPR-Cas9 to knock in an mNeonGreen-FKBP12^F36V^ tag (Nabet et al., 2018) at the C-terminus of both alleles of the endogenous *Stag1* locus (SA1^NG_FKBP^) in mESCs (Figures 1H and S1J–S1L). Upon dTAG addition, STAG1 is robustly degraded in a SA1^NG_FKBP^ mESC clone (Figures 1H and S1L). As we had previously observed with siRNA treatment, dTAG-mediated degradation of STAG1 led to a reduction in NANOG protein (reduced by 24% compared with DMSO controls) (Figure 1H), and self-renewal potential was reduced by an average of 38% compared with DMSO-treated cells (Figure 1I). Together, our results are consistent with a requirement for STAG1 in the control of naive pluripotency.

### STAG1 localizes to both euchromatin and heterochromatin

**Figure 2:**
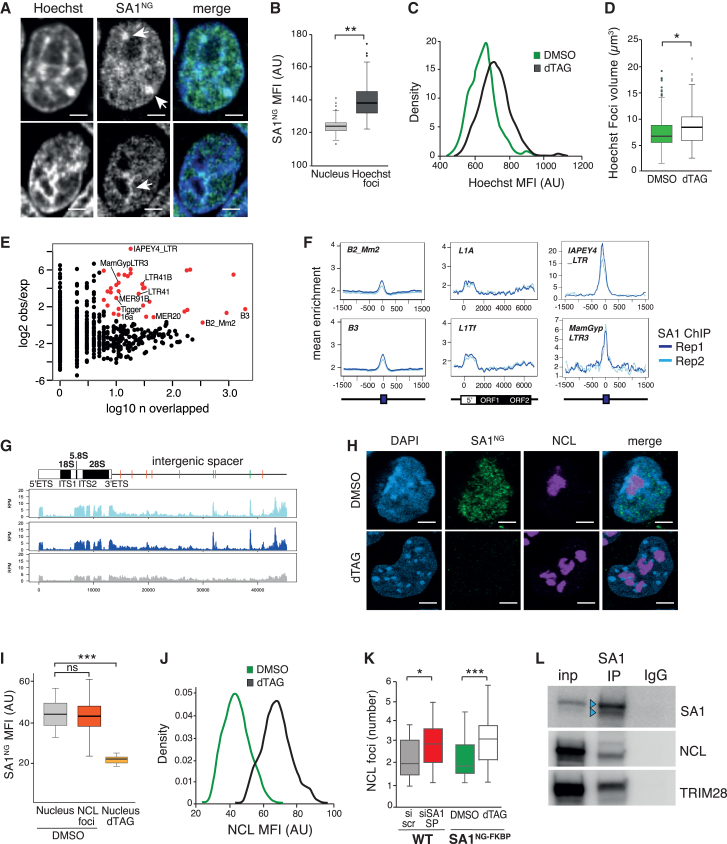
STAG1 is localized to and impacts both euchromatin and heterochromatin compartments (A) Live-cell spinning disk confocal images of two SA1^NG−FKBP^ mESCs counterstained with Hoechst. Arrows indicate notable regions of overlap of STAG1 and Hoechst, including at Hoechst-dense foci and at the nucleolar periphery. NB: puncta within the nucleoplasm can also be observed. Scale bars, 3 μm. (B) Imaris quantification of the MFI of SA1-NeonGreen within the nucleus (light gray) or Hoechst-dense foci (dark gray). Quantifications and statistical analysis were done as above. Data are from n > 100 independent cells/condition in 3 replicates. AU, arbitrary units. (C) Distribution of Hoechst MFI from SA1^NG−FKBP^ mESCs treated with DMSO (green) or dTAG (black). Data are from n > 100 independent cells/condition in 2 replicates. AU, arbitrary units. (D) Imaris quantification of the volume of Hoechst foci in SA1^NG−FKBP^ mESCs treated with DMSO (green) or dTAG (white). Quantifications and statistical analysis were done as above. Data are from n > 100 independent cells/condition in 3 replicates. AU, arbitrary units. (E) Number of copies of each repeat family that overlap an SA1 ChIP-seq peak and the enrichment of binding over random. Shown in red are the repeats that have significant enrichment, with a subset of these labeled. (F) Profiles of the mean enrichment of STAG1 ChIP-seq at select TE repeat families. Shown are full-length elements of the indicated SINE, LINE, and LTR families. Two SA1 ChIP replicates are shown in blue. (G) Top: cartoon of the consensus *Mus musculus* ribosomal DNA (rDNA) (GenBank: BK000964.3), showing the ribosomal genes and the intergenic spacer region which contains several SINE elements (red, B2_Mm2; green, B3). Bottom: Stag1 ChIP replicates and INPUT as in (F) above, aligned to this region. (H) Representative confocal images of MFI of SA1-NeonGreen and Nucleolin (NCL) assessed by IF in SA1^NG−FKBP^ mESCs treated with DMSO or dTAG and counterstained with DAPI. Scale bars, 3 μm. (I) Imaris quantification of the MFI of SA1-NeonGreen from (H) within the nucleus or NCL foci in DMSO and dTAG conditions. Quantifications and statistical analysis were done as above. Data are from n > 100 independent cells/condition in 3 replicates. AU, arbitrary units. (J) Distribution of NCL MFI from SA1^NG−FKBP^ mESCs treated with DMSO (green) or dTAG (black). Data are from n > 100 independent cells/condition in 3 replicates. AU, arbitrary units. (K) Imaris quantification of the number of NCL foci in wild-type mESCs treated with si scr (gray) or siSA1 SP siRNAs (red) and in the SA1^NG−FKBP^ mESC clone treated with DMSO (green) or dTAG (white). Quantifications and statistical analysis were done as above. Data are from n > 100 independent cells/condition in 3 replicates. See also Figure S2. (L) Chromatin immunoprecipitation of STAG1 and IgG from wild-type mESCs and WB for STAG1, NCL, and TRIM28. Blue arrows indicate multiple immunoreactive bands to STAG1. See also Figure S2.

To understand how *Stag1* contributes to pluripotency, we first investigated its subcellular localization. Live-cell imaging of Hoechst-labeled SA1^NG_FKBP^ mESCs revealed the expected and predominant localization of STAG1 in the nucleus with a notable punctate pattern within the nucleoplasm (Figure 2A). STAG1 was also co-localized with Hoechst-dense regions (Figure 2A, arrows) and enriched in Hoechst-dense foci compared with the whole nucleus (Figure 2B). This was of interest since Hoechst stains AT-rich heterochromatin, which is enriched around the nucleolus, at the nuclear periphery and in discreet foci within the nucleoplasm (Padeken and Heun, 2014; Quinodoz et al., 2018). Acute degradation of STAG1 in SA1^NG_FKBP^ mESCs resulted in increased Hoechst signal intensity (Figure 2C) and a significant increase in Hoechst foci volume (Figure 2D). siRNA-mediated *Stag1* KD mESCs revealed similar changes to heterochromatin, as assessed by DAPI and H3K9me3 staining (Figures S2A and S2B).

These observations prompted us to re-analyze STAG1 chromatin immunoprecipitation (IP) followed by chromatin immunoprecipitation sequencing (ChIP-seq) data in mESCs (Cuadrado et al., 2019; Deniz et al., 2020). We calculated the proportion of STAG1 peaks that overlapped genes, repeats (within the Repeat Masker annotation), introns, and intergenic regions not already represented (see experimental procedures). Of the 18,600 STAG1 peaks identified, the majority (76%) are bound to genomic sites that are distinct from protein-coding genes including at repetitive elements and intergenic regions (Figure S2C). Indeed, STAG1 binding was enriched at specific repeat families above random expectation (Figure 2E). These included the DNA transposon and retrotransposon classes, both known to form constitutive heterochromatin in differentiated cell types, and are expressed in early development and involved in regulation of cell fate (Hackett et al., 2017; Percharde et al., 2018). Specifically, STAG1 was enriched at SINE B3 and B2-Mm2 elements (previously shown to be enriched at TAD borders (Dixon et al., 2012); several LTR families, two of which have been previously shown to be associated with CTCF (LTR41, LTR55) (Schwalie et al., 2013) and at evolutionary young and active families of LINE1 elements (L1Tf, L1A) (Figures 2E, 2F, and S2E). We also found that several SINE B3 elements located within the intergenic spacer of the consensus rDNA locus were bound by STAG1 (Figure 2G). The binding of STAG1 at repeats may be dependent on CTCF since many of the bound repeats contained CTCF motifs (Figure S2D).

RNA-seq of siSA1-treated mESCs did not reveal dramatic changes in steady-state transcription of repetitive elements. However, qRT-PCR analysis using primers to ORF1 of STAG1-bound LINE1 and pre-rRNA revealed downregulation of rRNA and a modest but not significant effect on steady-state LINE transcripts (Figure S2F). Together with the microscopy results, the profile of STAG1 peaks suggests that the role of *Stag1* in mESCs may extend beyond protein-coding gene regulation.

### STAG1 supports nucleolar structure in mESCs

Depletion of *Stag1* resulted in a loss of self-renewal and reduced rRNA expression. Furthermore, STAG1 was enriched at repetitive elements including LINE1 and rDNA. As it is known that mESC self-renewal and rRNA synthesis are promoted by a complex containing LINE1 RNA (Percharde et al., 2018), the nucleolar protein Nucleolin (NCL), and the co-repressor TRIM28 (Kap1) (Rowe et al., 2010), we considered whether STAG1 was supporting pluripotency through nucleolar structure and function. We used confocal imaging of SA1^NG_FKBP^ mESCs to assess the co-localization of STAG1 with nucleolar proteins. We observed a similar amount of SA1-NeonGreen (SA1^NG^) within the nucleus compared with the nucleus of mESCs (Figures 2H and 2I). Notably, upon dTAG treatment of SA1^NG_FKBP^ mESCs, there was a significant increase in NCL signal intensity (Figure 2J) as well as increased numbers of nucleolar foci in both dTAG-treated SA1^NG_FKBP^ and in siSA1 KD mESCs (Figures 2K, S2G, and S2H), reminiscent of changes observed during mESC differentiation (Meshorer et al., 2006). Furthermore, STAG1 IP followed by WB in mESCs revealed an interaction with both NCL and Trim28 (Figure 2L), suggesting a direct effect of STAG1 on nucleolar structure and rRNA expression.

### *Stag1* expression is highly regulated in mESCs

**Figure 3:**
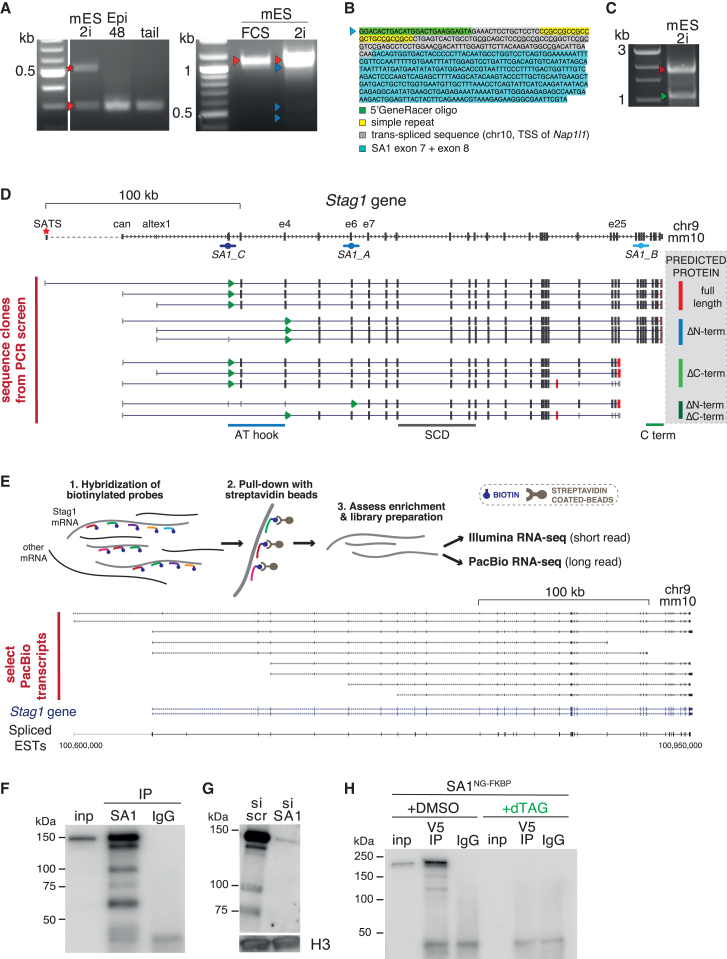
*Stag1* undergoes widespread transcriptional regulation in mESCs (A) 5′ Rapid amplification of cDNA ends (RACE) for *Stag1* in naive mESCs and EpiLCs. Left gel: red star indicates SATS TSSs and red arrow indicates canonical (can) TSSs. Right gel: red arrow indicates full-length *Stag1* with both SATS and can TSSs; dark blue arrow indicates alternatively spliced variants arising from skipping of exons in the 5′ region; light blue arrows indicate the TSSs at exon 6 (e6) and exon 7 (e7). Arrows indicate bands that were cloned and sequenced. (B) The 5′ RACE fragment that identified a new TSS at exon 7 spliced directly to a sequence in *trans* carrying regulatory elements. (C) 3′ RACE for *Stag1* in naive mESCs. Red arrow indicates canonical full-length end; green arrow indicates end in i25. Arrows indicate bands that were cloned and sequenced. (D) Top: schematic of the *Stag1* gene annotation in mm10. The identified TSSs and TTSs from RACE are indicated. Bottom: aligned sequence clones from the PCR mini-screen and their predicted impact on the STAG1 protein (gray box, right). Green arrows and red bars within the transcripts indicate start of the coding sequence and the TTS, respectively. Shown also are the regions that code for the AT hook and the stromalin-conserved domain (SCD). (E) Schematic of the PacBio sequencing methodology (see experimental procedures for full description). Select transcripts sequenced on the PacBio platform, including many isoforms already discovered using RACE and PCR cloning methods above. (F and G) WB analysis of endogenous, chromatin-bound STAG1 protein isoforms from (F) mESCs and (G) upon treatment with si scr and siSA1. H3 serves as a loading control. (H) Chromatin immunoprecipitation for the v5 tag in SA1^NG−FKBP^ mESCs treated with DMSO or dTAG to degrade STAG1. NB: STAG1 bands run 42 kDa higher due to the addition of the tag. See also Figures S3 and S4.

We consistently observed several immunoreactive bands on STAG1 WB (Figure 2L, arrows), which were enriched in mESCs (Figure 1B). To gain a full perspective on how STAG1 may be contributing to nucleolar structure and pluripotency, we first investigated whether *Stag1* may be regulated at the level of transcription in mESCs. Several lines of evidence suggested that this may be the case. First, *Stag1* levels are higher in 2i-grown compared with FCS-grown mESCs (Figures S1B and S1C) and, second, primers positioned along the length of STAG1 amplify mRNAs that respond differently to differentiation (Figure 1A). Thus, we employed a series of approaches to comprehensively characterize *Stag1* mRNAs. First, we used RACE (rapid amplification of cDNA ends) to characterize the starts and ends of *Stag1* mRNAs directly from mESCs. 5′ RACE uncovered four novel alternative transcription start sites (TSSs) in mESCs; ∼50 kb upstream of the canonical *Stag1* TSSs (referred to as “SATS,” and previously identified in Feng et al., 2016) (Figures 3A, 3D, and S3A); between canonical exon 1 and exon 2 (referred to as alternative exon 1 or altex1) (Figures 3A, 3D, and S3D); and at exons 6 and 7 (Figures 3A, 3D, and S3A). Interestingly, the TSS located at exon 7 (e7) was preceded by a sequence located in *trans* to the *Stag1* gene, carrying simple repeats and transcription factor binding sites (Figure 3B). While the frequency of this alternative TSS was significantly lower than the other TSSs, it was identified in multiple RACE replicates, indicating that it may be present in a subset of the mESC population. We also discovered widespread alternative splicing in the 5′ region of *Stag1*, with particularly frequent skipping of exons 2 and 3 (e2/3Δ) and exon 5 (e5Δ) (Figures 3D, S3A, and S3F). Using 3′ RACE, we detected an early termination site in intron 25 and inclusion of an alternative exon 22 introducing an early STOP codon, as well as several 3′ UTRs (Figures 3C, 3D, and S3C).

Next, PCR- and Sanger sequencing-based clonal screening confirmed that the newly discovered 5′ and 3′ ends represent true *Stag1* transcript ends, validated the existence of the e2/3Δ and e5Δ isoforms, confirmed their enrichment in naive mESCs compared with differentiated mouse embryonic fibroblasts (MEFs), and uncovered an isoform lacking exon 31, which encodes a basic domain embedded in the otherwise acidic C-terminal region of *Stag1* (e31Δ) (Figure 3D). To determine the complete sequences of the *Stag1* transcript isoforms and to use a non-PCR-based approach, we performed long-read PacBio Iso-seq from 2i mESC RNA (Figure 3E). This confirmed the diversity of the *Stag1* 5′ and 3′ UTRs, the e31Δ isoform, multiple TSSs including SATS, and early termination events, including in i22 and i25 (Figures 3E and S3E). Importantly, these transcripts all had poly(A) tails, in support of their protein-coding potential. Finally, we validated and quantified the newly discovered splicing events by calculating the frequency (percentage spliced in) of exon splicing in our RNA-seq as well as in published data using the VAST-tools method (Tapial et al., 2017). This confirmed the presence of *Stag1* splicing events in other mESC datasets and supported that several of these were specifically enriched in mESCs (Figure S3F; Table S1).

Interestingly, visual inspection of the genome topology around the *Stag1* locus in our 2i mESC and neural stem cell (NSC) Hi-C data (Barrington et al., 2019) revealed that the *Stag1* gene undergoes significant 3D reorganization as cells differentiate (Figure S4). For example, the *Stag1* TAD switches from the active to the repressive compartment during differentiation, in line with the decrease in *Stag1* levels during differentiation. Furthermore, UMI-4C revealed changes to sub-TAD architecture corresponding to the newly discovered mESC-enriched *Stag1* TSSs and TTSs described above, suggesting that 3D chromatin topology may play a role in facilitating the transcriptional diversity of *Stag1* (Figure S4). Together, our results point to a previously unappreciated diversity of endogenous *Stag1* transcripts in mESCs, prompting us to investigate the importance of these for pluripotency and the nucleolus.

### Multiple STAG1 protein isoforms are expressed in mESCs

*Stag1* transcript diversity was intriguing because many of the events were either specific to mESCs or enriched compared with MEFs and NSCs (Figures S3D and S3F). Furthermore, the transcript variants were predicted to produce STAG1 protein isoforms with distinct structural features and molecular weights (Figures 3D and S3G). For example, the truncation of the N-terminus (e2/3Δ, e5Δ, e6 TSS, and e7 TSS), and thus loss of the AT hook (amino acids 3–58) could impact STAG1 association with nucleic acids. Meanwhile, C-terminal truncated STAG1 isoforms (altex22, i25 end, e31Δ) could affect STAG1-cohesin interactions. It is noteworthy that the evolutionarily conserved stromalin domain (amino acids 296–381) (Orgil et al., 2015), shown to play a role in CTCF interaction (Li et al., 2020), would be retained in the isoforms identified here.

IP of endogenous STAG1 followed by WB revealed multiple bands corresponding to the predicted molecular weights for several protein isoforms and identified by mass spectrometry to contain STAG1 peptides (Figure 3F; Table S2). Similarly, multiple bands of expected sizes were reduced between naive and primed cells (Figure S3H) and sensitive to *Stag1* KD, alongside the canonical, full-length isoform (Figure 3G). Treatment of SA1^NG_FKBP^ mESCs with dTAG followed by WB of chromatin-associated proteins with an antibody to the v5 tag further confirmed the sensitivity of the isoforms to dTAG-mediated degradation (Figure 3H). Thus, complex transcriptional regulation in mESCs gives rise to multiple *Stag1* transcripts and protein isoforms with distinct regulatory regions and coding potential. Our discovery of such naturally occurring isoforms offers a unique opportunity to define the functions of the divergent N- and C-terminal ends of STAG1 in the context of the pluripotent state.

### Experimentally modulating the levels of the N- and C-terminal ends of *Stag1*

**Figure 4:**
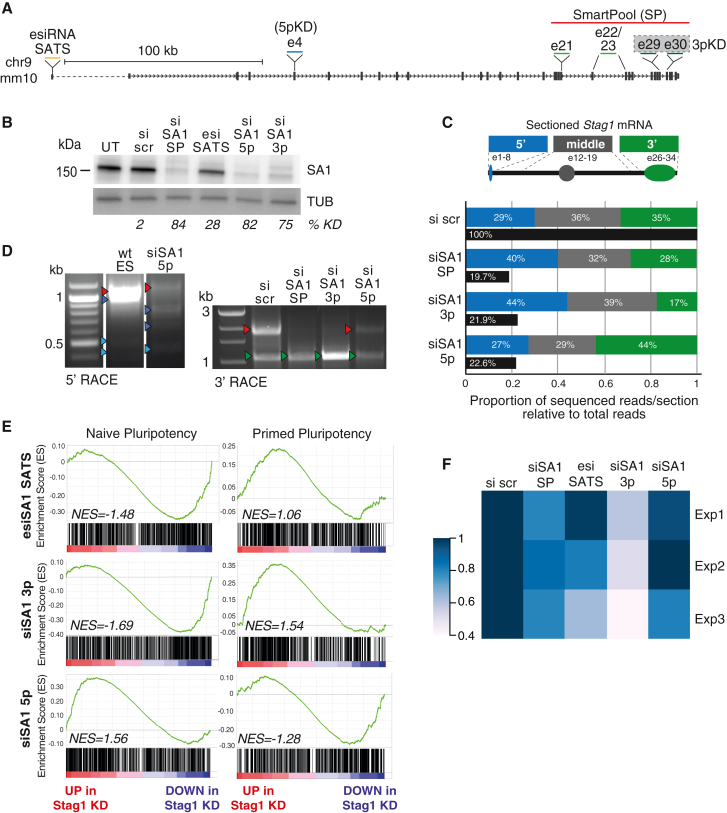
Fluctuations in the levels of the *Stag1* isoforms skews cell fates (A) Schematic of the siRNA pools used in this study. esiRNA SATS represents “enzymatically prepared” siRNAs (see experimental procedures). (B) Representative WB analysis of STAG1 levels in mESC WCL after no treatment (UT), or upon si scr, si SA1 SP, si SA1 3p, si SA1 5p, or esi SATS treatment. Tubulin (TUB) serves as a loading control. The percentage of knockdown (KD) of STAG1 signal normalized to tubulin from this specific experiment is shown. (C) The proportion of RNA-seq reads that align to three sections of *Stag1* mRNA relative to the total number of reads. Shown are the exons within each section that the reads correspond to. RNA-seq from mESCs treated with scr, SP, 5p, and 3p siRNAs was used. Solid black bars represent *Stag1* transcripts per million in the different KDs to highlight the similar degree of KD in all conditions. NB: the change in read proportions from residual *Stag1* in the different KD treatments. (D) Left gel 5′ and right gel 3′ RACE for *Stag1* in mESCs treated with the indicated siRNAs. Arrows indicate bands that were cloned and sequenced and color coded as before. (E) Enrichment score (ES) plots from GSEA using the naive and primed gene sets as in Figure 1E and RNA-seq data from the indicated siRNA-treated mESC samples. (F) Area occupied by AP^hi^ colonies relative to total colony area in mESCs treated with the siRNA panel from three independent replicates. n > 50 colonies/condition were counted.

To study the functional consequences of the *Stag1* isoforms on pluripotency and nucleolar structure, we took advantage of our detailed understanding of *Stag1* transcript diversity to design custom siRNAs to selectively target or retain specific isoforms (Figure 4A). Alongside the siRNAs used in Figure 1 (SmartPool [SP]), we designed siRNAs to specifically target the SATS 5′ UTR (esiSATS), the 5′ end (siSA1-5p), or the 3′ end (siSA1-3p) of Stag1 mRNA (see experimental procedures). We anticipated that the KD panels would reduce Stag1 levels and change the relative proportions of the N- and C-terminal ends of STAG1 in cells. 3p siRNAs were predicted to downregulate full-length and N-terminal truncated isoforms and retain C-terminal truncated isoforms, while 5p siRNAs would specifically retain N-terminal truncated isoforms.

We confirmed that *Stag1* isoform proportions were altered upon siRNA treatment using qRT-PCR, RNA-seq, RACE, and IP. siRNAs to the 5p and 3p ends of *Stag1* reduce full-length *Stag1* mRNA and protein with similar efficiency to SP KDs. esiSATS reduces *Stag1* by ∼30%–50%, indicating that the SATS TSS functions to enhance expression of *Stag1* in naive mESCs (Figures 4B, S5A, and S5B). RNA-seq reads aligning to *Stag1* in the different siRNA treatments were quantified to represent the residual N-terminal (exons 1–8), middle (exons 12–19), and C-terminal (exons 26–34) read proportions. Residual reads in the SP and 3p KDs aligned predominantly to the N-terminal and were relatively depleted from the C-terminal. While the 5p KD had the opposite effect with the least read retention in the N-terminal and relatively more reads aligning to the C-terminal (Figures 4C and S5C). In parallel, we performed RACE to validate changes to the proportions of *Stag1* isoforms. 5′ RACE performed in mESCs treated with 5p siRNA revealed downregulation of full-length *Stag1* transcript while several N-terminal truncated isoforms were upregulated compared with untreated cells (Figure 4D, left panel, blue arrows). Similarly, 3′ RACE captures the canonical 3′ end of *Stag1* (Figure 4D, right panel, red arrows), which is strongly reduced in the SP and 3p siRNA KD samples and to a lesser extent in the 5p KD, further support that the residual transcripts in the 5p KD have C-terminal ends. Meanwhile, the transcript terminating in i25 is substantially enriched upon 3p KD (Figure 4D, green arrows) compared with all other conditions. Thus, the siRNA panel developed here provide us with a powerful tool to modulate the proportion of the naturally occurring *Stag1* isoforms in mESCs and study their potential roles in pluripotency.

### A specific role for the STAG1 C-terminus in the maintenance of naive pluripotency transcriptome

We first quantified the effect of the *Stag1* siRNA KDs on pluripotency gene expression. qRT-PCR for *Nanog* expression and WB for NANOG protein levels revealed that the 3p KD had a similar effect on *Nanog* to SP, with significant downregulation, while, surprisingly, the 5p KD did not reduce *Nanog* (Figure S5D). We prepared independent replicate RNA-seq libraries from the *Stag1* 3p, 5p, and SATS siRNA KDs. We used GSEA as before to probe for signatures of naive or primed pluripotency. In support of our previous results, reducing *Stag1* levels by targeting the mESC-specific SATS promoter leads to downregulation of the naive pluripotency gene signature and upregulation of the primed signature (Figures 4E and S5E), reminiscent of the phenotype from SP KD (Figures 1E and 1F). We again observed a differential effect of the 3p and 5p KDs on naive and primed pluripotency signatures. A similar but more prominent loss of the naive signature was observed in 3p KD RNA-seq compared with SATS and SP while, surprisingly, in 5p KD cells the naive signature was unaffected compared with si scr controls (Figure 4E).

The distinct gene expression profiles of the 3p and 5p KDs were reflected in differences in self-renewal. Cells treated with 3p siRNAs exhibited a significant loss of self-renewal potential, consistent with the loss of the naive pluripotency signature, with only 20% of colonies exhibiting AP staining compared with 30% of colonies in the SP KDs (Figure S1I), and an average reduction of the area occupied by AP+ colonies of 50% compared with si scr controls (Figure 4F). This was not evident in the 5p KD, where the effect on self-renewal was more similar to si scr controls (Figure 4F). Interestingly, unlike siRNA to Stag1, esiSATS results in a variable effect on self-renewal (ranging from between 5% and 35% reduction in AP+ area) (Figure 4F), likely because the SATS TSS is expressed in the most naive cells of the population, the frequency of which varies significantly between FCS populations. Our results further confirm the importance of Stag1 in self-renewal and point to a specific role for the C-terminal of Stag1 in maintaining a naive pluripotency gene expression program.

### The N-terminus of STAG1 supports nucleolar structure and function

**Figure 5:**
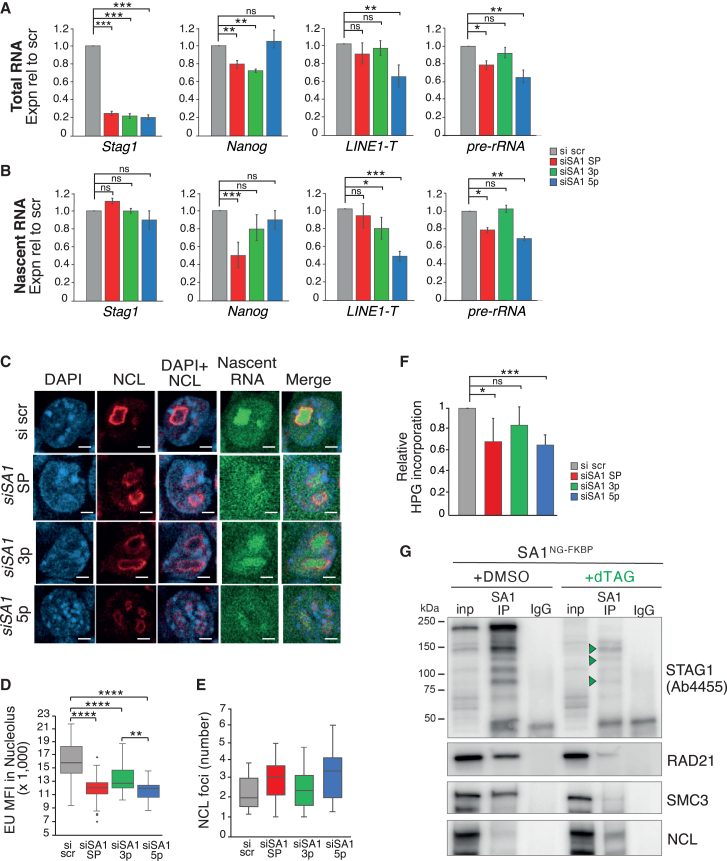
The N- and C-terminal ends of STAG1 regulate expression in different genomic compartments (A and B) Relative expression of *Stag1*, *Nanog*, *LINE1-T*, and *pre-rRNA* by qRT-PCR in mESCs after treatment with the siRNA panel. Shown are (A) total and (B) nascent RNA levels. Data are represented as mean ± SEM and statistical analysis as before. Data are from three independent experiments. (C) Representative confocal images of IF to NCL and nascent RNA in siRNA-treated mESCs labeled with EU-488. Nuclei were counterstained with DAPI. Scale bars, 2 μm. (D) Imaris quantification of the MFI of nascent RNA (EU) within the nucleoli from (C), as defined by a mask made to the NCL IF signal. Quantifications and statistical analysis were done as above. Data are from four independent replicates. n > 50/condition, except for siSA1 5p where n > 35. (E) Imaris quantification of the number of NCL foci in siRNA-treated mESCs. Quantifications and statistical analysis were done as above. Data are from n > 100 independent cells/condition in 2 independent replicates. See also Figure 2K. (F) Analysis of global levels of nascent translation by measuring HPG incorporation using flow cytometry and analyzed using FloJo software. Shown is the quantification of the change in EU incorporation relative to si scr-treated cells. Data are from four independent replicates. (G) Chromatin immunoprecipitation using an N-terminal Stag1 antibody (Ab4455) in SA1^NG−FKBP^ mESCs treated with DMSO or dTAG. Green arrow indicates residual C-terminal truncated STAG1 isoforms. Shown also are WB for the core cohesin subunits RAD21 and SMC3 and NCL. See also Figure S5.

The different effect on naive pluripotency between the 3p and 5p KDs was surprising. We therefore sought to re-examine the effect of our siRNA panel on the STAG1 bound repeats LINE1 and rDNA (Figures 2F and 2G). As we had not observed a significant difference on steady-state levels of repeats from our RNA-seq experiments, we instead purified nascent RNA from mESCs treated with siRNAs. Both the KD and the nascent RNA pull-downs were successful as revealed by qRT-PCR to *Stag1* (Figures 5A and 5B). Consistent with our previous results, total *Nanog* RNA levels were significantly reduced in siSA1 SP and 3p KD but not in 5p KD. Interestingly, this trend was not observed in nascent levels of Nanog RNA where the 3p KD does not have a significant effect, suggesting that the C-terminus may be required for the stability of *Nanog* mRNA instead of its transcription per se (Figures 5A and 5B). Upon *Stag1* SP KD, both steady-state and nascent levels of LINE1 RNA were modestly decreased (Figures 5A, 5B, and S2F). While the 3p KD had a 20% reduction in LINE1 RNA expression, this was not maintained at steady-state levels. However, both nascent and total levels of LINE1 RNA were significantly reduced by 40%–50% of controls in 5p KD mESCs. These results were also observed for pre-rRNA, with only the SP and 5p KD having significant effects on expression. Thus, the N-terminus of *Stag1* plays a distinct role in LINE1 and rDNA expression.

Given the effects on LINE1 and rRNA, we also assessed nucleolar structure and function using our siRNA panel. mESCs were pulsed with 5-ethynyl uridine (EU), which becomes incorporated into nascent RNA and enables detection of newly synthesized RNA. Samples for immunofluorescence (IF) were co-stained with an antibody to NCL to simultaneously quantify nucleoli number and changes in nascent RNA transcription. Cells treated with scrambled siRNA showed a distinct nucleolar structure and the EU signal could be seen throughout the nucleus, with a strong enrichment within the nucleolus as expected from rRNA expression (Figure 5C). While a significant reduction in nascent RNA signal was observed in all KD conditions compared with scrambled controls (Figure S5F), by IF we observed a distinct effect on nascent RNA levels within the nucleolus in the 5p KD. The medians between the three siSA1 KDs were not dramatically different; however, the effect of the 5p KD on nucleolar RNA signal distribution was significantly different from the 3p KD (Figure 5D). This result was consistent with the qRT-PCR analysis of nascent pre-rRNA levels (Figure 5B) and with the significant effect on NCL foci number in 5p KD mESCs (Figure 5E). Consequently, we also observed changes to global translation by assessing the incorporation of L-homopropargylglycine (HPG), an amino acid analog of methionine into mESC using FACS analysis. HPG incorporation was significantly reduced in SP and 5p siRNA-treated mESCs compared with scrambled control (32% and 35% of si Scr) (Figures 5F and S5G). We did observe a modest effect on global nascent translation in 3p KD-treated cells (16% of si scr), although this was not significantly different from scrambled control. Our results reveal distinct roles for the N- and C termini of Stag1 in nucleolar structure and function and pluripotency gene expression, respectively.

The effects observed on rRNA levels and nucleolar function were not associated with changes to expression of ribosome subunit expression (Figure S5H). Thus, we considered whether the regulation of LINE1 expression by the N-terminus of STAG1 influenced nucleolar structure via the NCL/Trim28 complex (Figure 2L). To investigate this, we took advantage of our SA1^NG_FKBP^ mESCs. dTAG treatment can only degrade isoforms containing the FKBP tag inserted into the canonical C-terminal end. Thus SA1^NG_FKBP^ mESCs treated with dTAG should enrich for SA1^ΔC^ isoforms that contain an N-terminus. Indeed, IP of STAG1 using an antibody that recognizes an N-terminal epitope reveals the presence of several N-terminal-enriched SA1ΔC isoforms (Figure 5G, green arrows). WB of this IP material revealed a reduction in the ability of SA1ΔC to interact with the cohesin subunits RAD21 and SMC3, despite similar levels in the input of dTAG-treated cells. Meanwhile, the interaction with NCL was increased in the same lysate (Figure 5G). Taken together, our results are supportive of the different ends of STAG1 interacting with different protein partners to coordinately regulate pluripotency.

### The N-terminus of STAG1 suppresses the 2C-LC state

In addition to promoting rRNA synthesis and self-renewal in mESCs, the LINE-1/NCL/Trim28 complex represses transcriptional program-specific 2C-LCs (Percharde et al., 2018). The phenotypes of the 5p KD, namely reduced rRNA and LINE-1 expression, reduced translation and aberrant nucleolar function, pointed toward possible conversion of cells into a 2C-LC state. We therefore tested whether STAG1, and specifically the N-terminal end, play a role herein.

**Figure 6:**
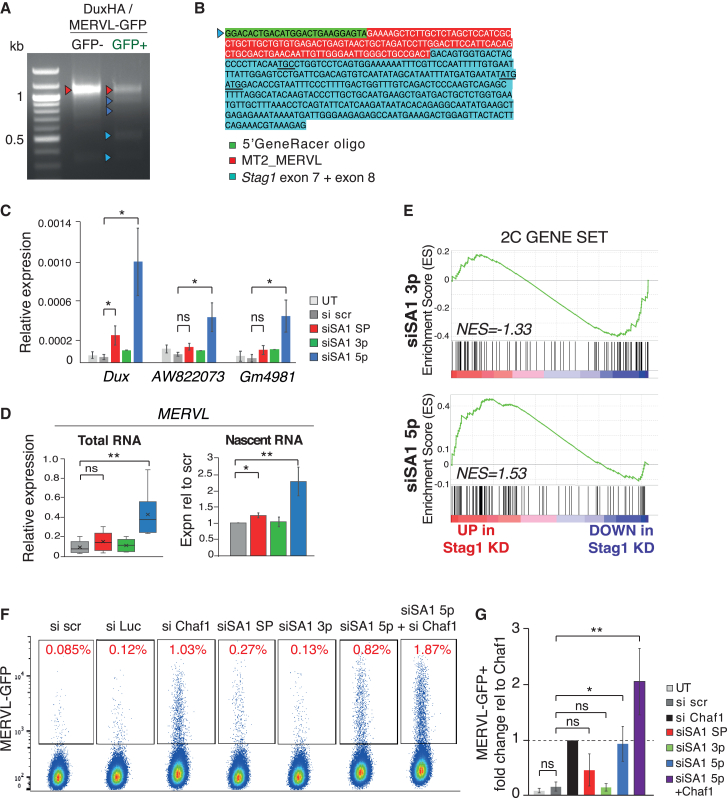
STAG1 N-terminus protects against conversion of ESCs to 2C-LCs (A) 5′ RACE for *Stag1* in Dux-HA MERVL-GFP mESCs with and without sorting for GFP+ cells. Arrows indicate bands that were cloned and sequenced and color coded as described previously. (B) Sequence of the 5′ RACE product identifying a novel *Stag1* TSS from (A) with direct splicing of exon7 to an MT2_MERVL element. (C) Relative expression of several 2C-LC markers in total RNA by qRT-PCR in mESCs after treatment with the siRNA panel. Data are represented as mean ± SEM and statistical analysis as before. Data are from six independent experiments. (D) Relative expression of MERVL repeat element by qRT-PCR in mESCs after treatment with the siRNA panel. Shown are total (left) and nascent RNA (right) levels. Quantifications and statistical analysis as before. Data are from five independent replicates. NB: nascent RNA levels are shown relative to si scr control. (E) Enrichment score (ES) plots from GSEA using a published 2C-L gene set and RNA-seq data from the 3p and 5p siRNA-treated mESC samples used in Figure 4. (F) Representative FACS analysis of the proportion of mESCs expressing a MERVL-GFP reporter in the different siRNA-treated cells and including siRNA to *Chaf1* as a positive control. Percentage of MERVL-GFP+ cells based on Flo-Jo analysis is shown in red. (G) Proportion of MERVL-GFP+ cells in the different siRNA conditions relative to the siChaf1 positive control. Data are represented as mean ± SEM and statistical analysis as before and is from four independent experiments.

We first investigated whether 2C-LCs, which naturally arise within mESC populations, express *Stag1* NΔ isoforms. To formally address this, we obtained mESCs expressing a Dox-inducible *Dux-HA*-expression construct together with a MERVL-linked GFP reporter (Hendrickson et al., 2017). Dux is a 2C-specific transcription factor that binds to MERVL elements to activate expression (Hendrickson et al., 2017). We induced *DuxHA* expression in the MERVL-GFP mESCs and performed 5′ RACE as before on sorted GFP+ (2C-L) and GFP– cells (Figure 6A). We enriched several of the previously identified N-terminal truncated *Stag1* transcripts in the GFP+ population including e2/3Δ and e5Δ isoforms (Figure 6A, blue arrows). Importantly, we also identified a transcript starting at e7, similar to the one previously found in 5p KD mESCs (Figures 6B, 3A, and 3B). Remarkably, however, the sequence preceding the TSS in e7 in *Dux*-induced cells was an MT2-MERVL element, creating a chimeric, LTR-driven *Stag1* transcript, reminiscent of other LTR transcripts specifically expressed in the 2C-L state.

2C-LCs are a rare subpopulation that spontaneously arise in mESC cultures and exhibit unique molecular and transcriptional features (Eckersley-Maslin et al., 2016; Ishiuchi et al., 2015; Macfarlan et al., 2012). Given that 2C-LCs expressed several N-terminal truncated *Stag1* isoforms, we investigated whether these in turn supported the maintenance or emergence of that state. We treated mESCs with the panel of siRNAs and used qRT-PCR to test expression of candidate genes. We found that *Dux*, and consequently MERVL and other markers of the 2C-LC state, *Gm6763*, *AW822073*, and *Gm4981*, are strongly upregulated by 5p KD (Figures 6C, 6D, and S6A). Notably, all 2C-L genes analyzed remained unchanged in 3p KD conditions with a modest upregulation in SP KD. Furthermore, GSEA using a published 2C gene set (Percharde et al., 2018) revealed a specific enrichment among the upregulated genes in 5p KDs that was not observed in 3p KDs (Figures 6E and S6B), consistent with the different ends of *Stag1* targeting different RNA pools.

To functionally validate the expression results, we returned to the Dox-inducible *Dux-HA*, MERVL-GFP mESCs (Hendrickson et al., 2017) and used flow cytometry to directly measure the number of GFP+ cells in our different *Stag1* KD conditions (Figures 6F and 6G). Chaf1 is a chromatin accessibility factor previously shown to support conversion of mESCs toward totipotency (Ishiuchi et al., 2015). In support of the upregulation of the 2C-LC gene set in 5p KD mESCs, we observed an 8- to 9-fold increase in the proportion of GFP+ cells in 5p KD conditions compared with scramble-treated controls, similar to the published effect of Chaf1 KD (Figures 6F and 6G). There was a modest, but insignificant increase in GFP+ cells upon SP KD and no effect upon 3p KD. mESCs treated with both Chaf1 and 5p siRNAs had an additive effect on the proportion of GFP+ cells, suggesting that the two proteins function in complementary pathways for conversion toward 2C-LCs. Thus, 2C-LCs express N-terminal truncated *Stag1* isoforms, which in turn supports the maintenance or emergence of that state through rRNA repression and nucleolar changes. Together our results reveal a new and specific role for the N-terminus of STAG1 in the regulation of the 2C-LC state.

## Discussion

Most studies of cohesin function focus on the core trimer, despite the fact that it is the regulatory STAG subunit that is a pan-cancer target (Leiserson et al., 2015) and has clear roles in cell identity control (Viny et al., 2019). How these proteins contribute to cohesin’s functions, why cells have diversified them so extensively, and how their mutations lead so often to disease are poorly understood. Here, we reveal a novel role for *Stag1*, and in particular its unique N-terminal end, in regulating nucleolar integrity and 2C repression to maintain mESC identity. It has been known for a long time that several STAG paralogs exist in mammalian cells and that they have non-reciprocal functions with respect to chromosome structure and cohesion. By dissecting the diversity of naturally occurring *Stag1* isoforms in mESCs, we have shed new light not only on the unique divergent ends of the STAG paralogs but also the critical role that their levels play in cell fate control. Our results highlight the importance of careful understanding of chromatin regulators in cell-specific contexts.

*Stag1* knockout (Stag1^Δ/Δ^) ESCs give rise to mice that survive to E13.5 (Remeseiro et al., 2012a, 2012b). At first this observation seems at odds with our report that *Stag1* is required for pluripotency. However, our observations may in fact explain why the *Stag1*^Δ/Δ^ mouse model does not exhibit early embryonic lethality. In this model, only the 5′ region of *Stag1* was targeted, meaning that the *Stag1* isoforms lacking the N-terminus may still be retained in the targeted ESCs. This is consistent with our results showing that 5p KD cells have not lost their ability to self-renew nor is their pluripotency gene signature affected. It further suggests that changes to the nucleolus may exist in these cells. We acknowledge that, while the N-terminus is important for regulation of totipotency *in vitro*, it is possible that it may be dispensable *in vivo* during early development and future work in mice could resolve this.

The nucleolus is held together by liquid-liquid phase separation (PS), which is driven by the association of rDNA with nucleolar proteins and is dependent on continual rRNA synthesis (Feric et al., 2016; Yao et al., 2019). However, in one- to two-cell embryos, nucleoli lack distinct compartments, and exhibit low rRNA synthesis and low translation (Borsos and Torres-Padilla, 2016). Similarly, changes to rRNA synthesis or nucleolar PS are sufficient to convert ESCs toward the 2C-LC state, either through Dux dissociation from the nucleolar periphery and consequently its de-repression (Xie et al., 2022) or p53-mediated nucleolar stress (Grow et al., 2021). Other proteins including the NCL/TRIM28 complex (Percharde et al., 2018) and nucleolar LIN28 (Sun et al., 2021) have been shown to contribute to nucleolar integrity and repress DUX expression. In this context, our results position STAG1, and specifically its N-terminal end, as a novel regulator of the 2C-ESC transition through the control of nucleolar integrity. STAG1 is localized to the nucleolar periphery and interacts with the nucleolar proteins NCL/TRIM28 as well as being bound to and supporting rDNA and LINE-1 element expression. Our results suggest that the N-terminus of STAG1 plays a specific role in repressing conversion to the 2C state. STAG1 may contribute to nucleolar structure and function via both the regulation of rRNA expression as well as by supporting nucleolar PS through interactions with nucleolar regulators. In this context, modulating the availability of the N- or C-terminus of STAG1 may be a way in which ESCs impact nucleolar structure and function and thus cell identity. Our results also point to the different ends of STAG1 interacting with different protein partners since mESCs retaining the C-terminus of STAG1 do not exhibit changes to the nucleolus and do not convert into 2C-LCs. This is also supported by the different gene expression programs affected in the KDs that select for N-terminalΔ or C-terminalΔ isoforms. It may in fact be quite important for ESCs to express a diversity of alternative *Stag1* isoforms to support the plasticity of nucleolar structure and a range of cell fate options from totipotency to primed pluripotency.

Finally, *Stags* are commonly mutated in cancers (Leiserson et al., 2015). Our results point to misregulation of STAG proteins as leading to changes in epigenetic regulation that move beyond changes to TADs and protein-coding genes. Instead, they support a role for hierarchical changes to chromatin organization, nucleolar structure and function, and repeat deregulation in cell fate determination. Careful analysis of *Stag2*-mutant cancers should shed light on these and deliver new insights into cancers that harbor these mutations.

## Experimental procedures

### Resource availability

#### Corresponding author

Further information and requests for resources and reagents should be directed to and will be fulfilled by the corresponding author, Suzana Hadjur (s.hadjur@ucl.ac.uk).

#### Materials availability

Mouse ESC lines generated in this study are available from the lead contact with a completed Materials Transfer Agreement.

### ESC culture, siRNA KD, and qRT-PCR

Male E14 mESCs were cultured in serum (FCS) or naive (2i) conditions. Serum-cultured cells were grown on 0.1% gelatin-coated plates in GMEM, 10% FCS (Sigma), NEAA, Na pyruvate, 0.1 mM β-mercaptoethanol (BMe), GlutaMAX, and freshly added LIF (1:10,000). 2i-cultured cells were grown on plates coated with fibronectin, in DMEM:F12/neurobasal 1:1, KnockOut Serum Replacement, N2, B27, GlutaMAX, 1 μM PD0325901, 3 μM CHIR9902, 0.1 mM BMe, and freshly added LIF as above. DuxHA/MERVL-GFP cells were cultured in 2i conditions. siRNAs were purchased from Horizon Discovery (previously Dharmacon) or Sigma (for “enzymatically derived” esiRNAs). siRNA KDs were performed for 24 or 72 h (for Figure 6) in 6-well plates where 200,000 cells were seeded for 72 h KDs, and 400,000 for 24 h KDs. siRNAs (50 pmol) were transfected using RNAiMax Lipofectamine at the time of seeding and, after 48 h for 72 h time points. Two siRNA controls were used, scrambled (scr) was D-001810-10 and Luciferase (esiLuc) Sigma. siSA1 SP was derived from equimolar ratios of commercial siRNAs (D-041989-02, -04, -05, -06, -07, or -08). siSA1 5p was a custom Duplex siRNA sequence (AGGAGCAGGUCGUGGAAGAUU). siSA1 3p was derived from equimolar ratios of commercial siRNAs J-041989-05, -07, or -08. esiRNA to SATS was a custom-made product (Sigma) to the entire SATS 5′ UTR (mm10 chr9:100,597,794–100,598,109). Total RNA was isolated using a Monarch RNA prep kit (NEB). Reverse transcription was performed on 0.5 μg DNase-treated total RNA using Lunascript RT (NEB) in 20-μL reactions. qPCR was performed using 2× SensiFAST SYBR No-ROX kit (Bioline) in 20 μL reactions using 1 μL of RT reaction as input and 0.4 μM of each primer.

### AP assay and quantification

Cells were seeded in 6-well plates and transfected with siRNAs as above. After 24 h, cells were collected for RNA isolation and KD efficiency analyzed by qRT-PCR. Cells from each condition were counted and 1,000 cells per well seeded into a new 6-well plate. Cells were re-transfected after 48 h using 5 pmol of siRNAs. Four days after seeding cells at clonal density, the cells were assayed for AP expression using a StemTAG Alkaline Phosphatase staining kit (Cell Biolabs CBA-300). AP-stained cells were imaged in 6-well plates using an M7000 Imaging System (Zeiss) with a 4× objective and a trans-illumination bright-field light source. For quantification, the area occupied from all colonies/conditions was assessed (using the area tool in ImageJ) and then, separately, the area of the dark purple/AP^hi^ colonies was assessed. To normalize for the number of colonies, the AP^hi^ area was expressed as a fraction of the total colony area (percent of total). The percentages are shown relative to 1 in the main figures for the heatmaps for each biological replicate separately.

### RACE and PCR mini screen

RACE was performed using GeneRacer kit (RLM RACE, Invitrogen L1500). Two micrograms of total RNA was used as input. Final products were amplified by nested PCR, using Kapa 2× MasterMix. First, PCR was carried out in a 50 μL reaction using 1 μL RT as input, 25 cycles. DNA was purified using a QIAGEN PCR Purification kit, and nested PCR was performed on a 10th of the first PCR for 30 cycles. The viewpoint for 5′ RACE was in exon 2 (Figure 3A) or exon 8 (Figure 3B) of Stag1. The viewpoint for 3′ RACE was in exon 23 (Figure 3C). RACE primer details can be found in Table S3. PCR products were excised from the gel, A-tailed using Klenow exo- (NEB) and cloned into pCR4-TOPO vector (Invitrogen). At least three clones were sequenced per PCR product. For the PCR Mini-Screen, forward primers at either SATS or canonical 5′ UTR were used with reverse primers either at the end of Stag1 canonical coding sequence, or at the end of coding sequence in intron 25 (see Table S3). PCR was performed using Kapa 2× MasterMix. DNA was excised from the gel, A-tailed, and cloned into pCR4-TOPO. At least six clones per PCR product were Sanger sequenced. Sequences from the PCR Mini-screen were aligned using Minimap2 (2.14-r884) in “splice” mode to ensure long read splice alignment (Figures 3D and S3A).

### Protein analysis including WBs, coIP, and IF

Please see supplemental experimental procedures where these are described in detail.

### Nascent transcription and translation analysis

For nascent transcription analysis, we used the Click-iT RNA Alexa Fluor 488 HCS Assay (Invitrogen C10327). Cells were labeled with 1 mM EU for 45 min at 37°C in fresh medium. Cells were fixed in solution or onto coverslips with 3.7% paraformaldehyde and permeabilized with 0.5% Triton X-solution. Cells were incubated with the Click-iT reaction cocktail for 30 min and then either processed further for IF (directly to the blocking step) or analyzed by flow cytometry on a BD Fortessa X20. For the nascent translation analysis, a Click-iT HPG Alexa Fluor 594 Protein Synthesis Assay Kit (Invitrogen C10429) was used. Cells were pre-incubated at 37°C in methionine-free medium for 30 min before addition of HPG at 50 μM. Cells were incubated with HPG for 30 min, then collected, fixed, permeabilized, and stained using Click-It reaction in low retention tubes. HPG incorporation was measured by flow cytometry. FACS analysis was done with FloJo software (v.10.7.1).

### RNA-seq library preparation and sequencing

ESCs were treated for 24 h with siRNA pools to *Stag1* and two sets of control siRNAs, scrambled (SCR), and Luciferase (Luc). There are three replicate sets for SP KD and two for the siRNA pools (SATS, SP, 3p, and 5p). Total RNA was isolated using an NEB Monarch RNA prep kit. One microgram of total RNA was rRNA-depleted using an NEBNext rRNA depletion kit (human/mouse/rat). Libraries were prepared from 10 to 50 ng rRNA-depleted total RNA using an NEBNext Ultra II directional RNAseq kit according to the manufacturer’s instructions using eight cycles of PCR. All ESC FCS libraries were rRNA depleted and the ESC 2i libraries were poly(A) enriched (with two rounds of enrichment). RNA-seq libraries were sequenced on the Illumina HiSeq3000 platform, with 75 bp paired-end or single-end reads. Reads were quality controlled using FASTQC. RNA-seq data were processed using the RNA-seq Nextflow pipeline (v.19.01.0), with the following parameters: –aligner hisat2 –genome mm10, with –reverse_stranded specified for paired-end samples. FeatureCounts output was parsed through edgeR (v.3.16.5) and DESeq2 (v.1.14.1) to generate normalized expression counts. The normalized counts for RNA-seq (Figure 1) were calculated in edgeR. Low-expressed genes were removed (rowSum cpm <2 across SCR and SA1SP replicates), normalization factors were calculated using calcNormFactors, and dispersions estimated using estimateDisp. The edgeR volcano plot statistics were calculated using the exactTest and topTags functions. To generate the normalized counts for RNA-seq experiments required to calculate the log2FC GSEA ranked lists, the FeatureCounts output for all experiments was combined into a single table and read into DESeq2. A DESeq2 object was built using the function DESeqDataSetFromMatrix and estimation of size factors and dispersions were calculated using the DEseq function. Normalized counts were calculated using the “counts” function. Low-expressed genes (rowSum normalized count <10 across all samples) were removed. See supplemental experimental procedures for detailed information about GSEA and VAST-tools.

### PacBio library, sequencing, and analysis

ESCs were cultured in naive 2i conditions, and poly(A)-enriched mRNAs were hybridized to a custom biotinylated oligonucleotide probe set. Post-capture, mRNAs were amplified using the Clontech SMARTer PCR cDNA Synthesis Kit with nine cycles and used in the SMRTbell library prep according to the manufacturer’s instructions. The library was sequenced on the SMRTseq 2000 platform. PacBio reads were processed through the SMRTLINK v.8.0.0 IsoSeq3 pipeline. A total of 403,995 circular consensus sequences (CCSs) were generated using default parameters (--minPasses = 1, --min-rq = 0.8, CCS Polish = No). Further refining through lima (removal of adapters and correct orientation of sequences), poly(A) trimming, and concatamer removal resulted in 265,106 full-length non-chimeric (FLNC) reads. FLNC reads were aligned to the mm10 genome using Minimap2 with the following parameters (-ax splice, -uf, -k14).

### ChIP-seq analysis

Previously published STAG1 ChIP-seq datasets from ES 2i cells (GSE126659) were trimmed using trim_galore and aligned to mm10 using bowtie2. Peak detection was performed with MACS2 using unique reads (MAPQ ≥ 2). Peaks were overlapped with genomic features in a hierarchical manner (promoters > exons > repeats > introns > intergenic), and overlap frequency was compared with a randomly shuffled version of the peaks. To identify repeat families enriched for STAG1 peaks, a previously described pipeline was used (Deniz et al., 2020) that compares family-levels overlap frequency with that observed in 1,000 permutations of random peak shuffling. Coverage profiles across specific TE families were generated using HOMER and including multi-mapping reads (MAPQ < 2).

### UMI-4C and Hi-C

Please see the supplemental experimental procedures where these are described in detail.

## Data Availability

Genomic data generated in this study are available at GEO with the Accession GSE160390.

## Supplemental information

Document S1. Supplemental experimental procedures, Figures S1–S5, and Tables S1–S3

Document S2. Article plus supplemental information
