# Supplementary material for: The N-terminus of Stag1 is required to repress the 2C program by maintaining rRNA expression and nucleolar integrity
Source: Stem Cell Reports. 2023 Oct 5;18(11):2154–73. doi: 10.1016/j.stemcr.2023.09.004 (PMC10679541; doi:10.1016/j.stemcr.2023.09.004)
Supplement: Document S1. Supplemental experimental procedures, Figures S1–S5, and Tables S1–S3 [file mmc1.pdf]

Stem Cell Reports, Volume *18*

Supplemental Information

The N-terminus of *Stag1* is required to repress the 2C program by maintaining rRNA expression and nucleolar integrity

Dubravka Pezic, Samuel Weeks, Wazeer Varsally, Pooran S. Dewari, Steven Pollard, Miguel R. Branco, and Suzana Hadjur

Supplementary Figure 1. *Pezic et al.*

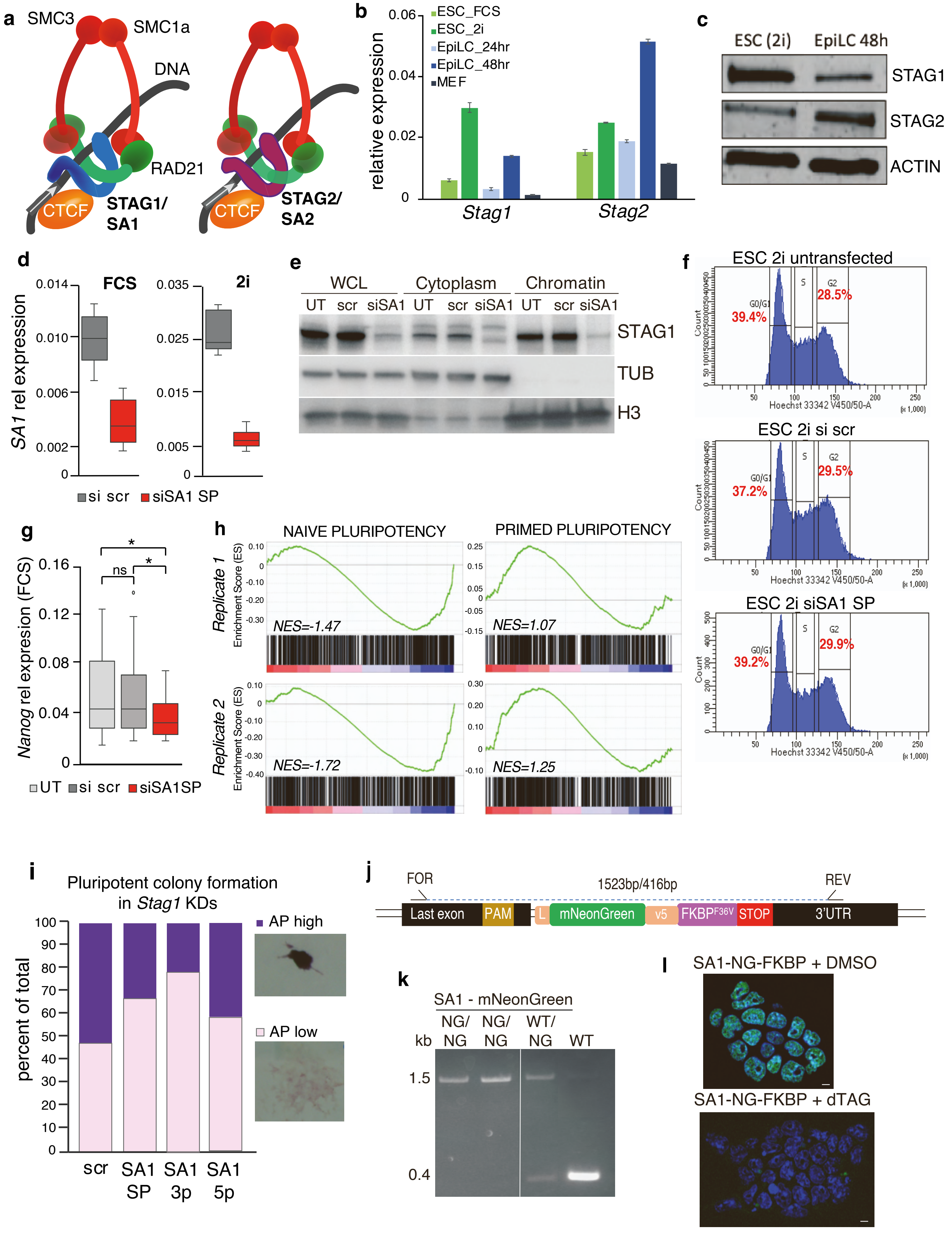

Supplementary Figure 2. *Pezic et al.*

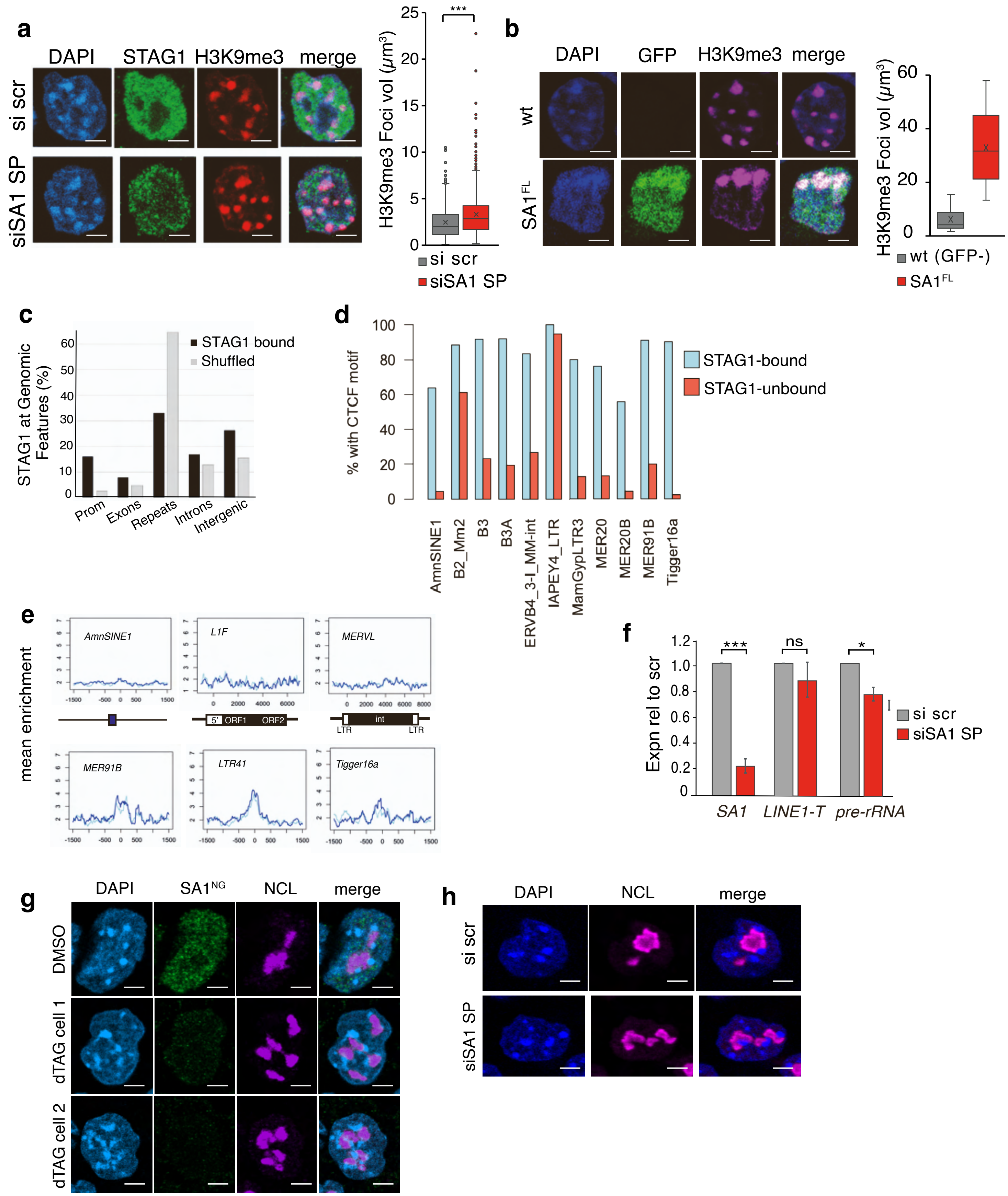

Supplementary Figure 3.

Pezic et al.

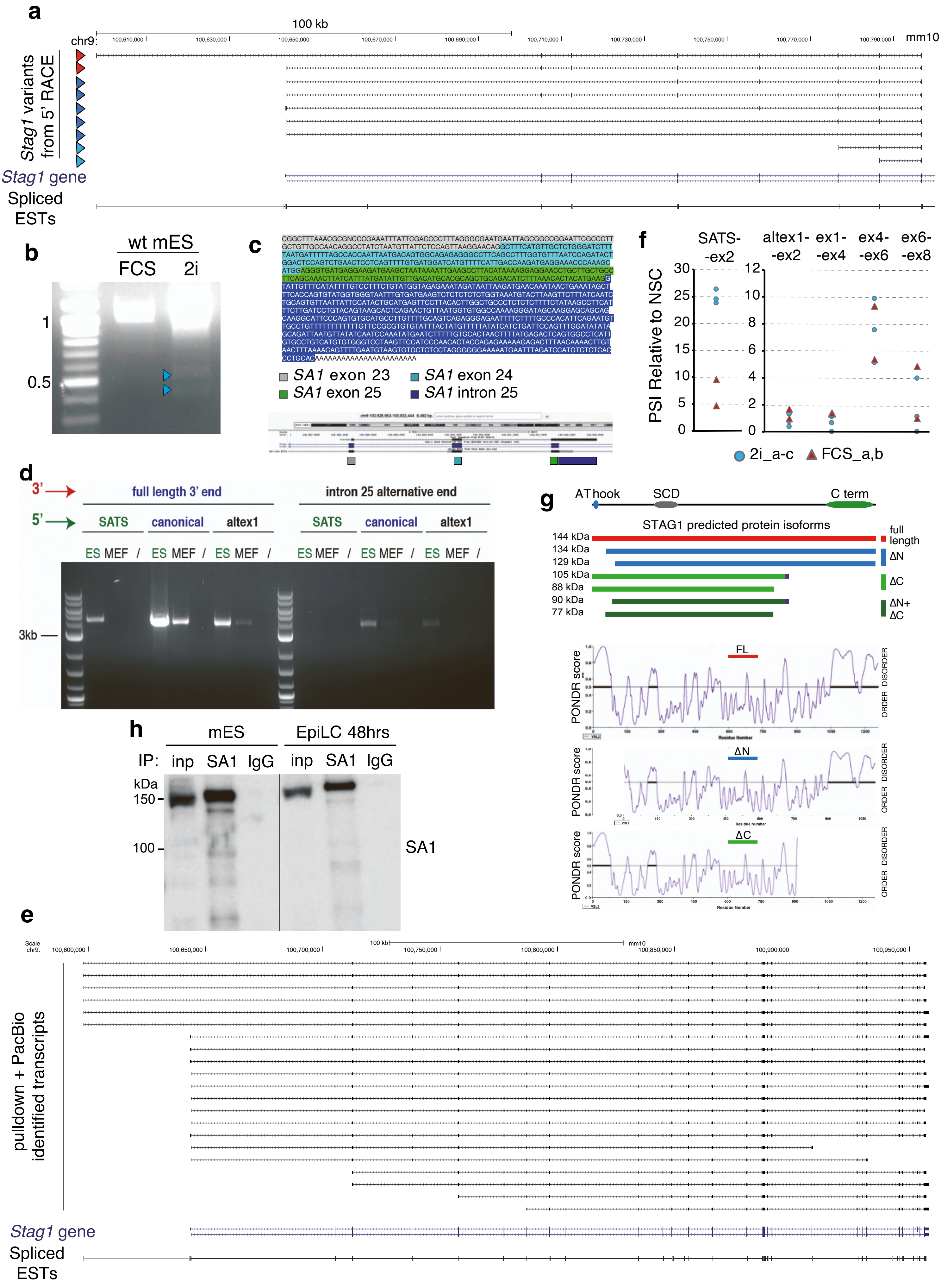

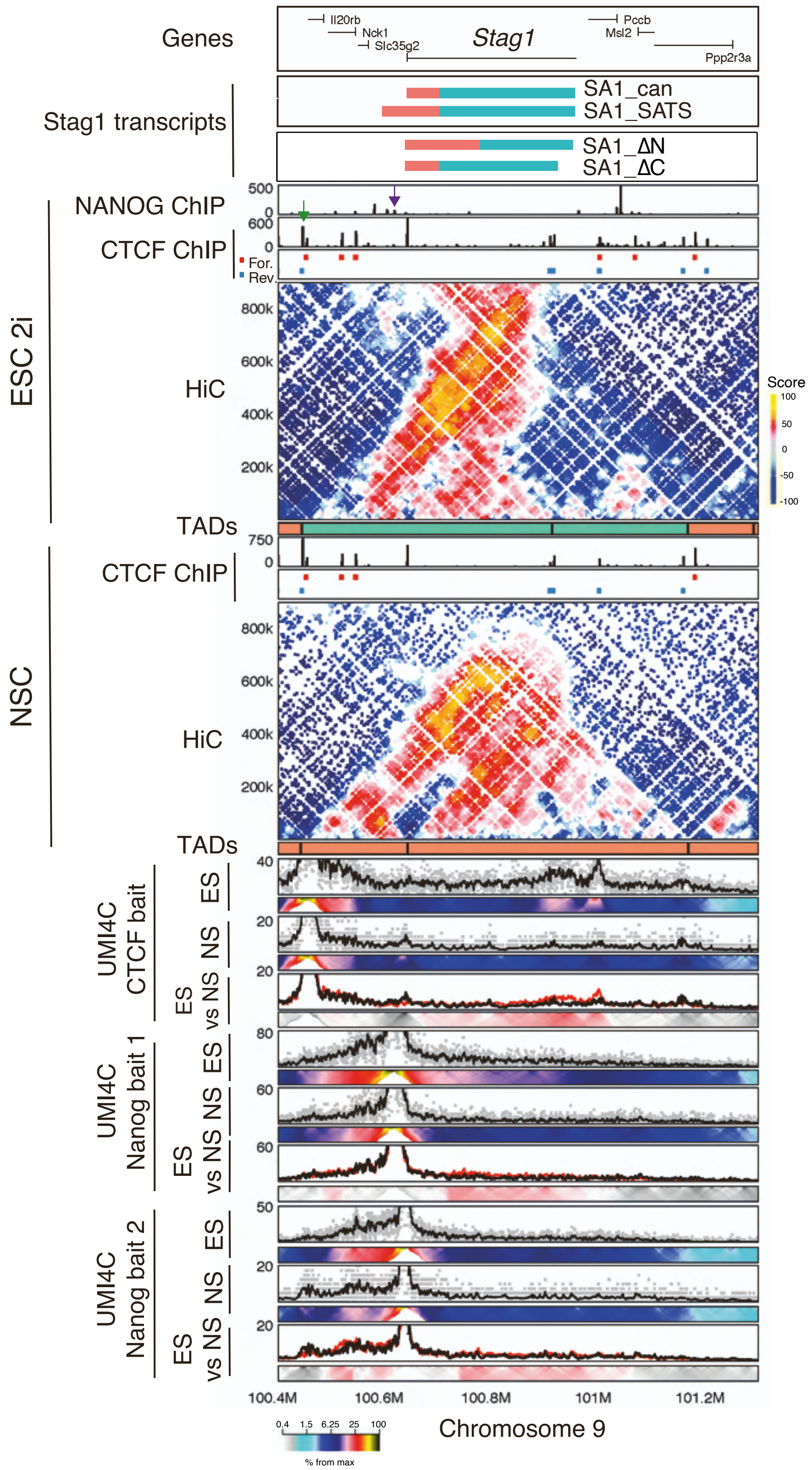
Genes
Il20rb
Nck1
Slc35g2
Stag1
Pccb
Msl2
Ppp2r3a
Stag1 transcripts
SA1_can
SA1_SATS
SA1_ΔN
SA1_ΔC
ESC 2i
NANOG ChIP
CTCF ChIP
For.
Rev.
HiC
TADs
Score
NSC
UMI4C CTCF bait
UMI4C Nanog bait 1
UMI4C Nanog bait 2
ES
NS
ES vs NS
% from max
100.4M
100.6M
100.8M
101M
101.2M
Chromosome 9

Supplementary Figure 5. *Pezic et al.*

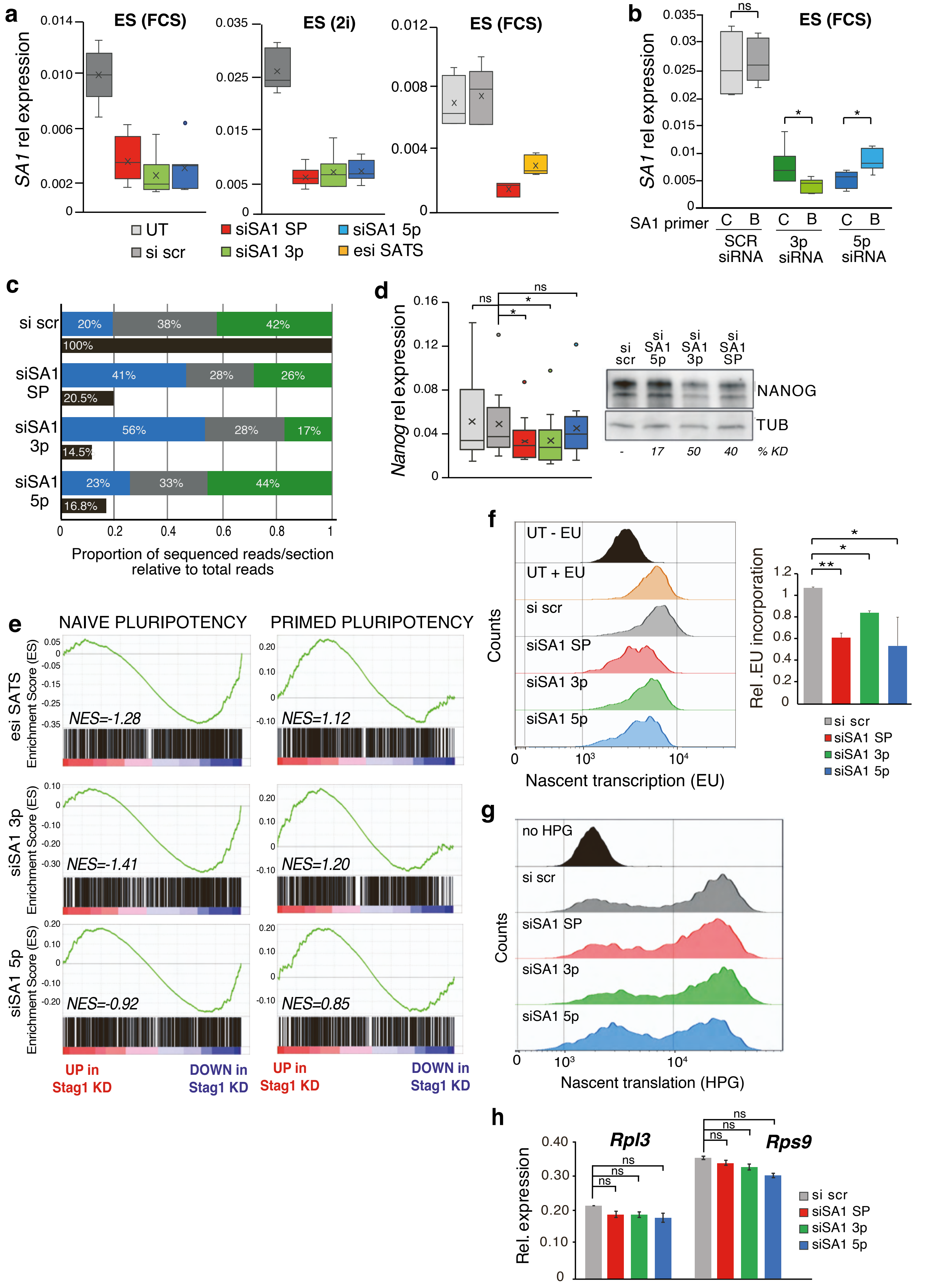

Supplementary Figure 6. *Pezic et al.*

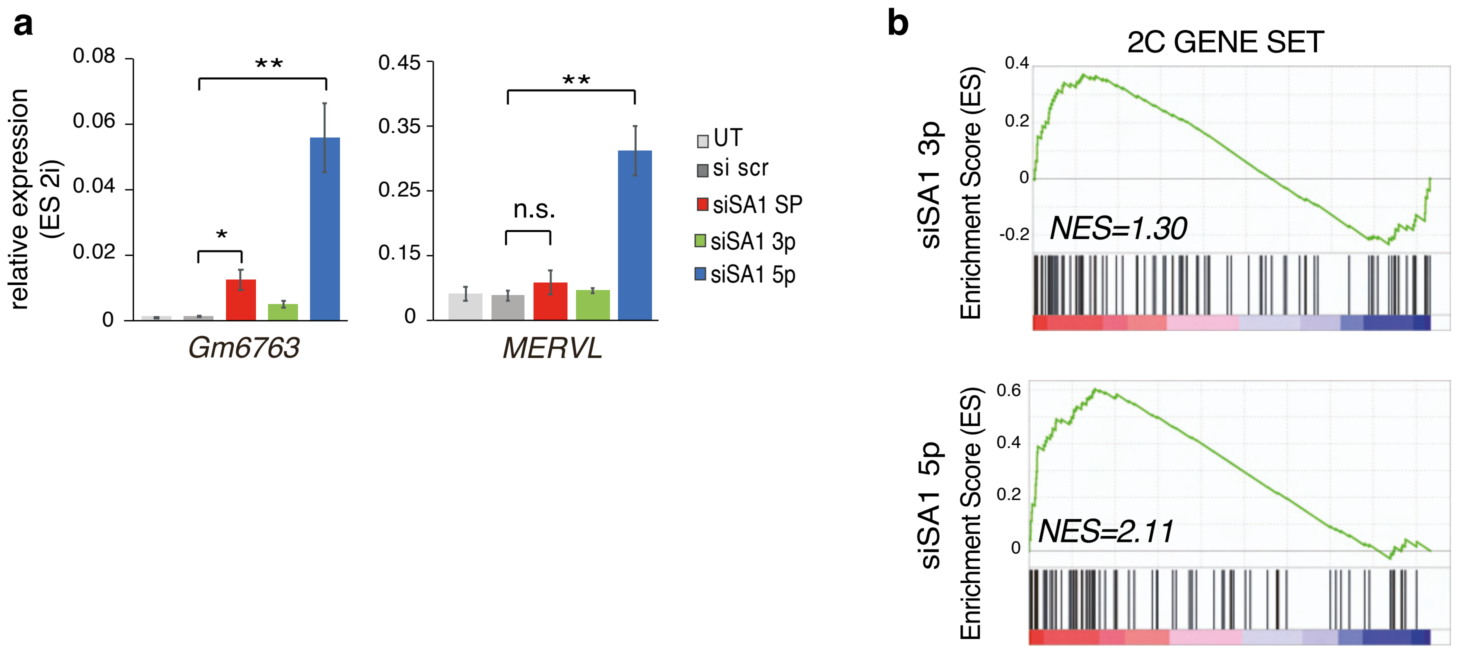

SUPPLEMENTARY FIGURE LEGENDS

Supplementary Figure 1. *Stag1* is required for pluripotency in mESCs. Related to Figure 1.
a) Cartoon of the cohesin complex including the core trimer subunits of SMC1a, SMC3 and RAD21 complexed with either STAG1 or STAG2.
b) Relative expression of *Stag1* and *Stag2* mRNA by qRT-PCR in 2i- (naïve) or FCS-grown mESC, EpiLCs and MEFs. Data is represented as mean ± SEM of two independent experiments and relative to *Actin* expression.
c) WCL from naïve (2i) mESC and EpiLCs, sorted for cells in the G1 phase and analysed by WB for levels of STAG1 and STAG2. ACTIN serves as a loading control.
d) Relative expression of *Stag1* mRNA by qRT-PCR in FCS- (left panel, n=20) or 2i-grown (right panel, n=19) mESCs upon treatment with si scr or si SA1, smartpool (SP). Whiskers and boxes indicate all and 50% of values respectively. Central line represents the median. Data is n=10 (FCS) and n=8 (2i) independent experiments.
e) WB analysis of STAG1 levels in WCL, cytoplasmic and chromatin fractions upon treatment with scrambled siRNAs (si Scr) or SA1 siRNAs (siSA1) for 24hr in naïve mESCs. Data as in Fig. 1c, but also including the cytoplasmic fraction. Tubulin (TUB) and H3 as controls.
f) Cell cycle analysis of Hoechst-stained 2i mESC after treatment with si scr or siSA1siRNAs for 24hrs. Shown are the percentages of cells in G1 or G2 phases. These are the same cells that were used for the RNA-sequencing experiments shown in Fig. 1.
g) Relative expression of *Nanog* mRNA by qRT-PCR in FCS-grown mESCs upon treatment with si scr or siSA1. Quantification and statistics as before. Data is from five independent experiments.
h) Enrichment score (ES) plots from GSEA using the naïve or primed gene sets as in Fig. 1 and RNA-seq from two additional siSA1 mESC biological replicates. The third replicate is shown in Fig. 1.
i) AP^{hi} colonies in mESCs (purple), as a percentage of all colonies (pink and purple) treated with the siRNA panel. Data are the average of three independent replicates.
j, k) Cartoon of the CRISPR/Cas9 targeting strategy to introduce a NeonGreen-v5-FKBP tag to the C-terminus of endogenous *Stag1*. FOR and REV primers used for genotyping. The leftmost NG/NG homozygous sample represents the 'B1 clone' used in the manuscript.
l) Representative confocal images of neon-green in $SA1^{NG\text{-}FKBP}$ mESC (clone B1) treated with DMSO or dTAG. Scale bar, 3 microns.

Supplementary Figure 2. STAG1 is localised to and impacts both euchromatin and heterochromatin compartments. Related to Figure 2.
a) Left, representative confocal images of IF to STAG1 and H3K9me3 in siRNA-treated mESC counterstained with DAPI. Right, Imaris quantification of the volume of H3K9me3 foci from siRNA treated mESC. Quantifications and statistical analysis were done as above. Data is from n>140 independent cells/condition in three biological replicates. Scale bar, 3 microns.
b) Left, representative confocal images of IF to GFP and H3K9me3 in mESCs expressing a dox-inducible GFP-tagged full-length STAG1 (SA1-FL) and counterstained with DAPI. Right, Imaris quantification of the volume of H3K9me3 foci from dox-inducible mESCs. Quantifications and statistical analysis as above. Data is from n>80 independent cells/condition in two replicates.
c) Percentage of STAG1 ChIP-seq peaks in mESCs (unique reads) at promoters, exons, transposable element repeats, introns and intergenic sequences.
d) Analysis of CTCF motifs contained within selected repeat elements and the percentage of STAG1 binding. NB. The majority of elements contain both a CTCF motif and STAG1.
e) STAG1 ChIP-seq (unique and multimapping reads) aligned to additional full-length repeat elements. Two STAG1 ChIP replicates are shown in blue alongside the INPUT in grey.

f) Relative expression of *Stag1*, *LINE1-T* and *pre-rRNA* by qRT-PCR in siRNA-treated mESCs. Shown are total RNA levels. Data is represented as mean ± SEM and statistical analysis as before. Data is from three independent experiments.

Representative confocal images of MFI of NCL assessed by IF in g) $SA1^{NG\text{-}FKBP}$ mESC (clone B1) treated with DMSO or dTAG (two different cells shown) and h) siRNA-treated mESC and counterstained with DAPI. Quantification of these is shown in Fig. 2k. Scale bar, 3 microns.

Supplementary Figure 3. Transcription-regulatory control of *Stag1* in mESC. Related to Fig 3.

a) Aligned *Stag1* transcript variants identified from 5'RACE in Fig. 3a. Arrows refer to the bands on the RACE gels which were cloned and sequenced. *NB,* the diversity of skipping events that all result in a functional loss of the 5' end of *Stag1*.

b) Over-exposure of 5'RACE gel shown in Fig. 3a (right) to show small RACE products (blue arrows).

c) Close-up of the 3' RACE sequence that identified a new alternative TTS in intron 25 (sequence shown in dark blue).

d) PCR mini-screen in naïve mESCs and MEFs using various combinations of forward (5') primers (SATS, canonical TSS, Alt exon 1 TSS) and reverse (3') primers (canonical TTS, Alt intron 25 TTS). *NB.* SATS is only expressed in ESC; canonical, full-length *Stag1* is more expressed in ESC compared to MEFs; and the alternative intron 25 TTS is most often expressed with a canonical TSS.

e) *Stag1* transcripts sequenced on the PacBio platform. Including many isoforms that were already discovered using RACE and PCR cloning methods above.

f) Percent Spliced In (PSI) calculations based on VAST-Tools analysis of RNA-seq from multiple 2i (blue) and FCS (red) datasets (see Methods). Data are shown relative to Neural stem cell (NSC) frequencies, highlighting the events that are ESC-specific.

g) Top, cartoon depicting functional domains within STAG1 protein, including the AT-hook (aa 3-58); Stromalin conserved domain (SCD, aa 296-381) and the C-terminus. Middle, the predicted STAG1 protein isoforms based on transcript analysis with estimated sizes for each isoform and colour coded according to the analysis in Fig 3d. Purple boxes in the 105kDa and 90kDa isoforms represent retained introns. Bottom, PONDR (Predictor of Natural Disordered Regions) analysis of STAG1 using VSL2 predictor showing consecutive stretches of disordered regions corresponding to the N- and C-terminus of STAG1 in its full-length (FL), N-terminal (ΔN) and C-terminal delta (ΔC) isoform groups.

h) Chromatin Immunoprecipitation of endogenous STAG1 in mESCs and EpiLCs. IgG was used as a control. *NB.* Both canonical and STAG1 isoform levels are reduced upon differentiation.

Supplementary Figure 4. Genome topology at the *Stag1* locus. Related to Figure 3.

Hi-C contact maps in naïve mESC and NSC of the 900kb region on chromosome 9 containing the *Stag1* topologically associated domain (TAD). TADs are denoted with a vertical line and as repressed (orange) or active (blue). Shown also are tracks for NANOG and CTCF ChIP-seq as well as a track indicating the directionality of CTCF binding sites (red, forward; blue, reverse). Aligned to the gene track are also the *Stag1* transcripts discovered above where red represents the untranslated regions and blue the coding body. UMI-4C-seq viewpoints are positioned to the leftmost CTCF site ('CTCF bait', vertical green arrow on ChIP track) and to a Nanog site 40 kb upstream of the *Stag1* canonical TSS ('Nanog bait', vertical purple arrow on ChIP track). For each bait, UMI information for each cell type is shown as well as the comparative plots where red represents an enrichment of contacts in ESC compared to NSC.

Supplementary Figure 5. Fluctuations in *Stag1* isoforms skews cell fates. Related to Figure 4.

a) Relative expression of *Stag1* mRNA by qRT-PCR using SA1_A primer in FCS- (n=7) or 2i-grown (n=6) mESC upon si scr or the si SA1 panel. Quantifications as before.

b) Relative expression of *Stag1* mRNA by qRT-PCR using two different primers located at either the 5' end (SA1_C) or the 3' end (SA1_B) of *Stag1* mRNA in mESCs (n=6) treated with scr, 3p or 5p siRNAs. While both siRNA sets reduce *Stag1* to a similar amount overall, the relative proportion of the residual 5' or 3' ends is significantly different in the 3p or 5p KDs. * p<0.05.
c) Data as in Fig. 4c shown here for the second biological replicate siRNA KD panel (same samples as the GSEA in Fig S5e).
d) Left, relative expression of *Nanog* mRNA by qRT-PCR in FCS-grown mESCs upon si scr or the si SA1 panel (n=13). Quantifications as before. *NB,* the modest, but different influence of the 5p and the 3p KDs on *Nanog* levels. Right, WB analysis of NANOG levels in siRNA treated mESC WCL. TUB is loading control. The percentage of KD of NANOG signal normalised to TUB is shown.
e) Enrichment score (ES) plots from GSEA using the naïve or primed gene sets as in Fig. 1f and RNA-seq data from the second set of mESCs treated with the siRNAs to SATS TSS, 3p and 5p.
f) Global analysis of nascent transcription by measuring EU-488 incorporation using Flow cytometry. Left, representative Flo-Jo analysis of EU incorporation in mESCs treated with the siRNA KD panel and controls. Right, quantification of the change in EU incorporation relative to si scr treated cells. Data are represented as the mean +/- SEM and are from three independent replicates. Statistical analysis using two-tailed t-test.
g) Global analysis of nascent translation by measuring HPG incorporation using Flow cytometry. Shown is representative Flo-Jo analysis of HPG incorporation in mESC treated with the siRNA KD panel. Quantifications of the data can be found in Fig. 5f.
h) Relative expression of *Rpl3* and *Rps9* mRNA by qRT-PCR in mESC upon si scr or the si SA1 panel. Data are represented as the mean +/- SEM and are from three independent replicates. Statistical analysis using two-tailed t-test.

Supplementary Figure 6. Loss of the *Stag1* N-terminus leads to conversion to totipotency.
a) Relative expression of 2C related genes by qRT-PCR in 2i-grown mESC after treatment with si scr or the si SA1 panel. Data are represented as mean +/- SEM from n=5 independent replicates.
b) Enrichment score (ES) plots from GSEA using 2C gene sets as in Fig. 6e and the replicate RNA-seq data from the siRNA treated mESC samples.

SUPPLEMENTARY METHODS and TABLES

GSEA - Broad Institute GSEA Preranked (v4.0.3) was used to determine the enrichment of curated genesets within our RNA-seq data. For each sample a ranked list was generated with genes ranked in descending order by their log2FC value using normalised expression scores from DEseq2. Log2FC per gene was calculated between the KD and its respective SCR using the following calculation: Log2(normalised_counts KD +1) - log2(normalised_counts SCR +1). In the case of experiments with multiple KD replicates, the average log2 normalised count was used. Three gene sets were assayed in this study, 'naïve pluripotency', 'primed pluripotency' and '2C signatures'. The naïve and primed pluripotency gene sets were curated in-house from (Fidalgo et al., 2016) where genes were selected if they had $\geq$2 fold change. The naïve and primed gene sets contained 661 and 580 genes respectively. The 2C signatures gene set (147 genes) was obtained from (Percharde et al., 2018). Gene sets were classed as having significant enrichment if the p-value was $\leq$0.05 and the normalised enrichment score (NES) exceeded +/- 1.

CRISPR-Mediated *Stag1* Knock-in Cell Line Generation - The guide RNA targeting Stag1 3' terminal coding region was designed using Tagin Software (http://tagin.stembio.org) and purchased from IDT. Lyophilised gRNA was rehydrated in RNA duplex buffer (100μM). The single stranded

oligodeoxynucleotides (ssODN) encoding mNeonGreen (mNG)-V5-FKBP12^{F36V} and the left and right homology arms was designed using the software tool ChopChop (https://chopchop.cbu.uib.no) and purchased as a High-Copy Amp-resistant plasmid from Twist Bioscience. 2.2μl gRNA (100μM) was mixed with 2.2μl tracrRNA ATTO 550nm (IDT) and annealed together. The RNA duplex was then incubated with 20μg S.p Cas9 Nuclease V3 (IDT) for 10min at room temperature and stored on ice prior to transfection. Linearised KI sequence was mixed with 100% DMSO and denatured at 95°C for 5min. The ssODN was plunged immediately into ice. The RNP complex was mixed with confluent 2i-grown ES cells re-suspended in P3 transfection buffer (Lonza) before being transferred to an electroporation microcuvette well (Lonza). Transfection was performed using a 4D Amaxa electroporator. Post-nucleofection, the cells were seeded into a fibronectin-coated 6 well plate with fresh ESC media. The media was changed daily for four days before being expanded into a T75 flask. Confluent ESC were FACS sorted for GFP+ population (BD FACS Aria Fusion Cell Sorter) and sparsely seeded into 10 cm plates. Clones were manually picked into 96 well plates and expanded for selection by v5 IF, genotyping and Sanger sequencing.

Dox-inducible STAG1-GFP isoform cell lines - Stag1 isoforms were cloned into pCW57.1 vector (Addgene 41393), modified using Gibson assembly to include an EGFP tag at the 3'end of the Gateway cassette, using Gateway recombination by LR clonase. For primers used to clone the isoforms see Supplementary Table S3. Plasmids were transfected into 2i-grown ESCs using Lipofectamine 3000 and cells grown in Puromycin-supplemented media (1μg/ml) for ten days to make stable lines. Isoform expression was induced using 2μg/ml Doxycycline for 24 hrs, and the population enriched for GFP-positive cells using FACS. For IF experiments, induction with Dox was for 48 hours.

VAST-TOOLS - To generate Percent Spliced In (PSI) scores, a statistic which represents how often a particular exon is spliced into a transcript using the ratio between reads which include and exclude said exon was used (Tapial et al., 2017). Paired-end RNA-seq datasets were submitted to VAST-TOOLS (v2.1.3) using the Mmu genome. Briefly, reads are split into 50nt words with a 25nt sliding window. The 50nt words are aligned to a reference genome using Bowtie to obtain unmapped reads. These unmapped reads are then aligned to a set of predefined exon-exon junction (EJJ) libraries allowing for the quantification of alternative exon events. The output was further interrogated using a script which searches all hypothetical EEJ combinations between potential donors and acceptors within Stag1. PSI scores could be obtained providing there was at least a single read within our RNAseq data that supported one of these potential events. Some datasets were combined to have enough reads for the analysis. See Table S1.

Table S1. Percent Spliced In (PSI) Values

Source of RNA-seq data	Name in Figure	SATS-exon2	AltEx1 - exon2	exon1 - exon4	exon4 - exon6	exon6 - exon8	exon21 - AltEx22
this study, 'ES 2i_Rep1_enriched'	2i_a	23.68	5.26	1.29	10.34	1.03	0
this study, 'ES 2i_Rep1'	2i_b	21.43	9.52	0	7.89	0	0
doi.org/10.1016/j.stemcr.2018.08.001	2i_c	22.08	2.60	2.13	5.36	3.56	0
doi:10.1016/j.cell.2017.09.043	FCS_a	8.62	12.07	2.63	9.73	0	0.81
this study, 'ES FCS_Rep2/3_CTL/SCR' combined	FCS_b	4.25	6.95	2.58	5.63	0.91	1.11
doi:10.1016/j.cell.2017.09.043	NSC (rel to)	0.98	7.14	1.92	1.05	0	0

Quantifying sectioned *Stag1* and PONDR - Stag1 was split into 5 sections; SATS, e1-e8, e12-e19, e20-e25, e26-e34. Using Kallisto (v0.46.1), raw RNAseq reads were used to quantify each section of Stag1. Kallisto was run in quant mode, using the –rf-stranded parameter, outputting a TPM per Stag1 section. TPMs were normalised to the total reads from the 3 sections analysed and expressed as a percent. Internally disordered regions were predicted using VSL2 predictor at http://www.pondr.com.

Immunofluorescence and Microscopy - mESCs were cultured on fibronectin or gelatin-coated cover glass in 6-well plates. Cells were fixed in 4% Paraformaldehyde for 5min and incubated in 0.1% Triton X-PBS for 10min before being washed and blocked in 10% FCS-PBS for 20min. Primary antibodies were diluted in 10% FCS, 0.1% Saponin (Sigma) and incubated overnight at 4°C. The next day, the cells were incubated with an Alexa fluorophore-conjugated secondary antibody diluted in 10% FCS, 0.1% Saponin for 1 hr at room temperature, washed and mounted on cover slides with ProLong Diamond Antifade Mountant with DAPI (Invitrogen). Z-stacks imaging of fixed cells was done using a LSM 880 confocal microscope (Zeiss) with a 63X oil objective. Analysis was performed using Imaris 9.6 (Oxford instruments). Live cell imaging was performed using a 3i Spinning Disc confocal microscope (Zeiss). Stag1-mNG-V5-FKBP12^{F36V} cells were seeded in an 8-chambered coverglass (Lab-Tek II) and DMSO or dTAG (500nM) were added for 24hr before imaging. Directly prior to imaging, cells were incubated with Hoechst 33342 (BD Pharmingen) for 45min, and then replaced with fresh 2i ESC media. Cells were imaged as confocal Z-stacks using DAPI and GFP lasers with a 63X objective and 1.4 Numerical Aperture.

Antibodies - STAG1/SA1, N-term epitope (Abcam, ab4455); STAG1/SA1, C-term epitope (Abcam, ab4457); STAG2/SA2 (Bethyl, A300-158A); SMC3 (Abcam, ab9263); NANOG (Abcam, ab70482); Tubulin/TUB (Sigma, T5168); ACTIN (Novus, Mab8929); H3 (Abcam, ab1791); v5 (Invitrogen, 14-6796-82); HP1a (Cell Signalling, 2616); Nucleolin/NCL (Abcam, ab22758); POLR2 (Covance, MMS-128P); H3K9me3 (Abcam, ab8898); H3K4me3 (Abcam, ab8580); Alexa488-anti-GFP (GFP) (ThermoFisher, A-21311); TRIM28 (ThermoFisher, MA1-2023).

Protein Lysates, Fractionations and Western blotting - Whole cell lysates (WCL) were collected by lysis in RIPA buffer (150mM NaCl, 1% NP-40 detergent, 0.5% Sodium Deoxycholate, 0.1% SDS, 25mM Tris-HCl pH 7.4, 1mM DTT) and sonicated at 4°C for x5 30 second cycles using Diagenode Bioruptor. Insoluble material was pelleted and the supernatant lysate was quantified using BSA Assay (Thermo Scientific). For cellular fractionations, a cellular ratio of 5×10^6 cells/80μl buffer was maintained throughout the protocol. Cells were re-suspended in Cell Membrane Lysis Buffer (0.1% Triton X, 10mM HEPES pH 7.9, 10mM KCl, 1.5mM MgCl2, 0.34M sucrose, 10% glycerol, 1mM DTT), incubated on ice for 5min and centrifuged for 5min at 3700rpm to collect the cytoplasmic sample. The pellet was re-suspended in Nuclear Lysis Buffer (3mM EDTA, 0.2mM EGTA, 1mM DTT) and incubated on ice for 1 hr. Nuclear lysis was aided by sonication with a handheld homogeniser (VWR) for 10sec at 10min intervals. The nucleoplasmic supernatant and chromatin pellet were separated by centrifugation at 9000rpm for 10min at 4°C. The chromatin pellet was re-suspended in 160μl 2X Laemmli Buffer (Bio-Rad). Equal volumes of each fraction were used for WB. Cyto- and nucleoplasmic protein samples were diluted in 2X Laemmli Buffer and boiled for 5min, then loaded on a 4-20% SDS-PAGE gel (Bio-rad) or a 3-8% Tris Acetate gel (Invitrogen). Proteins were wet transferred onto a PDVF membrane (Millipore) and assessed with Ponceau Red (Sigma). The membrane was blocked with 10% milk and incubated with primary antibodies in 1% milk, 0.1% Tween-PBS overnight at 4°C. Membranes were imaged with SuperSignal West Femto Maximum Sensitivity (Thermo) on ImageQuant.

Table S2. Mass Spectrometry Analysis of STAG1 IP bands. Accession Q9D3E6

Band	Coverage	Peptide Sequence	Peptide AA position	PEP score
145 kDa	29.35%	HVESDVLEACSK	627-638	0.00005153
		ITDGSPSKEDLLVLR	752-766	0.00003838
140 kDa	27.34%	HDPQAEEALAKR	443-454	0.0002167
		ITDGSPSKEDLLVLR	752-766	0.0002349
130 kDa	19.27%	NMQNAEIIRK	141-150	0.0001146
		HTSTLAAMK	220-228	0.0005888
100 kDa	17.40%	HTSTLAAMK	220-228	0.01277
		HVESDVLEACSK	627-638	0.0003382
75 kDa	14.94%	HTSTLAAMK	220-228	0.001847
		LTSFHNAHDLTK	698-709	2.032E-15

Chromatin Co-Immunoprecipitation (co-IP) - Cells were re-suspended in 0.1% NP-40-PBS (1ml/1x10^7 cells) with 1X Protease Inhibitors (Roche) and 1mM DTT, and centrifuged at 1500rpm for 2min at 4°C. The pellet was re-suspended in Nuclear Lysis Buffer (3mM EDTA, 0.2mM EGTA, 1X Protease Inhibitors, 1mM DTT), vortexed for 30sec before being incubated on a rotator for 30min at 4°C and centrifuged at 6500g for 5min at 4°C to isolate the glassy chromatin pellet. This was re-suspended in High Salt Chromatin Solubilisation Buffer (50mM Tris-HCl pH 7.5, 1.5mM MgCl2, 300mM KCl, 20% glycerol, 1mM EDTA, 0.1% NP-40, 1mM Pefabloc, 1X Protease Inhibitors, 1mM DTT) with Benzonase (Sigma) (6U/1x10^7) and incubated on rotator for 30min at 4°C. Chromatin was digested with 3x 10sec sonication at 30% intensity with a Vibra-Cell probe. The supernatant was collected by centrifugation at 1300rpm for 30min at 4°C, and then diluted to 200mM KCl concentration. 30μl of Dynabeads (Invitrogen) were used per co-IP. Beads were washed 2x in 200mM KCl IP Buffer, re-suspended in IP Buffer with 10μg of the IP antibody, or an IgG-containing serum to match the species of the IP antibody and placed on rotator for 5h at 4°C. Beads were washed 3x in IP buffer and then incubated in 1mg chromatin lysate on a rotator overnight at 4°C. The beads were washed, re-suspended in 2X Laemmli Buffer (Bio-Rad) and boiled for 10min.

UMI-4C library preparation - 1x10^7 cells were fixed at RT for 10min in 1% formaldehyde and fixation was quenched with 0.125M Glycine for 5min. Cells were then lysed on ice in 10ml Lysis Buffer (10mM NaCl, 10mM Tris-HCl pH 8.0, 0.25% NP40, protease inhibitor) for 30min, followed by 10 strokes of douncing using a tight pestle. Nuclei were pelleted, 8min 700 rcf, washed in 1ml 1.2X DpnII buffer in Protein LoBind tubes (Eppendorf) and resuspended in 500 μl 1.2X DpnII buffer. 15ul of 10% SDS was added and incubated for 1hr at 37°C shaking at 650 rcf. 50ul of 20% TritonX was added to quench the SDS and incubated for 15 min at 37°C with shaking. 750U of DpnII was added and incubated overnight at 37**°C** with interval shaking. The next morning, nuclei were pelleted at 4°C by 650 rcf for 5 min and resuspended in 500μl 1X DpnII buffer. 500U DpnII was added and incubated for an additional four hours. The nuclei were washed twice in 100 μl of 1X T4 Ligase Buffer and resuspended in 200 μl Ligase Buffer**.** 6ul of T4 DNA Ligase was added and incubated for 3hr at 16°C**.** Nuclei were then pelleted, resuspended in 200 μl 1x fresh Ligase Buffer, 6μl of T4 DNA Ligase added, and incubated overnight at 16°C. Samples were treated with 20μl of ProtK (NEB Molecular Biology Grade), incubated for 3 hrs at 55°C and 5 hrs at 65°C to reverse crosslinks. Samples were treated with RNase A (PureLink, Invitrogen) for 1 hr at 37°C and DNA was extracted and precipitated

overnight. For library preparation, 3x5μg of ligated DNA was sonicated using Covaris (10% duty cycle, intensity 5, cycle burst 200, 70sec). Samples were end-repaired using DNA PolII Klenow Large Fragment (NEB), A-tailed using Klenow (exo-) (NEB), and Illumina indexed adapters ligated using Quick DNA Ligase (NEB). Reactions were denatured at 95°C for 3 min, placed on ice, and purified using 1.2X SizeSelect AmpPure beads to recover ssDNA. Libraries were amplified using GoTaq (Promega), with 20 cycles for PCR1 and 15 cycles for nested PCR2 on 50% material from 1st PCR. For custom UMI bait sequences, see Table S3.

Hi-C and UMI-4C-seq analysis

Hi-C libraries were analysed as previously described (Barrington et al., 2019). UMI-4C tracks were processed using the 'umi4cPackage' pipeline (v0.0.0.9000) (Schwartzman et al., 2016). Briefly, raw reads are parsed through the UMI-4C pipeline, those reads containing the bait and padding sequence are retained and de-multiplexed. Retained reads are split based on a match to the restriction enzyme sequence to create a segmented fastq file. The first 10 bases of read 2 are extracted and attached to the segments derived from each read pair. Mapping to mm10 is done with Bowtie2. Read pairs that have reverse complement segments are mapped to a restriction fragment ID, with the fragment ID, strand and distance from each end represented within a fragment-chain table. UMI filtering is used to determine the number of molecules supporting each ligation event. The resulting UMI-4C tracks are then imported into R, and data from multiple bait replicates can be merged by summing the molecule counts per ligated fragment, at which point contact intensity profiles and domainograms around the viewpoint can be generated. The contact intensity profile represents the mean number of ligations within a genomic window, with the resolution of the contact intensity profile being determined by the window size (set to 15 here). The domainogram reports the mean contact per fend at a series of window sizes, a stacked representation of contact intensity values in increasing window sizes from 10 to 300 fragment ends, their colour can be used to identify peak locations. ES and NSC contact profiles were compared after normalisation to correct for bias (see Schwartzman et al for further details). For the compared profiles, the total molecule count for restriction fragment ends for each are calculated at three ranges around the viewpoint. One profile is selected as reference and the second is scaled to the first using the ratio in total molecule counts between the two profiles. Below the contact profile is the profile resolution indicator, which shows the number of fends required to include at least 15 UMI molecules. The darker the colour, the larger the window size required. The domainogram at the bottom represents the log2 ratio between the domainogram values of the compared profiles and highlights locations where ESC has more contacts than NSC or vice versa.

REFERENCES

Barrington, C., et al. 2019. Enhancer accessibility and CTCF occupancy underlie asymmetric TAD architecture and cell type specific genome topology. Nature Communications 10, 2908. doi:10.1038/s41467-019-10725-9

Fidalgo, M., et al. 2016. Zfp281 Coordinates Opposing Functions of Tet1 and Tet2 in Pluripotent States. Cell Stem Cell 19, 355–369. doi:10.1016/j.stem.2016.05.025

Percharde, M., et al. 2018. A LINE1-Nucleolin Partnership Regulates Early Development and ESC Identity. Cell 174, 391–405.e19. doi:10.1016/j.cell.2018.05.043

Schwartzman, O., et al. 2016. UMI-4C for quantitative and targeted chromosomal contact profiling. Nat Meth 13, 685–691. doi:10.1038/nmeth.3922

Tapial, J., et al. 2017. An atlas of alternative splicing profiles and functional associations reveals new regulatory programs and genes that simultaneously express multiple major isoforms. Genome Res. 27, 1759–1768. doi:10.1101/gr.220962.117

Table S3. List of Primers used in this study, related to all Figures

PRIMER	Application	SEQUENCE
Actin B_fwd	qPCR	GGTGTTGAAGGTCTCAAACATG
Actin B_rev	qPCR	GAACATGGCATTGTTACCAACTG
Stag1_A_fwd	qPCR	GTACGGTCAGAATAGAGATGTTTCG
Stag1_A_rev	qPCR	GACACTGTCGAATCAGGACTCC
Stag1_C_fwd	qPCR	CCCTAATCTGGCTTTTCTAGAAGTAC
Stag1_C_rev	qPCR	CAGACATCCTGTCATCTTCACC
Stag2_fwd	qPCR	CCGAAATTCTTTGCTAGCTGG
Stag2_rev	qPCR	CCACATACTGTCACTGCTACTGC
Stag2_B_fwd	qPCR	CCGAAATTCTTTGCTAGCTGG
Stag2_B_rev	qPCR	CCACATACTGTCACTGCTACTGC
Smc3_fwd	qPCR	CAAGGATTTGGAGGATACCGAG
Smc3_rev	qPCR	CAACTCGAGCTTTGACTTCATTG
Smc1A_fwd	qPCR	CATGAGATGGAAGAGATTCGC
Smc1A_rev	qPCR	CCTTTGACAGTGGCAGTTTG
Nanog_fwd	qPCR	CGGACTGTGTTCTCTCAGGC
Nanog_rev	qPCR	CACCGCTTGCACTTCATC
Oct4_fwd	qPCR	CCCAAGGTGATCCTCTTCTGCTT
Oct4_rev	qPCR	GAGAAGGTGGAACCAACTCCCG
Dnmt3a_fwd	qPCR	GCTTCTCCGACTGTGGCC
Dnmt3a_rev	qPCR	CACCAAGACACAATTCGGC
Stag1_SATS_fwd	qPCR	GACACCTCTGTGACTAGTGAAGCC
Stag1_SATS_rev	qPCR	TGCTGGAGAAGCTATTCCACAG
L1spa-ORF1_fwd	qPCR	GAGAACACTGCTAAAGAGTTACAAGTCC
L1spa-ORF1_rev	qPCR	GGTCTAGTATGGTTTTGTTCATTTCC
MERVL-B_fwd	qPCR	TGGTGGTCGAGATGGAGGTTA
MERVL-B_rev	qPCR	CCGTGAATGGTGGTTTTAGCA
IAP_fwd	qPCR	GCACCCTCAAAGCCTATCTTAT
IAP_rev	qPCR	TCCCTTGGTCAGTCTGGATTT
Gm6763_fwd	qPCR	GCACCATACTGCAGACCAAAA
Gm6763_rev	qPCR	AGTGCACAGCAGATTTCTTCAAC
Dux_fwd	qPCR	AACTCCTCCTCCTTGATCAACTG
Dux_rev	qPCR	CTTCTCTCTGTGGCCAAAAGC
AW822073_fwd	qPCR	GTAGAAATTCTGGCAGCTGGG
AW822073_rev	qPCR	TTGATAGAGCAAGAGCTCCAGG

Gm4981_fwd	qPCR	GATAATAATGAAGTGCCTTCTGCAG
Gm4981_rev	qPCR	GTGAAGCCTAGTCCTAGTGTCCC
Stag1exon2-rev	5' RACE	TTGCTGGAGAAGCTATTCCACAGTACA
Stag1exon8-rev	5' RACE	CTCTTTACGTTTCTGAAGTAGTAACTCCAGTC
Stag1exon23-fwd	3' RACE	GCTGTTTGCCAACAGTGCCTATCTAATG
Stag1_SATS_5p-end	PCRminiscreen	ACTTCCGGTTCTAACTCCTTCCTC
Stag1_can-5p-end	PCRminiscreen	ATTGGCGTGTGGAAAATGC
Stag1_altex1-5p-end	PCRminiscreen	TGAAGGATGACAGCTACGCAC
Stag1_lastexon_rev	PCRminiscreen	TTCAGAACATAGGCATTCCAAATC
Stag1_intrn25_end_rev	PCRminiscreen	GTGCAGGGTGAGAGACATGG
Nanog_UMI4Cbait_nested	UMI-4C	AATGATACGGCGACCACCGAGATCTACACTCTTTCCCTACACGACGCTCTTCCGATCTCTCAGGTGCCAAAACCAACAG
Nanog_UMI4Cbait_PCR1	UMI-4C	GAAGCAGATGAGCACCAGACAC
CTCF_UMI4Cbait_nested	UMI-4C	AATGATACGGCGACCACCGAGATCTACACTCTTTCCCTACACGACGCTCTTCCGATCTACCTGGAGGCCAGCATAGAC
CTCF_UMI4Cbait_PCR1	UMI-4C	GGTGCTAACCTGGGCTTTG
Stag1canATG	Gateway cloning	GGGGAAGTTTGTACAAAAAAGCAGGC*TTCACC***ATGATTACTTCAGAGTTACCAGTGTTACAG**
Stag1canend	Gateway cloning	GGGGACCACTTTGTACAAGAAAGCTGGG**T***CGAACATAGGCATTCCAAATC*
intron25_end	Gateway cloning	GGGGACCACTTTGTACAAGAAAGCTGGGTCATTATCTATTTCTCTACCATACAGAAAGG
Stag1-exon5_exon6ATG_Kozak	Gateway cloning	GGGGAAGTTTGTACAAAAAAGCAGGCTTC*ACCATG*ATGTTTCGAAATATGCAGAACG
mSTAG1_e.33_FWD_2	CRISPR/Cas9 genotyping	ACTTCTTTGACTCTGCAGCTATCAT
mSTAG1_3' UTR_REV_2	CRISPR/Cas9 genotyping	AAACACACACACATCTGTACTGAGA
V5_FWD	CRISPR/Cas9 genotyping	CCTAACCCTCTCCTCGGTCT
Stag1_3'UTR_REV	CRISPR/Cas9 genotyping	CGGCGATTAGAACGAGCTGC
Stag1_3' gRNA_2	CRISPR/Cas9 gRNA	TTCTTCAGACTTCAGAACAT
