# Supplementary material for: The N-terminus of Stag1 is required to repress the 2C program by maintaining rRNA expression and nucleolar integrity
Source: Stem Cell Reports. 2023 Oct 5;18(11):2154–73. doi: 10.1016/j.stemcr.2023.09.004 (PMC10679541; doi:10.1016/j.stemcr.2023.09.004)
Supplement: Document S2. Article plus supplemental information [file mmc2.pdf]

Stem Cell Reports
Article
OPEN ACCESS

The N-terminus of *Stag1* is required to repress the 2C program by maintaining rRNA expression and nucleolar integrity

Dubravka Pezic,[1] Samuel Weeks,[1] Wazeer Varsally,[1] Pooran S. Dewari,[2] Steven Pollard,[2] Miguel R. Branco,[3] and Suzana Hadjur[1,*]
[1]Department of Cancer Biology, Cancer Institute, University College London, 72 Huntley Street, London, UK
[2]Centre for Regenerative Medicine, Institute for Regeneration and Repair, Cancer Research UK Scotland Centre, Edinburgh, UK
[3]Blizard Institute, Faculty of Medicine and Dentistry, QMUL, London, UK
*Correspondence: s.hadjur@ucl.ac.uk
https://doi.org/10.1016/j.stemcr.2023.09.004

SUMMARY

Our understanding of how STAG proteins contribute to cell identity and disease have largely been studied from the perspective of chromosome topology and protein-coding gene expression. Here, we show that STAG1 is the dominant paralog in mouse embryonic stem cells (mESCs) and is required for pluripotency. mESCs express a wide diversity of naturally occurring *Stag1* isoforms, resulting in complex regulation of both the levels of STAG paralogs and the proportion of their unique terminal ends. Skewing the balance of these isoforms impacts cell identity. We define a novel role for STAG1, in particular its N-terminus, in regulating repeat expression, nucleolar integrity, and repression of the two-cell (2C) state to maintain mESC identity. Our results move beyond protein-coding gene regulation via chromatin loops to new roles for STAG1 in nucleolar structure and function, and offer fresh perspectives on how STAG proteins, known to be cancer targets, contribute to cell identity and disease.

INTRODUCTION

Cohesin is a ubiquitously expressed, multi-subunit protein complex that has fundamental roles in cell biology including sister chromosome cohesion, chromatin topology, and regulation of cell identity (Cuartero et al., 2018; Horsfield et al., 2007; Kline et al., 2018; Leiserson et al., 2015; Romero-Pérez et al., 2019; Viny et al., 2019). Much of our understanding of how cohesin contributes to cell identity has been studied in the context of its roles in protein-coding gene expression and three-dimensional organization of interphase chromatin structure (Hadjur et al., 2009; Kagey et al., 2010; Mishiro and Tsutsumi, 2009; Misulovin et al., 2007; Parelho et al., 2008; Phillips-Cremins et al., 2013; Rao et al., 2014; Vietri Rudan et al., 2015; Wendt et al., 2008). Indeed, loss of cohesin and its regulators results in a dramatic loss of chromatin topology at the level of topologically associated domains (TAD) and chromatin loops, albeit with modest changes to gene expression (Haarhuis et al., 2017; Rao et al., 2017; Schwarzer et al., 2017; Seitan et al., 2013; Sofueva et al., 2013; Wutz et al., 2017; Zuin et al., 2014). This suggests that cohesin's roles in development and disease extend beyond gene expression regulation and highlight the need to re-evaluate how cohesin regulators shape the structure and function of the genome.

The association of cohesin with chromosomes is tightly controlled by several regulators, including the stromalin antigen protein (known as STAG or SA), which has been implicated in cell identity regulation and disease development (Cuadrado et al., 2019; Lehalle et al., 2017; Leiserson et al., 2015; Soardi et al., 2017; Viny et al., 2019; Yuan et al., 2019). STAG proteins interact with the RAD21 subunit of cohesin and mediate its association with DNA and CTCF (Hara et al., 2014; Li et al., 2020; Orgil et al., 2015; Xiao et al., 2011). Mammalian cells express multiple STAG paralogs, which have >90% sequence conservation in their central domain yet perform distinct functions (Canudas and Smith, 2009; Kojic et al., 2018; Remeseiro et al., 2012a; Winters et al., 2014). It is likely that the divergent N- and C-terminal regions provide functional specificity. For example, the N-terminus of STAG1 contains a unique AT hook (Bisht et al., 2013) that is required for its preferential participation in telomere cohesion (Canudas and Smith, 2009). Why cells have so many STAG proteins and the specific functions that each STAG protein performs to support a given cell state is poorly understood.

The nucleolus is a multifunctional nuclear compartment that coordinates ribosome biogenesis with cell-cycle control and mRNA processing (Boisvert et al., 2007). It forms through self-organization of its constituent proteins and the rDNA gene clusters into a tripartite, phase-separated condensate (Feric et al., 2016; Yao et al., 2019), which is intimately connected to overall nuclear organization (Padeken and Heun, 2014). In line with its liquid-like properties, the nucleolus is itself plastic, undergoing dramatic changes in response to cell cycle, metabolic, or developmental cues. For example, functional nucleoli play an important role in the control of cell identity during early mouse development (Kresoja-Rakic and Santoro, 2019). Two-cell (2C) stage totipotent embryos from mice exhibit "immature" nucleoli with poorly defined structure and

 © 2023 The Authors.
This is an open access article under the CC BY license (http://creativecommons.org/licenses/by/4.0/).

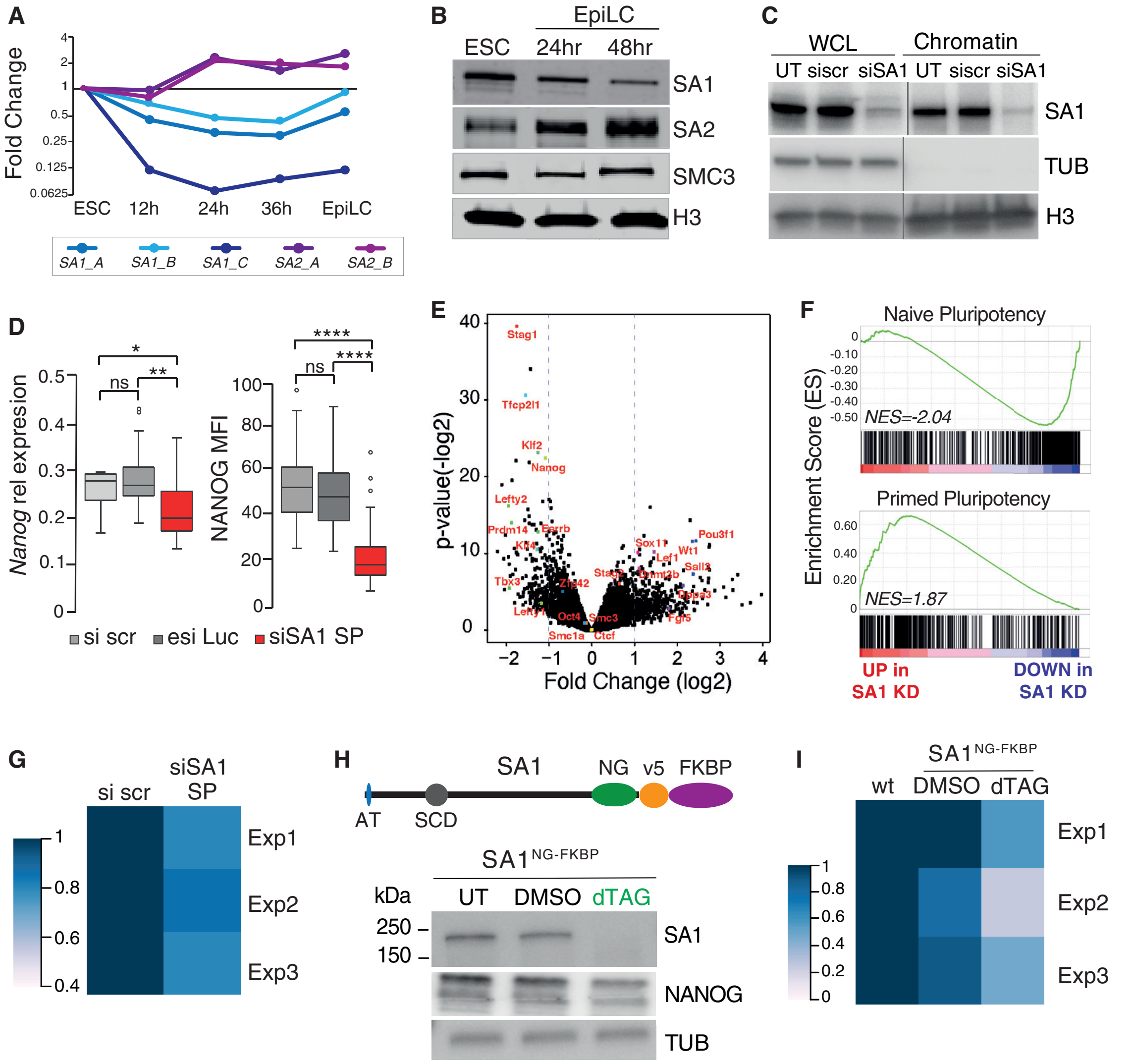

Figure 1. *Stag1* is required for naive pluripotency in mouse ESCs

(A) Log2 fold change of *Stag1* (*SA1*) and *Stag* (*SA2*) gene expression assessed by qRT-PCR during *in vitro* mESC differentiation toward EpiLCs. Multiple primer pairs were used for *Stag1* (blue) and *Stag2* (purple) mRNA (see box). Data are derived from two independent replicates.

(B) Whole-cell protein extracts (WCL) from naive mESCs and EpiLCs were analyzed by western blot (WB) for levels of SA1, SA2, and SMC3. H3 serves as a loading control.

(C) WB analysis of SA1 levels in WCL and chromatin fractions upon treatment with scrambled control siRNAs (si scr) or SmartPool SA1 siRNAs (siSA1) for 24 h in naive mESC cells. Tubulin (TUB) and H3 serve as fractionation and loading controls.

(D) Left: relative expression of *Nanog* mRNA by qRT-PCR in naive mESCs upon treatment with si scr, esiLuciferase control, or siSA1. Data are from eight independent replicates. Right, mean fluorescence intensity (MFI) of NANOG protein assessed by immunofluorescence (IF) in naive mESCs treated with same siRNAs as before. Cells were counterstained with DAPI. Data are n > 100 cells/condition across three independent replicates. Whiskers and boxes indicate all and 50% of values, respectively. The central line represents the median. Asterisks indicate a statistically significant difference as assessed using two-tailed t test. *$p < 0.05$, **$p < 0.005$, ***$p < 0.0005$, ****$p < 0.0001$; ns, not significant.

(E) Volcano plot displaying the statistical significance (−log2 p value) versus magnitude of change (log2 fold change) from RNA-seq data produced in mESCs treated with si scr or siSA1 for 24 h. Data are from three independent replicates. Vertical blue dashed lines represent changes of 2-fold. Selected genes associated with cohesin, pluripotency, and differentiation have been highlighted in red.

(F) Enrichment score (ES) plots from gene set enrichment analysis (GSEA) using curated naive or primed pluripotency gene sets (see experimental procedures). Negative and positive normalized enrichment scores (NES) point to the gene set being over-represented in the

(legend continued on next page)

low levels of perinucleolar heterochromatin (Aguirre-Lavin et al., 2012; Fulka et al., 2020). This global chromatin accessibility contributes to the expression of the 2C-specific transcription factor DUX and the subsequent activation of MERVL elements (Ishiuchi et al., 2015; Xie et al., 2022). As the mouse embryo reaches the eight-cell stage, cells harbor fully mature phase-separated nucleoli, defined heterochromatin around the nucleolar periphery (Németh et al., 2010), and robust rRNA expression, all of which are essential for cells to commit to differentiation (Gupta and Santoro, 2020; Kresoja-Rakic and Santoro, 2019). In contrast, mouse embryonic stem cells (mESCs) exhibiting nucleolar stress lead to conversion to 2C-like cell (2C-LC) identity *in vitro* (Grow et al., 2021) and nucleolar proteins that control rRNA transcription and processing are essential for 2C-LC repression (Sun et al., 2021), highlighting the tight relationship between rRNA levels, nucleolar structure, and cell identity.

It is known that cohesin is necessary for nucleolar integrity in yeast. Core cohesin subunits have been shown to bind to the non-transcribed region of the rDNA locus (Laloraya et al., 2000) and the 35S and 5S genes form loops that are dependent on Eco1, the cohesin subunit known to acetylate Smc3 and thus stabilize cohesin rings on chromatin (Harris et al., 2014). Consequently, yeast with Eco1 mutations exhibit disorganized nucleolar structure and defective ribosome biogenesis.

Here, we sought to understand how STAG proteins and their divergent ends influence cell identity. We reveal a novel role for STAG1, and in particular its unique N-terminal end, in regulating nucleolar integrity and 2C repression to maintain mESC cell identity. Our results offer fresh perspectives on how STAG proteins, known to be pan-cancer targets (Leiserson et al., 2015), contribute to cell identity and disease. STAG1 binds to repeats associated with nucleolar structure and function including rDNA and LINE-1 and interacts with the Nucleolin/TRIM28 complex that resides within perinucleolar chromatin to maintain nucleolar integrity. Loss of STAG1 or specifically the N-terminus in mESCs leads to reduced nascent rRNA and LINE-1, nucleolar disruption, increased expression of DUX, and conversion of mESCs to 2C-LCs. In addition to presenting a new role for STAG1 in repeat regulation, nucleolar structure, and translation control, our results also reveal a previously unappreciated transcriptional diversity of *Stag1* in stem cells and highlights the complexity of cohesin regulation in mammalian cells. We show that cells change both the levels of STAG paralogs as well as the proportion of their unique terminal ends to control cell identity and point to the importance of the divergent, unstructured ends of STAG1 proteins in nuclear body structure and cell fate control.

RESULTS

A functional change in cohesin regulation in cells of different potential

We analyzed the expression levels of cohesin regulators in mESCs by qRT-PCR in different pluripotent populations. During the transition between naive (2i mESCs) and primed epiblast-like pluripotent cells (EpiLCs) *in vitro*, levels of the core cohesin subunits *Smc1* and *Smc3* do not change, while *Stag1* becomes downregulated and *Stag2* becomes upregulated (Figures 1A, 1B, S1A, and S1B). This is supported by western blot (WB) analysis where we observe a 2- to 3-fold higher level of chromatin-associated STAG1 compared with STAG2 in naive (2i) mESCs, while STAG2 levels are 5- to 10-fold higher in EpiLCs (Figures 1B and S1C). These results, together with similar observations (Cuadrado et al., 2019), identify STAG1 as the dominant paralog in naive mESCs and suggest that a switch between *Stag1* and *Stag2* may represent a functionally important change in cohesin regulation at different stages of pluripotency.

STAG1 is required for pluripotency

To investigate the functional importance of *Stag1* in the regulation of pluripotency, we first established a *Stag1* knockdown (KD) ("siSA1," experimental procedures) strategy using siRNAs. This resulted in a significant reduction of *Stag1* at the mRNA and protein levels (4- to 5-fold and 8- to 10-fold, respectively), in both serum-grown (FCS) and naive mESCs without affecting the cell cycle

top-most down- or upregulated genes in *Stag1* KD mESCs, respectively. Vertical bars refer to individual genes in the gene set and their position reflects the contribution of each gene to the NES.

(G) Area occupied by AP^{hi} colonies relative to total colony area in mESCs treated with si scr and si SA1 from 3 independent replicates where n > 50 colonies/condition were counted.

(H) CRISPR-Cas9 was used to knock in a NeonGreen-v5-FKBP tag on both alleles of endogenous *Stag1* at the C-terminus ($SA1^{NG-FKBP}$). The resultant STAG1 protein is 42 kDa larger. Shown also are known features of STAG1 including the N-terminal AT-hook (AT) and the stromalin-conserved domain (SCD). WB analysis of STAG1 and NANOG levels in a targeted mESC clone after treatment with DMSO or dTAG. Tubulin (Tub) serves as a loading control.

(I) Area occupied by AP^{hi} colonies as above but in wild-type or $SA1^{NG-FKBP}$ mESCs treated with DMSO or dTAG. Data are from three independent replicates where n > 50 colonies/condition were counted. See also Figure S1.

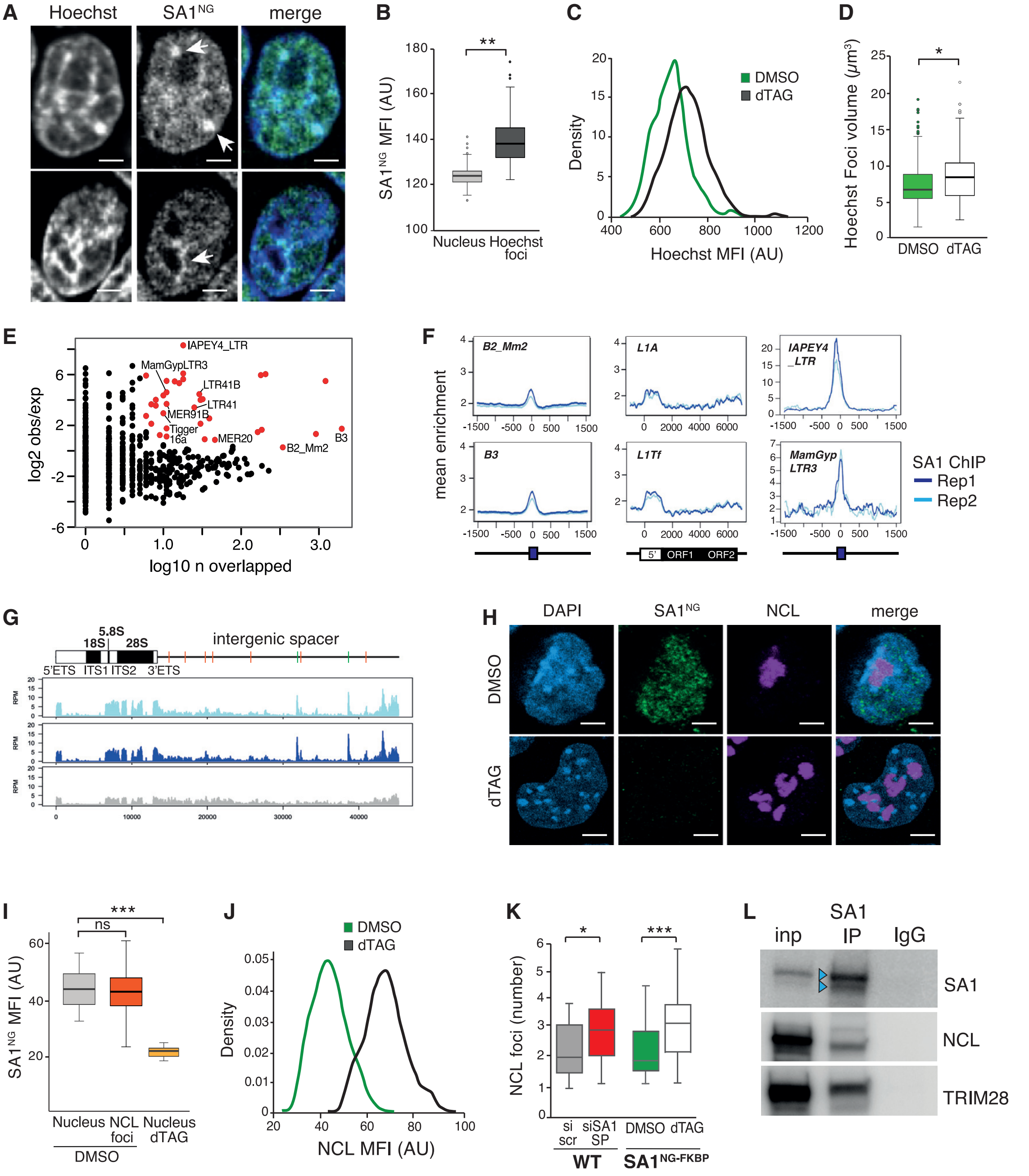

Figure 2. STAG1 is localized to and impacts both euchromatin and heterochromatin compartments

(A) Live-cell spinning disk confocal images of two SA1$^{NG-FKBP}$ mESCs counterstained with Hoechst. Arrows indicate notable regions of overlap of STAG1 and Hoechst, including at Hoechst-dense foci and at the nucleolar periphery. NB: puncta within the nucleoplasm can also be observed. Scale bars, 3 μm.

(B) Imaris quantification of the MFI of SA1-NeonGreen within the nucleus (light gray) or Hoechst-dense foci (dark gray). Quantifications and statistical analysis were done as above. Data are from n > 100 independent cells/condition in 3 replicates. AU, arbitrary units.

(C) Distribution of Hoechst MFI from SA1$^{NG-FKBP}$ mESCs treated with DMSO (green) or dTAG (black). Data are from n > 100 independent cells/condition in 2 replicates. AU, arbitrary units.

(D) Imaris quantification of the volume of Hoechst foci in SA1$^{NG-FKBP}$ mESCs treated with DMSO (green) or dTAG (white). Quantifications and statistical analysis were done as above. Data are from n > 100 independent cells/condition in 3 replicates. AU, arbitrary units.

(E) Number of copies of each repeat family that overlap an SA1 ChIP-seq peak and the enrichment of binding over random. Shown in red are the repeats that have significant enrichment, with a subset of these labeled.

(legend continued on next page)

(Figures 1C and S1D–S1F). Using Nanog as a marker of naive pluripotency, we observed a significant downregulation of *Nanog* mRNA and protein levels within 24 h of *Stag1* KD in mESCs (Figures 1D and S1G), suggesting that *Stag1* may be required for pluripotency. Global analysis of the mESC transcriptome using RNA sequencing (RNA-seq) upon siRNA-mediated *Stag1* KD revealed that 375 genes were up- and 205 genes were downregulated by at least 2-fold (Figure 1E). Among the downregulated group were genes known to have roles in the maintenance of pluripotency (i.e., *Nanog*, *Tbx3*, *Esrrb*, *Klf4*), while genes associated with exit from pluripotency (*Dnmt3b*, *Fgf5*) and differentiation (i.e., *Pou3f1*, *Sox11*) were upregulated (Figure 1E). Gene set enrichment analysis (GSEA) (Mootha et al., 2003; Subramanian et al., 2005) confirmed a reproducible loss of naive pluripotency-associated gene signature and enrichment for genes associated with primed pluripotency upon *Stag1* KD (Figures 1F and S1H).

The loss of the naive transcriptional program upon *Stag1* KD suggests that mESCs may require *Stag1* for the maintenance of self-renewal. To test this, we plated cells in self-renewal conditions at clonal density and determined the proportion of undifferentiated cells upon *Stag1* KD by measuring the area occupied by the colonies with high alkaline phosphatase activity (AP^{hi}). In scrambled siRNA-treated controls, 52% of plated cells retain their naive state, identified by AP^{hi} colonies, which was not significantly different from untreated cells. Upon *Stag1* KD, both the proportion of AP^{hi} colonies and the area they occupy decreased by an average of 20% compared with siRNA controls, indicating that mESCs have a reduced ability to self-renewal in the absence of *Stag1* (Figures 1G and S1I).

We validated these observations by using CRISPR-Cas9 to knock in an mNeonGreen-FKBP12^{F36V} tag (Nabet et al., 2018) at the C-terminus of both alleles of the endogenous *Stag1* locus (SA1^{NG_FKBP}) in mESCs (Figures 1H and S1J–S1L). Upon dTAG addition, STAG1 is robustly degraded in a SA1^{NG_FKBP} mESC clone (Figures 1H and S1L). As we had previously observed with siRNA treatment, dTAG-mediated degradation of STAG1 led to a reduction in NANOG protein (reduced by 24% compared with DMSO controls) (Figure 1H), and self-renewal potential was reduced by an average of 38% compared with DMSO-treated cells (Figure 1I). Together, our results are consistent with a requirement for STAG1 in the control of naive pluripotency.

STAG1 localizes to both euchromatin and heterochromatin

To understand how *Stag1* contributes to pluripotency, we first investigated its subcellular localization. Live-cell imaging of Hoechst-labeled SA1^{NG_FKBP} mESCs revealed the expected and predominant localization of STAG1 in the nucleus with a notable punctate pattern within the nucleoplasm (Figure 2A). STAG1 was also co-localized with Hoechst-dense regions (Figure 2A, arrows) and enriched in Hoechst-dense foci compared with the whole nucleus (Figure 2B). This was of interest since Hoechst stains AT-rich heterochromatin, which is enriched around the nucleolus, at the nuclear periphery and in discreet foci within the nucleoplasm (Padeken and Heun, 2014; Quinodoz et al., 2018). Acute degradation of STAG1 in SA1^{NG_FKBP} mESCs resulted in increased Hoechst signal intensity (Figure 2C) and a significant increase in Hoechst foci volume (Figure 2D). siRNA-mediated *Stag1* KD mESCs revealed similar changes to heterochromatin, as assessed by DAPI and H3K9me3 staining (Figures S2A and S2B).

These observations prompted us to re-analyze STAG1 chromatin immunoprecipitation (IP) followed by chromatin immunoprecipitation sequencing (ChIP-seq) data in mESCs (Cuadrado et al., 2019; Deniz et al., 2020). We calculated the proportion of STAG1 peaks that overlapped genes, repeats (within the Repeat Masker annotation), introns, and intergenic regions not already represented

(F) Profiles of the mean enrichment of STAG1 ChIP-seq at select TE repeat families. Shown are full-length elements of the indicated SINE, LINE, and LTR families. Two SA1 ChIP replicates are shown in blue.
(G) Top: cartoon of the consensus *Mus musculus* ribosomal DNA (rDNA) (GenBank: BK000964.3), showing the ribosomal genes and the intergenic spacer region which contains several SINE elements (red, B2_Mm2; green, B3). Bottom: Stag1 ChIP replicates and INPUT as in (F) above, aligned to this region.
(H) Representative confocal images of MFI of SA1-NeonGreen and Nucleolin (NCL) assessed by IF in SA1$^{NG-FKBP}$ mESCs treated with DMSO or dTAG and counterstained with DAPI. Scale bars, 3 μm.
(I) Imaris quantification of the MFI of SA1-NeonGreen from (H) within the nucleus or NCL foci in DMSO and dTAG conditions. Quantifications and statistical analysis were done as above. Data are from n > 100 independent cells/condition in 3 replicates. AU, arbitrary units.
(J) Distribution of NCL MFI from SA1$^{NG-FKBP}$ mESCs treated with DMSO (green) or dTAG (black). Data are from n > 100 independent cells/condition in 3 replicates. AU, arbitrary units.
(K) Imaris quantification of the number of NCL foci in wild-type mESCs treated with si scr (gray) or siSA1 SP siRNAs (red) and in the SA1$^{NG-FKBP}$ mESC clone treated with DMSO (green) or dTAG (white). Quantifications and statistical analysis were done as above. Data are from n > 100 independent cells/condition in 3 replicates. See also Figure S2.
(L) Chromatin immunoprecipitation of STAG1 and IgG from wild-type mESCs and WB for STAG1, NCL, and TRIM28. Blue arrows indicate multiple immunoreactive bands to STAG1. See also Figure S2.

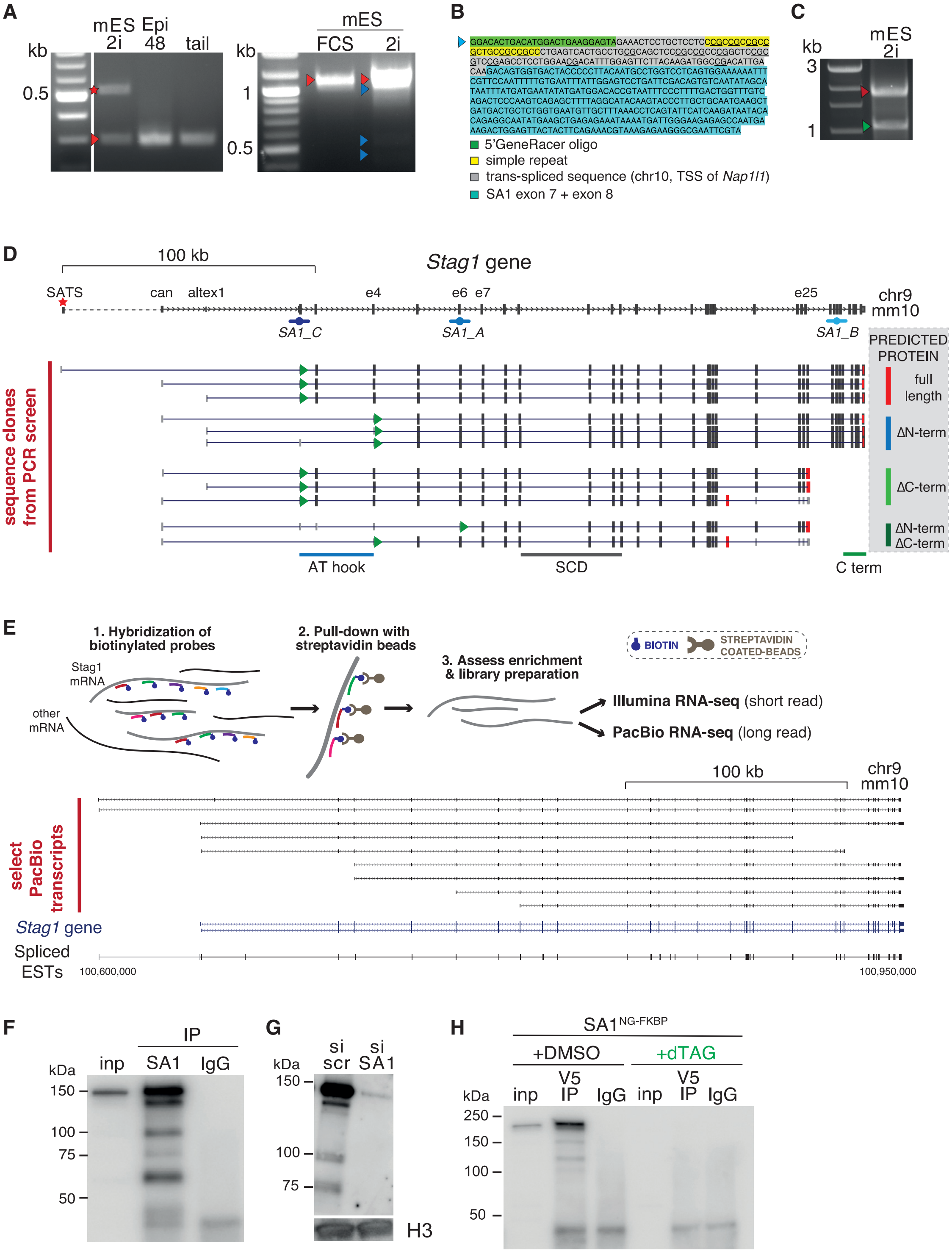

Figure 3. *Stag1* undergoes widespread transcriptional regulation in mESCs

(A) 5′ Rapid amplification of cDNA ends (RACE) for *Stag1* in naive mESCs and EpiLCs. Left gel: red star indicates SATS TSSs and red arrow indicates canonical (can) TSSs. Right gel: red arrow indicates full-length *Stag1* with both SATS and can TSSs; dark blue arrow indicates alternatively spliced variants arising from skipping of exons in the 5′ region; light blue arrows indicate the TSSs at exon 6 (e6) and exon 7 (e7). Arrows indicate bands that were cloned and sequenced.

(B) The 5′ RACE fragment that identified a new TSS at exon 7 spliced directly to a sequence in *trans* carrying regulatory elements.

(legend continued on next page)

(see experimental procedures). Of the 18,600 STAG1 peaks identified, the majority (76%) are bound to genomic sites that are distinct from protein-coding genes including at repetitive elements and intergenic regions (Figure S2C). Indeed, STAG1 binding was enriched at specific repeat families above random expectation (Figure 2E). These included the DNA transposon and retrotransposon classes, both known to form constitutive heterochromatin in differentiated cell types, and are expressed in early development and involved in regulation of cell fate (Hackett et al., 2017; Percharde et al., 2018). Specifically, STAG1 was enriched at SINE B3 and B2-Mm2 elements (previously shown to be enriched at TAD borders (Dixon et al., 2012); several LTR families, two of which have been previously shown to be associated with CTCF (LTR41, LTR55) (Schwalie et al., 2013) and at evolutionary young and active families of LINE1 elements (L1Tf, L1A) (Figures 2E, 2F, and S2E). We also found that several SINE B3 elements located within the intergenic spacer of the consensus rDNA locus were bound by STAG1 (Figure 2G). The binding of STAG1 at repeats may be dependent on CTCF since many of the bound repeats contained CTCF motifs (Figure S2D).

RNA-seq of siSA1-treated mESCs did not reveal dramatic changes in steady-state transcription of repetitive elements. However, qRT-PCR analysis using primers to ORF1 of STAG1-bound LINE1 and pre-rRNA revealed downregulation of rRNA and a modest but not significant effect on steady-state LINE transcripts (Figure S2F). Together with the microscopy results, the profile of STAG1 peaks suggests that the role of *Stag1* in mESCs may extend beyond protein-coding gene regulation.

STAG1 supports nucleolar structure in mESCs

Depletion of *Stag1* resulted in a loss of self-renewal and reduced rRNA expression. Furthermore, STAG1 was enriched at repetitive elements including LINE1 and rDNA. As it is known that mESC self-renewal and rRNA synthesis are promoted by a complex containing LINE1 RNA (Percharde et al., 2018), the nucleolar protein Nucleolin (NCL), and the co-repressor TRIM28 (Kap1) (Rowe et al., 2010), we considered whether STAG1 was supporting pluripotency through nucleolar structure and function. We used confocal imaging of SA1^{NG_FKBP} mESCs to assess the co-localization of STAG1 with nucleolar proteins. We observed a similar amount of SA1-NeonGreen (SA1^{NG}) within the nucleus compared with the nucleus of mESCs (Figures 2H and 2I). Notably, upon dTAG treatment of SA1^{NG_FKBP} mESCs, there was a significant increase in NCL signal intensity (Figure 2J) as well as increased numbers of nucleolar foci in both dTAG-treated SA1^{NG_FKBP} and in siSA1 KD mESCs (Figures 2K, S2G, and S2H), reminiscent of changes observed during mESC differentiation (Meshorer et al., 2006). Furthermore, STAG1 IP followed by WB in mESCs revealed an interaction with both NCL and Trim28 (Figure 2L), suggesting a direct effect of STAG1 on nucleolar structure and rRNA expression.

Stag1 expression is highly regulated in mESCs

We consistently observed several immunoreactive bands on STAG1 WB (Figure 2L, arrows), which were enriched in mESCs (Figure 1B). To gain a full perspective on how STAG1 may be contributing to nucleolar structure and pluripotency, we first investigated whether *Stag1* may be regulated at the level of transcription in mESCs. Several lines of evidence suggested that this may be the case. First, *Stag1* levels are higher in 2i-grown compared with FCS-grown mESCs (Figures S1B and S1C) and, second, primers positioned along the length of STAG1 amplify mRNAs that respond differently to differentiation (Figure 1A). Thus, we employed a series of approaches to comprehensively characterize *Stag1* mRNAs. First, we used RACE (rapid amplification of cDNA ends) to characterize the starts and ends of *Stag1* mRNAs directly from mESCs. 5′ RACE uncovered four novel alternative transcription start sites (TSSs) in mESCs; ~50 kb upstream of the canonical *Stag1* TSSs (referred to as "SATS," and previously identified in Feng et al., 2016) (Figures 3A, 3D, and S3A); between canonical exon 1 and exon 2 (referred to as alternative exon 1 or altex1) (Figures 3A, 3D, and S3D); and at exons 6 and 7 (Figures 3A, 3D, and S3A). Interestingly, the TSS located

(C) 3′ RACE for *Stag1* in naive mESCs. Red arrow indicates canonical full-length end; green arrow indicates end in i25. Arrows indicate bands that were cloned and sequenced.
(D) Top: schematic of the *Stag1* gene annotation in mm10. The identified TSSs and TTSs from RACE are indicated. Bottom: aligned sequence clones from the PCR mini-screen and their predicted impact on the STAG1 protein (gray box, right). Green arrows and red bars within the transcripts indicate start of the coding sequence and the TTS, respectively. Shown also are the regions that code for the AT hook and the stromalin-conserved domain (SCD).
(E) Schematic of the PacBio sequencing methodology (see experimental procedures for full description). Select transcripts sequenced on the PacBio platform, including many isoforms already discovered using RACE and PCR cloning methods above.
(F and G) WB analysis of endogenous, chromatin-bound STAG1 protein isoforms from (F) mESCs and (G) upon treatment with si scr and siSA1. H3 serves as a loading control.
(H) Chromatin immunoprecipitation for the v5 tag in SA1$^{NG-FKBP}$ mESCs treated with DMSO or dTAG to degrade STAG1. NB: STAG1 bands run 42 kDa higher due to the addition of the tag. See also Figures S3 and S4.

at exon 7 (e7) was preceded by a sequence located in *trans* to the *Stag1* gene, carrying simple repeats and transcription factor binding sites (Figure 3B). While the frequency of this alternative TSS was significantly lower than the other TSSs, it was identified in multiple RACE replicates, indicating that it may be present in a subset of the mESC population. We also discovered widespread alternative splicing in the 5′ region of *Stag1*, with particularly frequent skipping of exons 2 and 3 (e2/3Δ) and exon 5 (e5Δ) (Figures 3D, S3A, and S3F). Using 3′ RACE, we detected an early termination site in intron 25 and inclusion of an alternative exon 22 introducing an early STOP codon, as well as several 3′ UTRs (Figures 3C, 3D, and S3C).

Next, PCR- and Sanger sequencing-based clonal screening confirmed that the newly discovered 5′ and 3′ ends represent true *Stag1* transcript ends, validated the existence of the e2/3Δ and e5Δ isoforms, confirmed their enrichment in naive mESCs compared with differentiated mouse embryonic fibroblasts (MEFs), and uncovered an isoform lacking exon 31, which encodes a basic domain embedded in the otherwise acidic C-terminal region of *Stag1* (e31Δ) (Figure 3D). To determine the complete sequences of the *Stag1* transcript isoforms and to use a non-PCR-based approach, we performed long-read PacBio Iso-seq from 2i mESC RNA (Figure 3E). This confirmed the diversity of the *Stag1* 5′ and 3′ UTRs, the e31Δ isoform, multiple TSSs including SATS, and early termination events, including in i22 and i25 (Figures 3E and S3E). Importantly, these transcripts all had poly(A) tails, in support of their protein-coding potential. Finally, we validated and quantified the newly discovered splicing events by calculating the frequency (percentage spliced in) of exon splicing in our RNA-seq as well as in published data using the VAST-tools method (Tapial et al., 2017). This confirmed the presence of *Stag1* splicing events in other mESC datasets and supported that several of these were specifically enriched in mESCs (Figure S3F; Table S1).

Interestingly, visual inspection of the genome topology around the *Stag1* locus in our 2i mESC and neural stem cell (NSC) Hi-C data (Barrington et al., 2019) revealed that the *Stag1* gene undergoes significant 3D reorganization as cells differentiate (Figure S4). For example, the *Stag1* TAD switches from the active to the repressive compartment during differentiation, in line with the decrease in *Stag1* levels during differentiation. Furthermore, UMI-4C revealed changes to sub-TAD architecture corresponding to the newly discovered mESC-enriched *Stag1* TSSs and TTSs described above, suggesting that 3D chromatin topology may play a role in facilitating the transcriptional diversity of *Stag1* (Figure S4). Together, our results point to a previously unappreciated diversity of endogenous *Stag1* transcripts in mESCs, prompting us to investigate the importance of these for pluripotency and the nucleolus.

Multiple STAG1 protein isoforms are expressed in mESCs

Stag1 transcript diversity was intriguing because many of the events were either specific to mESCs or enriched compared with MEFs and NSCs (Figures S3D and S3F). Furthermore, the transcript variants were predicted to produce STAG1 protein isoforms with distinct structural features and molecular weights (Figures 3D and S3G). For example, the truncation of the N-terminus (e2/3Δ, e5Δ, e6 TSS, and e7 TSS), and thus loss of the AT hook (amino acids 3–58) could impact STAG1 association with nucleic acids. Meanwhile, C-terminal truncated STAG1 isoforms (altex22, i25 end, e31Δ) could affect STAG1-cohesin interactions. It is noteworthy that the evolutionarily conserved stromalin domain (amino acids 296–381) (Orgil et al., 2015), shown to play a role in CTCF interaction (Li et al., 2020), would be retained in the isoforms identified here.

IP of endogenous STAG1 followed by WB revealed multiple bands corresponding to the predicted molecular weights for several protein isoforms and identified by mass spectrometry to contain STAG1 peptides (Figure 3F; Table S2). Similarly, multiple bands of expected sizes were reduced between naive and primed cells (Figure S3H) and sensitive to *Stag1* KD, alongside the canonical, full-length isoform (Figure 3G). Treatment of SA1^{NG_FKBP} mESCs with dTAG followed by WB of chromatin-associated proteins with an antibody to the v5 tag further confirmed the sensitivity of the isoforms to dTAG-mediated degradation (Figure 3H). Thus, complex transcriptional regulation in mESCs gives rise to multiple *Stag1* transcripts and protein isoforms with distinct regulatory regions and coding potential. Our discovery of such naturally occurring isoforms offers a unique opportunity to define the functions of the divergent N- and C-terminal ends of STAG1 in the context of the pluripotent state.

Experimentally modulating the levels of the N- and C-terminal ends of *Stag1*

To study the functional consequences of the *Stag1* isoforms on pluripotency and nucleolar structure, we took advantage of our detailed understanding of *Stag1* transcript diversity to design custom siRNAs to selectively target or retain specific isoforms (Figure 4A). Alongside the siRNAs used in Figure 1 (SmartPool [SP]), we designed siRNAs to specifically target the SATS 5′ UTR (esiSATS), the 5′ end (siSA1-5p), or the 3′ end (siSA1-3p) of Stag1 mRNA (see experimental procedures). We anticipated that the KD panels would reduce Stag1 levels and change the relative proportions of the N- and C-terminal ends of STAG1 in cells. 3p siRNAs were predicted to downregulate full-length and

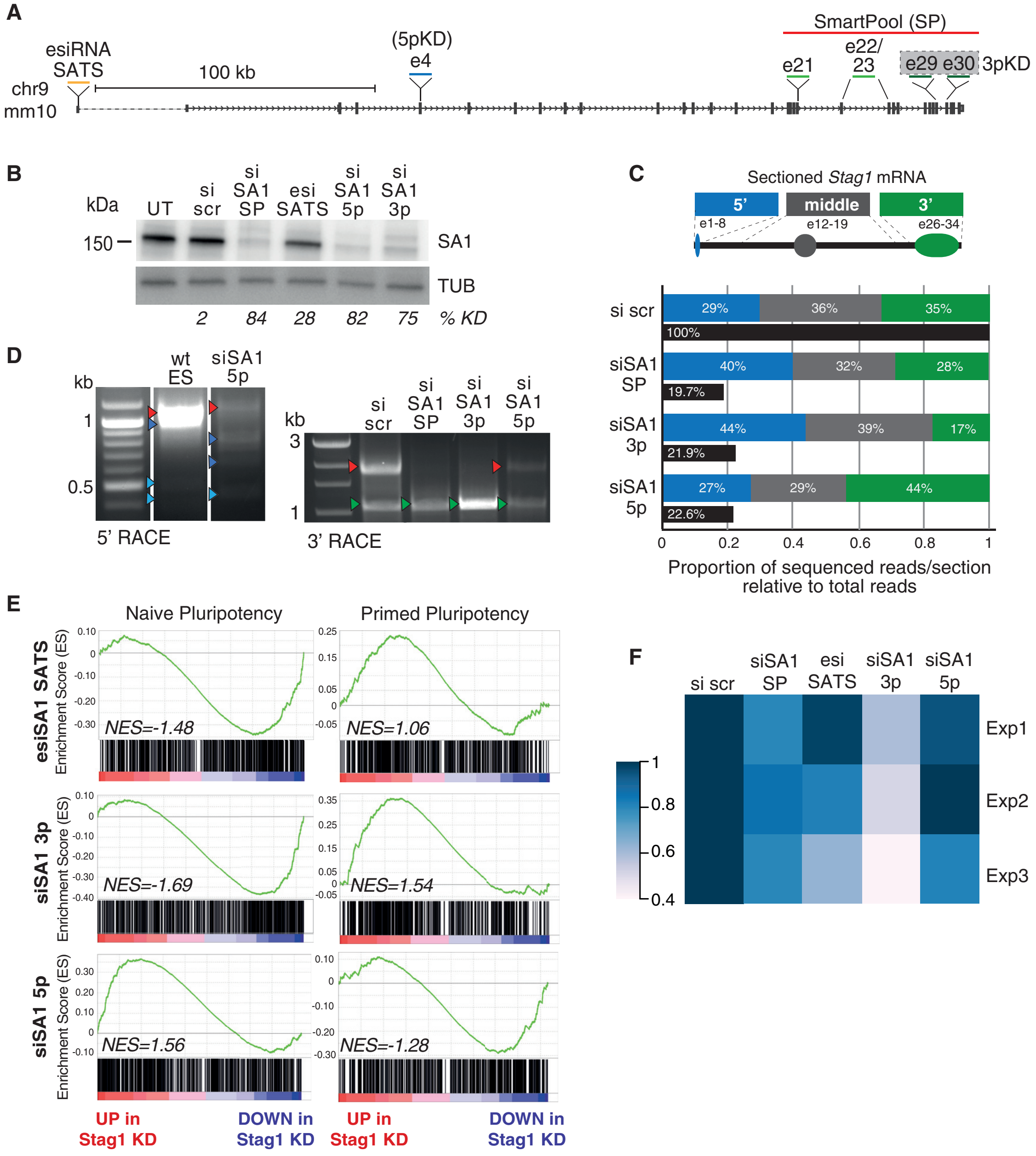

Figure 4. Fluctuations in the levels of the *Stag1* isoforms skews cell fates

(A) Schematic of the siRNA pools used in this study. esiRNA SATS represents “enzymatically prepared” siRNAs (see experimental procedures).

(B) Representative WB analysis of STAG1 levels in mESC WCL after no treatment (UT), or upon si scr, si SA1 SP, si SA1 3p, si SA1 5p, or esi SATS treatment. Tubulin (TUB) serves as a loading control. The percentage of knockdown (KD) of STAG1 signal normalized to tubulin from this specific experiment is shown.

(C) The proportion of RNA-seq reads that align to three sections of *Stag1* mRNA relative to the total number of reads. Shown are the exons within each section that the reads correspond to. RNA-seq from mESCs treated with scr, SP, 5p, and 3p siRNAs was used. Solid black bars represent *Stag1* transcripts per million in the different KDs to highlight the similar degree of KD in all conditions. NB: the change in read proportions from residual *Stag1* in the different KD treatments.

(D) Left gel 5′ and right gel 3′ RACE for *Stag1* in mESCs treated with the indicated siRNAs. Arrows indicate bands that were cloned and sequenced and color coded as before.

(legend continued on next page)

N-terminal truncated isoforms and retain C-terminal truncated isoforms, while 5p siRNAs would specifically retain N-terminal truncated isoforms.

We confirmed that *Stag1* isoform proportions were altered upon siRNA treatment using qRT-PCR, RNA-seq, RACE, and IP. siRNAs to the 5p and 3p ends of *Stag1* reduce full-length *Stag1* mRNA and protein with similar efficiency to SP KDs. esiSATS reduces *Stag1* by ~30%–50%, indicating that the SATS TSS functions to enhance expression of *Stag1* in naive mESCs (Figures 4B, S5A, and S5B). RNA-seq reads aligning to *Stag1* in the different siRNA treatments were quantified to represent the residual N-terminal (exons 1–8), middle (exons 12–19), and C-terminal (exons 26–34) read proportions. Residual reads in the SP and 3p KDs aligned predominantly to the N-terminal and were relatively depleted from the C-terminal. While the 5p KD had the opposite effect with the least read retention in the N-terminal and relatively more reads aligning to the C-terminal (Figures 4C and S5C). In parallel, we performed RACE to validate changes to the proportions of *Stag1* isoforms. 5′ RACE performed in mESCs treated with 5p siRNA revealed downregulation of full-length *Stag1* transcript while several N-terminal truncated isoforms were upregulated compared with untreated cells (Figure 4D, left panel, blue arrows). Similarly, 3′ RACE captures the canonical 3′ end of *Stag1* (Figure 4D, right panel, red arrows), which is strongly reduced in the SP and 3p siRNA KD samples and to a lesser extent in the 5p KD, further support that the residual transcripts in the 5p KD have C-terminal ends. Meanwhile, the transcript terminating in i25 is substantially enriched upon 3p KD (Figure 4D, green arrows) compared with all other conditions. Thus, the siRNA panel developed here provide us with a powerful tool to modulate the proportion of the naturally occurring *Stag1* isoforms in mESCs and study their potential roles in pluripotency.

A specific role for the STAG1 C-terminus in the maintenance of naive pluripotency transcriptome

We first quantified the effect of the *Stag1* siRNA KDs on pluripotency gene expression. qRT-PCR for *Nanog* expression and WB for NANOG protein levels revealed that the 3p KD had a similar effect on *Nanog* to SP, with significant downregulation, while, surprisingly, the 5p KD did not reduce *Nanog* (Figure S5D). We prepared independent replicate RNA-seq libraries from the *Stag1* 3p, 5p, and SATS siRNA KDs. We used GSEA as before to probe for signatures of naive or primed pluripotency. In support of our previous results, reducing *Stag1* levels by targeting the mESC-specific SATS promoter leads to downregulation of the naive pluripotency gene signature and upregulation of the primed signature (Figures 4E and S5E), reminiscent of the phenotype from SP KD (Figures 1E and 1F). We again observed a differential effect of the 3p and 5p KDs on naive and primed pluripotency signatures. A similar but more prominent loss of the naive signature was observed in 3p KD RNA-seq compared with SATS and SP while, surprisingly, in 5p KD cells the naive signature was unaffected compared with si scr controls (Figure 4E).

The distinct gene expression profiles of the 3p and 5p KDs were reflected in differences in self-renewal. Cells treated with 3p siRNAs exhibited a significant loss of self-renewal potential, consistent with the loss of the naive pluripotency signature, with only 20% of colonies exhibiting AP staining compared with 30% of colonies in the SP KDs (Figure S1I), and an average reduction of the area occupied by AP+ colonies of 50% compared with si scr controls (Figure 4F). This was not evident in the 5p KD, where the effect on self-renewal was more similar to si scr controls (Figure 4F). Interestingly, unlike siRNA to Stag1, esiSATS results in a variable effect on self-renewal (ranging from between 5% and 35% reduction in AP+ area) (Figure 4F), likely because the SATS TSS is expressed in the most naive cells of the population, the frequency of which varies significantly between FCS populations. Our results further confirm the importance of Stag1 in self-renewal and point to a specific role for the C-terminal of Stag1 in maintaining a naive pluripotency gene expression program.

The N-terminus of STAG1 supports nucleolar structure and function

The different effect on naive pluripotency between the 3p and 5p KDs was surprising. We therefore sought to reexamine the effect of our siRNA panel on the STAG1 bound repeats LINE1 and rDNA (Figures 2F and 2G). As we had not observed a significant difference on steady-state levels of repeats from our RNA-seq experiments, we instead purified nascent RNA from mESCs treated with siRNAs. Both the KD and the nascent RNA pull-downs were successful as revealed by qRT-PCR to *Stag1* (Figures 5A and 5B). Consistent with our previous results, total *Nanog* RNA levels were significantly reduced in siSA1 SP and 3p KD but not in 5p KD. Interestingly, this trend was not observed in nascent levels of Nanog RNA where the 3p KD does not have a significant effect, suggesting that the C-terminus may be required for the stability of *Nanog* mRNA instead of its transcription per se (Figures 5A and 5B). Upon *Stag1* SP KD,

(E) Enrichment score (ES) plots from GSEA using the naive and primed gene sets as in Figure 1E and RNA-seq data from the indicated siRNA-treated mESC samples.
(F) Area occupied by AP^{hi} colonies relative to total colony area in mESCs treated with the siRNA panel from three independent replicates. n > 50 colonies/condition were counted.

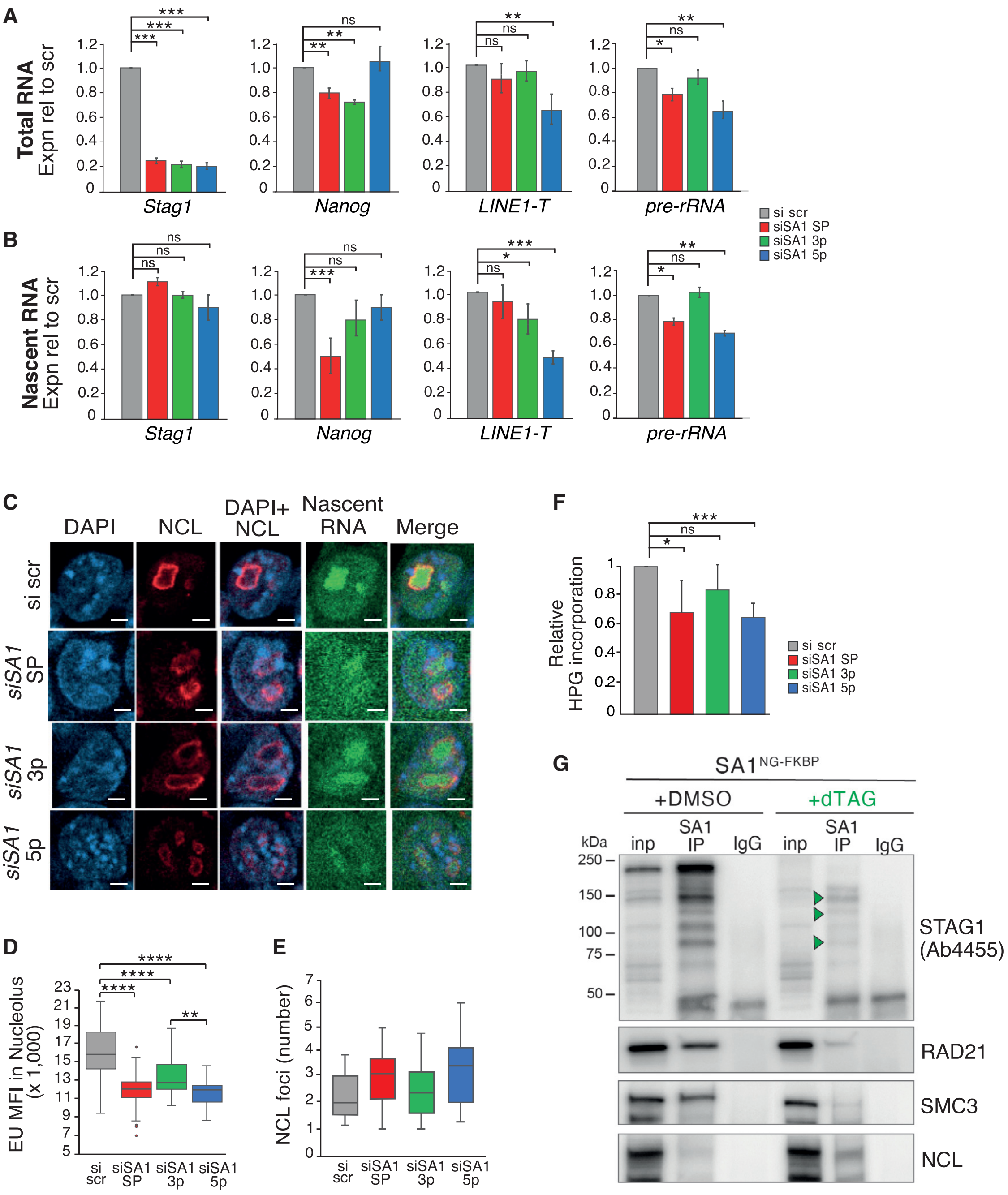

Figure 5. The N- and C-terminal ends of STAG1 regulate expression in different genomic compartments

(A and B) Relative expression of *Stag1*, *Nanog*, *LINE1-T*, and *pre-rRNA* by qRT-PCR in mESCs after treatment with the siRNA panel. Shown are (A) total and (B) nascent RNA levels. Data are represented as mean ± SEM and statistical analysis as before. Data are from three independent experiments.

(C) Representative confocal images of IF to NCL and nascent RNA in siRNA-treated mESCs labeled with EU-488. Nuclei were counterstained with DAPI. Scale bars, 2 μm.

(D) Imaris quantification of the MFI of nascent RNA (EU) within the nucleoli from (C), as defined by a mask made to the NCL IF signal. Quantifications and statistical analysis were done as above. Data are from four independent replicates. n > 50/condition, except for siSA1 5p where n > 35.

(E) Imaris quantification of the number of NCL foci in siRNA-treated mESCs. Quantifications and statistical analysis were done as above. Data are from n > 100 independent cells/condition in 2 independent replicates. See also Figure 2K.

(legend continued on next page)

both steady-state and nascent levels of LINE1 RNA were modestly decreased (Figures 5A, 5B, and S2F). While the 3p KD had a 20% reduction in LINE1 RNA expression, this was not maintained at steady-state levels. However, both nascent and total levels of LINE1 RNA were significantly reduced by 40%–50% of controls in 5p KD mESCs. These results were also observed for pre-rRNA, with only the SP and 5p KD having significant effects on expression. Thus, the N-terminus of *Stag1* plays a distinct role in LINE1 and rDNA expression.

Given the effects on LINE1 and rRNA, we also assessed nucleolar structure and function using our siRNA panel. mESCs were pulsed with 5-ethynyl uridine (EU), which becomes incorporated into nascent RNA and enables detection of newly synthesized RNA. Samples for immunofluorescence (IF) were co-stained with an antibody to NCL to simultaneously quantify nucleoli number and changes in nascent RNA transcription. Cells treated with scrambled siRNA showed a distinct nucleolar structure and the EU signal could be seen throughout the nucleus, with a strong enrichment within the nucleolus as expected from rRNA expression (Figure 5C). While a significant reduction in nascent RNA signal was observed in all KD conditions compared with scrambled controls (Figure S5F), by IF we observed a distinct effect on nascent RNA levels within the nucleolus in the 5p KD. The medians between the three siSA1 KDs were not dramatically different; however, the effect of the 5p KD on nucleolar RNA signal distribution was significantly different from the 3p KD (Figure 5D). This result was consistent with the qRT-PCR analysis of nascent pre-rRNA levels (Figure 5B) and with the significant effect on NCL foci number in 5p KD mESCs (Figure 5E). Consequently, we also observed changes to global translation by assessing the incorporation of L-homopropargylglycine (HPG), an amino acid analog of methionine into mESC using FACS analysis. HPG incorporation was significantly reduced in SP and 5p siRNA-treated mESCs compared with scrambled control (32% and 35% of si Scr) (Figures 5F and S5G). We did observe a modest effect on global nascent translation in 3p KD-treated cells (16% of si scr), although this was not significantly different from scrambled control. Our results reveal distinct roles for the N- and C termini of Stag1 in nucleolar structure and function and pluripotency gene expression, respectively.

The effects observed on rRNA levels and nucleolar function were not associated with changes to expression of ribosome subunit expression (Figure S5H). Thus, we considered whether the regulation of LINE1 expression by the N-terminus of STAG1 influenced nucleolar structure via the NCL/Trim28 complex (Figure 2L). To investigate this, we took advantage of our SA1^{NG_FKBP} mESCs. dTAG treatment can only degrade isoforms containing the FKBP tag inserted into the canonical C-terminal end. Thus SA1^{NG_FKBP} mESCs treated with dTAG should enrich for SA1$^{\Delta C}$ isoforms that contain an N-terminus. Indeed, IP of STAG1 using an antibody that recognizes an N-terminal epitope reveals the presence of several N-terminal-enriched SA1ΔC isoforms (Figure 5G, green arrows). WB of this IP material revealed a reduction in the ability of SA1ΔC to interact with the cohesin subunits RAD21 and SMC3, despite similar levels in the input of dTAG-treated cells. Meanwhile, the interaction with NCL was increased in the same lysate (Figure 5G). Taken together, our results are supportive of the different ends of STAG1 interacting with different protein partners to coordinately regulate pluripotency.

The N-terminus of STAG1 suppresses the 2C-LC state

In addition to promoting rRNA synthesis and self-renewal in mESCs, the LINE-1/NCL/Trim28 complex represses transcriptional program-specific 2C-LCs (Percharde et al., 2018). The phenotypes of the 5p KD, namely reduced rRNA and LINE-1 expression, reduced translation and aberrant nucleolar function, pointed toward possible conversion of cells into a 2C-LC state. We therefore tested whether STAG1, and specifically the N-terminal end, play a role herein.

We first investigated whether 2C-LCs, which naturally arise within mESC populations, express *Stag1* NΔ isoforms. To formally address this, we obtained mESCs expressing a Dox-inducible *Dux-HA*-expression construct together with a MERVL-linked GFP reporter (Hendrickson et al., 2017). Dux is a 2C-specific transcription factor that binds to MERVL elements to activate expression (Hendrickson et al., 2017). We induced *DuxHA* expression in the MERVL-GFP mESCs and performed 5′ RACE as before on sorted GFP+ (2C-L) and GFP– cells (Figure 6A). We enriched several of the previously identified N-terminal truncated *Stag1* transcripts in the GFP+ population including e2/3Δ and e5Δ isoforms (Figure 6A, blue arrows). Importantly, we also identified a transcript starting at e7, similar to the one previously found in 5p KD mESCs (Figures 6B, 3A,

(F) Analysis of global levels of nascent translation by measuring HPG incorporation using flow cytometry and analyzed using FloJo software. Shown is the quantification of the change in EU incorporation relative to si scr-treated cells. Data are from four independent replicates.
(G) Chromatin immunoprecipitation using an N-terminal Stag1 antibody (Ab4455) in SA1$^{NG-FKBP}$ mESCs treated with DMSO or dTAG. Green arrow indicates residual C-terminal truncated STAG1 isoforms. Shown also are WB for the core cohesin subunits RAD21 and SMC3 and NCL. See also Figure S5.

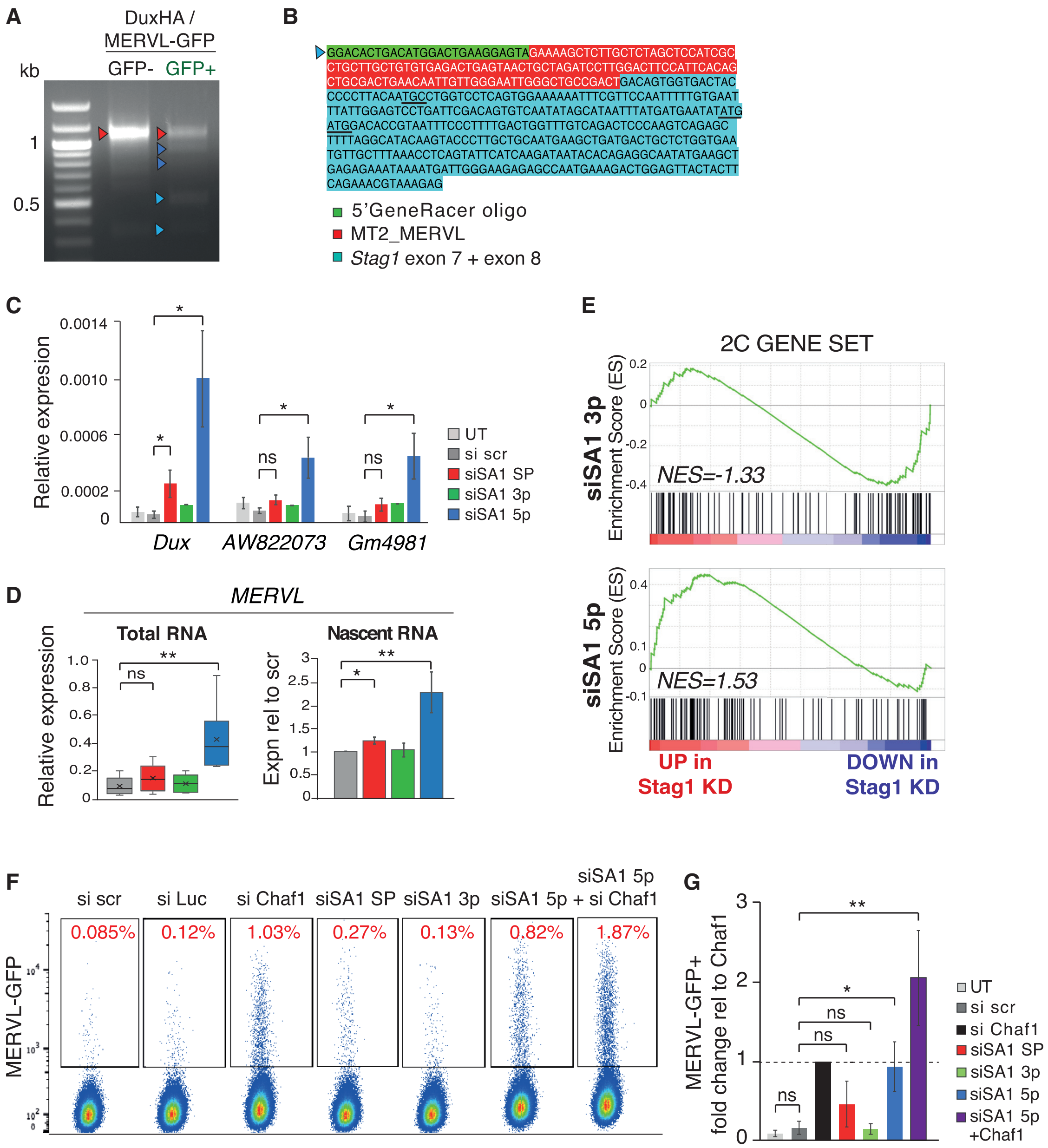

Figure 6. STAG1 N-terminus protects against conversion of ESCs to 2C-LCs

(A) 5′ RACE for *Stag1* in Dux-HA MERVL-GFP mESCs with and without sorting for GFP+ cells. Arrows indicate bands that were cloned and sequenced and color coded as described previously.

(B) Sequence of the 5′ RACE product identifying a novel *Stag1* TSS from (A) with direct splicing of exon7 to an MT2_MERVL element.

(C) Relative expression of several 2C-LC markers in total RNA by qRT-PCR in mESCs after treatment with the siRNA panel. Data are represented as mean ± SEM and statistical analysis as before. Data are from six independent experiments.

(D) Relative expression of MERVL repeat element by qRT-PCR in mESCs after treatment with the siRNA panel. Shown are total (left) and nascent RNA (right) levels. Quantifications and statistical analysis as before. Data are from five independent replicates. NB: nascent RNA levels are shown relative to si scr control.

(E) Enrichment score (ES) plots from GSEA using a published 2C-L gene set and RNA-seq data from the 3p and 5p siRNA-treated mESC samples used in Figure 4.

(legend continued on next page)

and 3B). Remarkably, however, the sequence preceding the TSS in e7 in *Dux*-induced cells was an MT2-MERVL element, creating a chimeric, LTR-driven *Stag1* transcript, reminiscent of other LTR transcripts specifically expressed in the 2C-L state.

2C-LCs are a rare subpopulation that spontaneously arise in mESC cultures and exhibit unique molecular and transcriptional features (Eckersley-Maslin et al., 2016; Ishiuchi et al., 2015; Macfarlan et al., 2012). Given that 2C-LCs expressed several N-terminal truncated *Stag1* isoforms, we investigated whether these in turn supported the maintenance or emergence of that state. We treated mESCs with the panel of siRNAs and used qRT-PCR to test expression of candidate genes. We found that *Dux*, and consequently MERVL and other markers of the 2C-LC state, *Gm6763*, *AW822073*, and *Gm4981*, are strongly upregulated by 5p KD (Figures 6C, 6D, and S6A). Notably, all 2C-L genes analyzed remained unchanged in 3p KD conditions with a modest upregulation in SP KD. Furthermore, GSEA using a published 2C gene set (Percharde et al., 2018) revealed a specific enrichment among the upregulated genes in 5p KDs that was not observed in 3p KDs (Figures 6E and S6B), consistent with the different ends of *Stag1* targeting different RNA pools.

To functionally validate the expression results, we returned to the Dox-inducible *Dux-HA*, MERVL-GFP mESCs (Hendrickson et al., 2017) and used flow cytometry to directly measure the number of GFP+ cells in our different *Stag1* KD conditions (Figures 6F and 6G). Chaf1 is a chromatin accessibility factor previously shown to support conversion of mESCs toward totipotency (Ishiuchi et al., 2015). In support of the upregulation of the 2C-LC gene set in 5p KD mESCs, we observed an 8- to 9-fold increase in the proportion of GFP+ cells in 5p KD conditions compared with scramble-treated controls, similar to the published effect of Chaf1 KD (Figures 6F and 6G). There was a modest, but insignificant increase in GFP+ cells upon SP KD and no effect upon 3p KD. mESCs treated with both Chaf1 and 5p siRNAs had an additive effect on the proportion of GFP+ cells, suggesting that the two proteins function in complementary pathways for conversion toward 2C-LCs. Thus, 2C-LCs express N-terminal truncated *Stag1* isoforms, which in turn supports the maintenance or emergence of that state through rRNA repression and nucleolar changes. Together our results reveal a new and specific role for the N-terminus of STAG1 in the regulation of the 2C-LC state.

DISCUSSION

Most studies of cohesin function focus on the core trimer, despite the fact that it is the regulatory STAG subunit that is a pan-cancer target (Leiserson et al., 2015) and has clear roles in cell identity control (Viny et al., 2019). How these proteins contribute to cohesin's functions, why cells have diversified them so extensively, and how their mutations lead so often to disease are poorly understood. Here, we reveal a novel role for *Stag1*, and in particular its unique N-terminal end, in regulating nucleolar integrity and 2C repression to maintain mESC identity. It has been known for a long time that several STAG paralogs exist in mammalian cells and that they have non-reciprocal functions with respect to chromosome structure and cohesion. By dissecting the diversity of naturally occurring *Stag1* isoforms in mESCs, we have shed new light not only on the unique divergent ends of the STAG paralogs but also the critical role that their levels play in cell fate control. Our results highlight the importance of careful understanding of chromatin regulators in cell-specific contexts.

Stag1 knockout ($Stag1^{\Delta/\Delta}$) ESCs give rise to mice that survive to E13.5 (Remeseiro et al., 2012a, 2012b). At first this observation seems at odds with our report that *Stag1* is required for pluripotency. However, our observations may in fact explain why the $Stag1^{\Delta/\Delta}$ mouse model does not exhibit early embryonic lethality. In this model, only the 5′ region of *Stag1* was targeted, meaning that the *Stag1* isoforms lacking the N-terminus may still be retained in the targeted ESCs. This is consistent with our results showing that 5p KD cells have not lost their ability to self-renew nor is their pluripotency gene signature affected. It further suggests that changes to the nucleolus may exist in these cells. We acknowledge that, while the N-terminus is important for regulation of totipotency *in vitro*, it is possible that it may be dispensable *in vivo* during early development and future work in mice could resolve this.

The nucleolus is held together by liquid-liquid phase separation (PS), which is driven by the association of rDNA with nucleolar proteins and is dependent on continual rRNA synthesis (Feric et al., 2016; Yao et al., 2019). However, in one- to two-cell embryos, nucleoli lack distinct compartments, and exhibit low rRNA synthesis and low translation (Borsos and Torres-Padilla, 2016). Similarly, changes to rRNA synthesis or nucleolar PS are sufficient to convert ESCs toward the 2C-LC state, either through Dux dissociation from the

(F) Representative FACS analysis of the proportion of mESCs expressing a MERVL-GFP reporter in the different siRNA-treated cells and including siRNA to *Chaf1* as a positive control. Percentage of MERVL-GFP+ cells based on Flo-Jo analysis is shown in red.
(G) Proportion of MERVL-GFP+ cells in the different siRNA conditions relative to the siChaf1 positive control. Data are represented as mean ± SEM and statistical analysis as before and is from four independent experiments.

nucleolar periphery and consequently its de-repression (Xie et al., 2022) or p53-mediated nucleolar stress (Grow et al., 2021). Other proteins including the NCL/TRIM28 complex (Percharde et al., 2018) and nucleolar LIN28 (Sun et al., 2021) have been shown to contribute to nucleolar integrity and repress DUX expression. In this context, our results position STAG1, and specifically its N-terminal end, as a novel regulator of the 2C-ESC transition through the control of nucleolar integrity. STAG1 is localized to the nucleolar periphery and interacts with the nucleolar proteins NCL/TRIM28 as well as being bound to and supporting rDNA and LINE-1 element expression. Our results suggest that the N-terminus of STAG1 plays a specific role in repressing conversion to the 2C state. STAG1 may contribute to nucleolar structure and function via both the regulation of rRNA expression as well as by supporting nucleolar PS through interactions with nucleolar regulators. In this context, modulating the availability of the N- or C-terminus of STAG1 may be a way in which ESCs impact nucleolar structure and function and thus cell identity. Our results also point to the different ends of STAG1 interacting with different protein partners since mESCs retaining the C-terminus of STAG1 do not exhibit changes to the nucleolus and do not convert into 2C-LCs. This is also supported by the different gene expression programs affected in the KDs that select for N-terminalΔ or C-terminalΔ isoforms. It may in fact be quite important for ESCs to express a diversity of alternative *Stag1* isoforms to support the plasticity of nucleolar structure and a range of cell fate options from totipotency to primed pluripotency.

Finally, *Stags* are commonly mutated in cancers (Leiserson et al., 2015). Our results point to misregulation of STAG proteins as leading to changes in epigenetic regulation that move beyond changes to TADs and protein-coding genes. Instead, they support a role for hierarchical changes to chromatin organization, nucleolar structure and function, and repeat deregulation in cell fate determination. Careful analysis of *Stag2*-mutant cancers should shed light on these and deliver new insights into cancers that harbor these mutations.

EXPERIMENTAL PROCEDURES

Resource availability

Corresponding author

Further information and requests for resources and reagents should be directed to and will be fulfilled by the corresponding author, Suzana Hadjur (s.hadjur@ucl.ac.uk).

Materials availability

Mouse ESC lines generated in this study are available from the lead contact with a completed Materials Transfer Agreement.

Data and code availability

Genomic data generated in this study are available at GEO with the Accession GSE160390.

ESC culture, siRNA KD, and qRT-PCR

Male E14 mESCs were cultured in serum (FCS) or naive (2i) conditions. Serum-cultured cells were grown on 0.1% gelatin-coated plates in GMEM, 10% FCS (Sigma), NEAA, Na pyruvate, 0.1 mM β-mercaptoethanol (BMe), GlutaMAX, and freshly added LIF (1:10,000). 2i-cultured cells were grown on plates coated with fibronectin, in DMEM:F12/neurobasal 1:1, KnockOut Serum Replacement, N2, B27, GlutaMAX, 1 μM PD0325901, 3 μM CHIR9902, 0.1 mM BMe, and freshly added LIF as above. DuxHA/MERVL-GFP cells were cultured in 2i conditions. siRNAs were purchased from Horizon Discovery (previously Dharmacon) or Sigma (for "enzymatically derived" esiRNAs). siRNA KDs were performed for 24 or 72 h (for Figure 6) in 6-well plates where 200,000 cells were seeded for 72 h KDs, and 400,000 for 24 h KDs. siRNAs (50 pmol) were transfected using RNAiMax Lipofectamine at the time of seeding and, after 48 h for 72 h time points. Two siRNA controls were used, scrambled (scr) was D-001810-10 and Luciferase (esiLuc) Sigma. siSA1 SP was derived from equimolar ratios of commercial siRNAs (D-041989-02, -04, -05, -06, -07, or -08). siSA1 5p was a custom Duplex siRNA sequence (AGGAGCAGGUCGUGGAAGAUU). siSA1 3p was derived from equimolar ratios of commercial siRNAs J-041989-05, -07, or -08. esiRNA to SATS was a custom-made product (Sigma) to the entire SATS 5′ UTR (mm10 chr9:100,597,794–100,598,109). Total RNA was isolated using a Monarch RNA prep kit (NEB). Reverse transcription was performed on 0.5 μg DNase-treated total RNA using Lunascript RT (NEB) in 20-μL reactions. qPCR was performed using 2× SensiFAST SYBR No-ROX kit (Bioline) in 20 μL reactions using 1 μL of RT reaction as input and 0.4 μM of each primer.

AP assay and quantification

Cells were seeded in 6-well plates and transfected with siRNAs as above. After 24 h, cells were collected for RNA isolation and KD efficiency analyzed by qRT-PCR. Cells from each condition were counted and 1,000 cells per well seeded into a new 6-well plate. Cells were re-transfected after 48 h using 5 pmol of siRNAs. Four days after seeding cells at clonal density, the cells were assayed for AP expression using a StemTAG Alkaline Phosphatase staining kit (Cell Biolabs CBA-300). AP-stained cells were imaged in 6-well plates using an M7000 Imaging System (Zeiss) with a 4× objective and a trans-illumination bright-field light source. For quantification, the area occupied from all colonies/conditions was assessed (using the area tool in ImageJ) and then, separately, the area of the dark purple/AP^{hi} colonies was assessed. To normalize for the number of colonies, the AP^{hi} area was expressed as a fraction of the total colony area (percent of total). The percentages are shown relative to 1 in the main figures for the heatmaps for each biological replicate separately.

RACE and PCR mini screen

RACE was performed using GeneRacer kit (RLM RACE, Invitrogen L1500). Two micrograms of total RNA was used as input. Final products were amplified by nested PCR, using Kapa 2× MasterMix. First, PCR was carried out in a 50 μL reaction using 1 μL RT as input, 25 cycles. DNA was purified using a QIAGEN PCR Purification kit, and nested PCR was performed on a 10th of the first PCR for 30 cycles. The viewpoint for 5′ RACE was in exon 2 (Figure 3A) or exon 8 (Figure 3B) of Stag1. The viewpoint

for 3′ RACE was in exon 23 (Figure 3C). RACE primer details can be found in Table S3. PCR products were excised from the gel, A-tailed using Klenow exo- (NEB) and cloned into pCR4-TOPO vector (Invitrogen). At least three clones were sequenced per PCR product. For the PCR Mini-Screen, forward primers at either SATS or canonical 5′ UTR were used with reverse primers either at the end of Stag1 canonical coding sequence, or at the end of coding sequence in intron 25 (see Table S3). PCR was performed using Kapa 2× MasterMix. DNA was excised from the gel, A-tailed, and cloned into pCR4-TOPO. At least six clones per PCR product were Sanger sequenced. Sequences from the PCR Mini-screen were aligned using Minimap2 (2.14-r884) in "splice" mode to ensure long read splice alignment (Figures 3D and S3A).

Protein analysis including WBs, coIP, and IF

Please see supplemental experimental procedures where these are described in detail.

Nascent transcription and translation analysis

For nascent transcription analysis, we used the Click-iT RNA Alexa Fluor 488 HCS Assay (Invitrogen C10327). Cells were labeled with 1 mM EU for 45 min at 37°C in fresh medium. Cells were fixed in solution or onto coverslips with 3.7% paraformaldehyde and permeabilized with 0.5% Triton X-solution. Cells were incubated with the Click-iT reaction cocktail for 30 min and then either processed further for IF (directly to the blocking step) or analyzed by flow cytometry on a BD Fortessa X20. For the nascent translation analysis, a Click-iT HPG Alexa Fluor 594 Protein Synthesis Assay Kit (Invitrogen C10429) was used. Cells were pre-incubated at 37°C in methionine-free medium for 30 min before addition of HPG at 50 μM. Cells were incubated with HPG for 30 min, then collected, fixed, permeabilized, and stained using Click-It reaction in low retention tubes. HPG incorporation was measured by flow cytometry. FACS analysis was done with FloJo software (v.10.7.1).

RNA-seq library preparation and sequencing

ESCs were treated for 24 h with siRNA pools to *Stag1* and two sets of control siRNAs, scrambled (SCR), and Luciferase (Luc). There are three replicate sets for SP KD and two for the siRNA pools (SATS, SP, 3p, and 5p). Total RNA was isolated using an NEB Monarch RNA prep kit. One microgram of total RNA was rRNA-depleted using an NEBNext rRNA depletion kit (human/mouse/rat). Libraries were prepared from 10 to 50 ng rRNA-depleted total RNA using an NEBNext Ultra II directional RNAseq kit according to the manufacturer's instructions using eight cycles of PCR. All ESC FCS libraries were rRNA depleted and the ESC 2i libraries were poly(A) enriched (with two rounds of enrichment). RNA-seq libraries were sequenced on the Illumina HiSeq3000 platform, with 75 bp paired-end or single-end reads. Reads were quality controlled using FASTQC. RNA-seq data were processed using the RNA-seq Nextflow pipeline (v.19.01.0), with the following parameters: –aligner hisat2 –genome mm10, with –reverse_stranded specified for paired-end samples. FeatureCounts output was parsed through edgeR (v.3.16.5) and DESeq2 (v.1.14.1) to generate normalized expression counts. The normalized counts for RNA-seq (Figure 1) were calculated in edgeR. Low-expressed genes were removed (rowSum cpm <2 across SCR and SA1SP replicates), normalization factors were calculated using calcNormFactors, and dispersions estimated using estimateDisp. The edgeR volcano plot statistics were calculated using the exactTest and topTags functions. To generate the normalized counts for RNA-seq experiments required to calculate the log2FC GSEA ranked lists, the FeatureCounts output for all experiments was combined into a single table and read into DESeq2. A DESeq2 object was built using the function DESeqDataSetFromMatrix and estimation of size factors and dispersions were calculated using the DEseq function. Normalized counts were calculated using the "counts" function. Low-expressed genes (rowSum normalized count <10 across all samples) were removed. See supplemental experimental procedures for detailed information about GSEA and VAST-tools.

PacBio library, sequencing, and analysis

ESCs were cultured in naive 2i conditions, and poly(A)-enriched mRNAs were hybridized to a custom biotinylated oligonucleotide probe set. Post-capture, mRNAs were amplified using the Clontech SMARTer PCR cDNA Synthesis Kit with nine cycles and used in the SMRTbell library prep according to the manufacturer's instructions. The library was sequenced on the SMRTseq 2000 platform. PacBio reads were processed through the SMRTLINK v.8.0.0 IsoSeq3 pipeline. A total of 403,995 circular consensus sequences (CCSs) were generated using default parameters (–minPasses = 1, –min-rq = 0.8, CCS Polish = No). Further refining through lima (removal of adapters and correct orientation of sequences), poly(A) trimming, and concatamer removal resulted in 265,106 full-length non-chimeric (FLNC) reads. FLNC reads were aligned to the mm10 genome using Minimap2 with the following parameters (-ax splice, -uf, -k14).

ChIP-seq analysis

Previously published STAG1 ChIP-seq datasets from ES 2i cells (GSE126659) were trimmed using trim_galore and aligned to mm10 using bowtie2. Peak detection was performed with MACS2 using unique reads (MAPQ $\geq$ 2). Peaks were overlapped with genomic features in a hierarchical manner (promoters > exons > repeats > introns > intergenic), and overlap frequency was compared with a randomly shuffled version of the peaks. To identify repeat families enriched for STAG1 peaks, a previously described pipeline was used (Deniz et al., 2020) that compares family-levels overlap frequency with that observed in 1,000 permutations of random peak shuffling. Coverage profiles across specific TE families were generated using HOMER and including multi-mapping reads (MAPQ < 2).

UMI-4C and Hi-C

Please see the supplemental experimental procedures where these are described in detail.

SUPPLEMENTAL INFORMATION

Supplemental information can be found online at https://doi.org/10.1016/j.stemcr.2023.09.004.

ACKNOWLEDGMENTS

This work was supported by the Wellcome Trust through a Senior Research Fellowship awarded to S.H. (106985/Z/15/Z). We would

like to thank Sally Lowell and Mattias Malaguti for advice throughout the project. We are grateful to the members of the Hadjur lab for critical discussions and reading of the manuscript. We thank M. Irima for help with VAST-tools pipelines, B. Cairns for the inducible Dux-HA, MERVL-GFP ESCs, W. Reik for a second MERVL-reporter ESC line, and H. Rowe for advice on 2C-LC cells and repeat analysis. Thank you to the Earlham Institute for PacBio library preparation and sequencing and to Y. Guo and J. Manji in the Cancer Institute CRUK Center FACS and Imaging core facilities for their invaluable assistance.

AUTHOR CONTRIBUTIONS

D.P. and S.H. conceived the project. D.P. designed and performed all the experiments with assistance from S.W. S.W. performed protein analysis, generated the SA1-NG-FKBP mESC line by CRISPR, spinning disk microscopy, and helped with the siRNA KD experiments. W.V. performed all bioinformatic analyses with the exception of the STAG1 enrichments at repeat elements and TE analysis, which was done by M.R.B. P.S.D. and S.P. provided advice on CRISPR targeting. D.P. and S.H. formatted all figures and wrote the manuscript with input from all authors.

DECLARATION OF INTERESTS

The authors declare no competing interests.

Received: January 30, 2023
Revised: September 7, 2023
Accepted: September 8, 2023
Published: October 5, 2023

REFERENCES

Aguirre-Lavin, T., Adenot, P., Bonnet-Garnier, A., Lehmann, G., Fleurot, R., Boulesteix, C., Debey, P., and Beaujean, N. (2012). 3D-FISH analysis of embryonic nuclei in mouse highlights several abrupt changes of nuclear organization during preimplantation development. BMC Dev. Biol. *12*, 30. https://doi.org/10.1186/1471-213X-12-30.

Barrington, C., Georgopoulou, D., Pezic, D., Varsally, W., Herrero, J., and Hadjur, S. (2019). Enhancer accessibility and CTCF occupancy underlie asymmetric TAD architecture and cell type specific genome topology. Nat. Commun. *10*, 2908. https://doi.org/10.1038/s41467-019-10725-9.

Bisht, K.K., Daniloski, Z., and Smith, S. (2013). SA1 binds directly to DNA through its unique AT-hook to promote sister chromatid cohesion at telomeres. J. Cell Sci. *126*, 3493–3503. https://doi.org/10.1242/jcs.130872.

Boisvert, F.-M., van Koningsbruggen, S., Navascués, J., and Lamond, A.I. (2007). The multifunctional nucleolus. Nat. Rev. Mol. Cell Biol. *8*, 574–585. https://doi.org/10.1038/nrm2184.

Borsos, M., and Torres-Padilla, M.-E. (2016). Building up the nucleus: nuclear organization in the establishment of totipotency and pluripotency during mammalian development. Genes Dev. *30*, 611–621. https://doi.org/10.1101/gad.273805.115.

Canudas, S., and Smith, S. (2009). Differential regulation of telomere and centromere cohesion by the Scc3 homologues SA1 and SA2, respectively, in human cells. J. Cell Biol. *187*, 165–173. https://doi.org/10.1083/jcb.200903096.

Cuadrado, A., Giménez-Llorente, D., Kojic, A., Rodríguez-Corsino, M., Cuartero, Y., Martín-Serrano, G., Gómez-López, G., Marti-Renom, M.A., and Losada, A. (2019). Specific Contributions of Cohesin-SA1 and Cohesin-SA2 to TADs and Polycomb Domains in Embryonic Stem Cells. Cell Rep. *27*, 3500–3510.e4. https://doi.org/10.1016/j.celrep.2019.05.078.

Cuartero, S., Weiss, F.D., Dharmalingam, G., Guo, Y., Ing-Simmons, E., Masella, S., Robles-Rebollo, I., Xiao, X., Wang, Y.-F., Barozzi, I., et al. (2018). Control of inducible gene expression links cohesin to hematopoietic progenitor self-renewal and differentiation. Nat. Immunol. *19*, 932–941. https://doi.org/10.1038/s41590-018-0184-1.

Deniz, Ö., Ahmed, M., Todd, C.D., Rio-Machin, A., Dawson, M.A., and Branco, M.R. (2020). Endogenous retroviruses are a source of enhancers with oncogenic potential in acute myeloid leukaemia. Nat. Commun. *11*, 3506–3514. https://doi.org/10.1038/s41467-020-17206.

Dixon, J.R., Selvaraj, S., Yue, F., Kim, A., Li, Y., Shen, Y., Hu, M., Liu, J.S., and Ren, B. (2012). Topological domains in mammalian genomes identified by analysis of chromatin interactions. Nature *485*, 376–380. https://doi.org/10.1038/nature11082.

Eckersley-Maslin, M.A., Svensson, V., Krueger, C., Stubbs, T.M., Giehr, P., Krueger, F., Miragaia, R.J., Kyriakopoulos, C., Berrens, R.V., Milagre, I., et al. (2016). MERVL/Zscan4 Network Activation Results in Transient Genome-wide DNA Demethylation of mESCs. Cell Rep. *17*, 179–192. https://doi.org/10.1016/j.celrep.2016.08.087.

Feng, G., Tong, M., Xia, B., Luo, G.Z., Wang, M., Xie, D., Wan, H., Zhang, Y., Zhou, Q., and Wang, X.J. (2016). Ubiquitously expressed genes participate in cell-specific functions via alternative promoter usage. EMBO Rep. *17*, 1304–1313. https://doi.org/10.15252/embr.201541476.

Feric, M., Vaidya, N., Harmon, T.S., Mitrea, D.M., Zhu, L., Richardson, T.M., Kriwacki, R.W., Pappu, R.V., and Brangwynne, C.P. (2016). Coexisting Liquid Phases Underlie Nucleolar Subcompartments. Cell *165*, 1686–1697. https://doi.org/10.1016/j.cell.2016.04.047.

Fulka, H., Rychtarova, J., and Loi, P. (2020). The nucleolus-like and precursor bodies of mammalian oocytes and embryos and their possible role in post-fertilization centromere remodelling. Biochem. Soc. Trans. *48*, 581–593. https://doi.org/10.1042/BST20190847.

Grow, E.J., Weaver, B.D., Smith, C.M., Guo, J., Stein, P., Shadle, S.C., Hendrickson, P.G., Johnson, N.E., Butterfield, R.J., Menafra, R., et al. (2021). p53 convergently activates Dux/DUX4 in embryonic stem cells and in facioscapulohumeral muscular dystrophy cell models. Nat. Genet. *53*, 1207–1220. https://doi.org/10.1038/s41588-021-00893-0.

Gupta, S., and Santoro, R. (2020). Regulation and Roles of the Nucleolus in Embryonic Stem Cells: From Ribosome Biogenesis to Genome Organization. Stem Cell Rep. *15*, 1206–1219. https://doi.org/10.1016/j.stemcr.2020.08.012.

Haarhuis, J.H.I., van der Weide, R.H., Blomen, V.A., Yáñez-Cuna, J.O., Amendola, M., van Ruiten, M.S., Krijger, P.H.L., Teunissen, H., Medema, R.H., van Steensel, B., et al. (2017). The Cohesin Release Factor WAPL Restricts Chromatin Loop Extension. Cell *169*, 693–707.e14. https://doi.org/10.1016/j.cell.2017.04.013.

Hackett, J.A., Kobayashi, T., Dietmann, S., and Surani, M.A. (2017). Activation of Lineage Regulators and Transposable Elements across a Pluripotent Spectrum. Stem Cell Rep. *8*, 1645–1658. https://doi.org/10.1016/j.stemcr.2017.05.014.

Hadjur, S., Williams, L.M., Ryan, N.K., Cobb, B.S., Sexton, T., Fraser, P., Fisher, A.G., and Merkenschlager, M. (2009). Cohesins form chromosomal cis-interactions at the developmentally regulated IFNG locus. Nature *460*, 410–413. https://doi.org/10.1038/nature08079.

Hara, K., Zheng, G., Qu, Q., Liu, H., Ouyang, Z., Chen, Z., Tomchick, D.R., and Yu, H. (2014). Structure of cohesin subcomplex pinpoints direct shugoshin-Wapl antagonism in centromeric cohesion. Nat. Struct. Mol. Biol. *21*, 864–870. https://doi.org/10.1038/nsmb.2880.

Harris, B., Bose, T., Lee, K.K., Wang, F., Lu, S., Ross, R.T., Zhang, Y., French, S.L., Beyer, A.L., Slaughter, B.D., et al. (2014). Cohesion promotes nucleolar structure and function. Mol. Biol. Cell *25*, 337–346. https://doi.org/10.1091/mbc.E13-07-0377.

Hendrickson, P.G., Doráis, J.A., Grow, E.J., Whiddon, J.L., Lim, J.-W., Wike, C.L., Weaver, B.D., Pflueger, C., Emery, B.R., Wilcox, A.L., et al. (2017). Conserved roles of mouse DUX and human DUX4 in activating cleavage-stage genes and MERVL/HERVL retrotransposons. Nat. Genet. *49*, 925–934. https://doi.org/10.1038/ng.3844.

Horsfield, J.A., Anagnostou, S.H., Hu, J.K.-H., Cho, K.H.Y., Geisler, R., Lieschke, G., Crosier, K.E., and Crosier, P.S. (2007). Cohesin-dependent regulation of Runx genes. Development *134*, 2639–2649. https://doi.org/10.1242/dev.002485.

Ishiuchi, T., Enriquez-Gasca, R., Mizutani, E., Bošković, A., Ziegler-Birling, C., Rodriguez-Terrones, D., Wakayama, T., Vaquerizas, J.M., and Torres-Padilla, M.-E. (2015). Early embryonic-like cells are induced by downregulating replication-dependent chromatin assembly. Nat. Struct. Mol. Biol. *22*, 662–671. https://doi.org/10.1038/nsmb.3066.

Kagey, M.H., Newman, J.J., Bilodeau, S., Zhan, Y., Orlando, D.A., van Berkum, N.L., Ebmeier, C.C., Goossens, J., Rahl, P.B., Levine, S.S., et al. (2010). Mediator and cohesin connect gene expression and chromatin architecture. Nature *467*, 430–435. https://doi.org/10.1038/nature09380.

Kline, A.D., Moss, J.F., Selicorni, A., Bisgaard, A.-M., Deardorff, M.A., Gillett, P.M., Ishman, S.L., Kerr, L.M., Levin, A.V., Mulder, P.A., et al. (2018). Diagnosis and management of Cornelia de Lange syndrome: first international consensus statement. Nat. Rev. Genet. *19*, 649–666. https://doi.org/10.1038/s41576-018-0031-0.

Kojic, A., Cuadrado, A., De Koninck, M., Giménez-Llorente, D., Rodríguez-Corsino, M., Gómez-López, G., Le Dily, F., Marti-Renom, M.A., and Losada, A. (2018). Distinct roles of cohesin-SA1 and cohesin-SA2 in 3D chromosome organization. Nat. Struct. Mol. Biol. *25*, 496–504. https://doi.org/10.1038/s41594-018-0070-4.

Kresoja-Rakic, J., and Santoro, R. (2019). Nucleolus and rRNA Gene Chromatin in Early Embryo Development. Trends Genet. *35*, 868–879. https://doi.org/10.1016/j.tig.2019.06.005.

Laloraya, S., Guacci, V., and Koshland, D. (2000). Chromosomal addresses of the cohesin component Mcd1p. J. Cell Biol. *151*, 1047–1056. https://doi.org/10.1083/jcb.151.5.1047.

Lehalle, D., Mosca-Boidron, A.-L., Begtrup, A., Boute-Benejean, O., Charles, P., Cho, M.T., Clarkson, A., Devinsky, O., Duffourd, Y., Duplomb-Jego, L., et al. (2017). STAG1 mutations cause a novel cohesinopathy characterised by unspecific syndromic intellectual disability. J. Med. Genet. *54*, 479–488. https://doi.org/10.1136/jmedgenet-2016-104468.

Leiserson, M.D.M., Vandin, F., Wu, H.-T., Dobson, J.R., Eldridge, J.V., Thomas, J.L., Papoutsaki, A., Kim, Y., Niu, B., McLellan, M., et al. (2015). Pan-cancer network analysis identifies combinations of rare somatic mutations across pathways and protein complexes. Nat. Genet. *47*, 106–114. https://doi.org/10.1038/ng.3168.

Li, Y., Haarhuis, J.H.I., Sedeño Cacciatore, Á., Oldenkamp, R., van Ruiten, M.S., Willems, L., Teunissen, H., Muir, K.W., de Wit, E., Rowland, B.D., and Panne, D. (2020). The structural basis for cohesin-CTCF-anchored loops. Nature *578*, 472–476. https://doi.org/10.1038/s41586-019-1910-z.

Macfarlan, T.S., Gifford, W.D., Driscoll, S., Lettieri, K., Rowe, H.M., Bonanomi, D., Firth, A., Singer, O., Trono, D., and Pfaff, S.L. (2012). Embryonic stem cell potency fluctuates with endogenous retrovirus activity. Nature *487*, 57–63. https://doi.org/10.1038/nature11244.

Meshorer, E., Yellajoshula, D., George, E., Scambler, P.J., Brown, D.T., and Misteli, T. (2006). Hyperdynamic plasticity of chromatin proteins in pluripotent embryonic stem cells. Dev. Cell *10*, 105–116. https://doi.org/10.1016/j.devcel.2005.10.017.

Mishiro, T., Ishihara, K., Hino, S., Tsutsumi, S., Aburatani, H., Shirahige, K., Kinoshita, Y., and Nakao, M. (2009). Architectural roles of multiple chromatin insulators at the human apolipoprotein gene cluster. EMBO J. *28*, 1234–1245. https://doi.org/10.1038/emboj.2009.81.

Misulovin, Z., Schwartz, Y.B., Li, X.-Y., Kahn, T.G., Gause, M., MacArthur, S., Fay, J.C., Eisen, M.B., Pirrotta, V., Biggin, M.D., and Dorsett, D. (2008). Association of cohesin and Nipped-B with transcriptionally active regions of the Drosophila melanogaster genome. Chromosoma *117*, 89–102. https://doi.org/10.1007/s00412-007-0129-1.

Mootha, V.K., Lindgren, C.M., Eriksson, K.-F., Subramanian, A., Sihag, S., Lehar, J., Puigserver, P., Carlsson, E., Ridderstråle, M., Laurila, E., et al. (2003). PGC-1alpha-responsive genes involved in oxidative phosphorylation are coordinately downregulated in human diabetes. Nat. Genet. *34*, 267–273. https://doi.org/10.1038/ng1180.

Nabet, B., Roberts, J.M., Buckley, D.L., Paulk, J., Dastjerdi, S., Yang, A., Leggett, A.L., Erb, M.A., Lawlor, M.A., Souza, A., et al. (2018). The dTAG system for immediate and target- specific protein degradation. Nat. Chem. Biol. *14*, 431–441. https://doi.org/10.1038/s41589-018-0021-8.

Németh, A., Conesa, A., Santoyo-Lopez, J., Medina, I., Montaner, D., Péterfia, B., Solovei, I., Cremer, T., Dopazo, J., and Längst, G.

(2010). Initial genomics of the human nucleolus. PLoS Genet. *6*, e1000889. https://doi.org/10.1371/journal.pgen.1000889.

Orgil, O., Matityahu, A., Eng, T., Guacci, V., Koshland, D., and Onn, I. (2015). A conserved domain in the scc3 subunit of cohesin mediates the interaction with both mcd1 and the cohesin loader complex. PLoS Genet. *11*, e1005036. https://doi.org/10.1371/journal.pgen.1005036.

Padeken, J., and Heun, P. (2014). Nucleolus and nuclear periphery: velcro for heterochromatin. Curr. Opin. Cell Biol. *28*, 54–60. https://doi.org/10.1016/j.ceb.2014.03.001.

Parelho, V., Hadjur, S., Spivakov, M., Leleu, M., Sauer, S., Gregson, H.C., Jarmuz, A., Canzonetta, C., Webster, Z., Nesterova, T., et al. (2008). Cohesins functionally associate with CTCF on mammalian chromosome arms. Cell *132*, 422–433. https://doi.org/10.1016/j.cell.2008.01.011.

Percharde, M., Lin, C.-J., Yin, Y., Guan, J., Peixoto, G.A., Bulut-Karslioglu, A., Biechele, S., Huang, B., Shen, X., and Ramalho-Santos, M. (2018). A LINE1-Nucleolin Partnership Regulates Early Development and ESC Identity. Cell *174*, 391–405.e19. https://doi.org/10.1016/j.cell.2018.05.043.

Phillips-Cremins, J.E., Sauria, M.E.G., Sanyal, A., Gerasimova, T.I., Lajoie, B.R., Bell, J.S.K., Ong, C.-T., Hookway, T.A., Guo, C., Sun, Y., et al. (2013). Architectural protein subclasses shape 3D organization of genomes during lineage commitment. Cell *153*, 1281–1295. https://doi.org/10.1016/j.cell.2013.04.053.

Quinodoz, S.A., Ollikainen, N., Tabak, B., Palla, A., Schmidt, J.M., Detmar, E., Lai, M.M., Shishkin, A.A., Bhat, P., Takei, Y., et al. (2018). Higher-Order Inter-chromosomal Hubs Shape 3D Genome Organization in the Nucleus. Cell *174*, 744–757.e24. https://doi.org/10.1016/j.cell.2018.05.024.

Rao, S.S.P., Huang, S.-C., Glenn St Hilaire, B., Engreitz, J.M., Perez, E.M., Kieffer-Kwon, K.-R., Sanborn, A.L., Johnstone, S.E., Bascom, G.D., Bochkov, I.D., et al. (2017). Cohesin Loss Eliminates All Loop Domains. Cell *171*, 305–320.e24. https://doi.org/10.1016/j.cell.2017.09.026.

Rao, S.S.P., Huntley, M.H., Durand, N.C., Stamenova, E.K., Bochkov, I.D., Robinson, J.T., Sanborn, A.L., Machol, I., Omer, A.D., Lander, E.S., and Aiden, E.L. (2014). A 3D map of the human genome at kilobase resolution reveals principles of chromatin looping. Cell *159*, 1665–1680. https://doi.org/10.1016/j.cell.2014.11.021.

Remeseiro, S., Cuadrado, A., Carretero, M., Martínez, P., Drosopoulos, W.C., Cañamero, M., Schildkraut, C.L., Blasco, M.A., and Losada, A. (2012a). Cohesin-SA1 deficiency drives aneuploidy and tumourigenesis in mice due to impaired replication of telomeres. EMBO J. *31*, 2076–2089. https://doi.org/10.1038/emboj.2012.11.

Remeseiro, S., Cuadrado, A., Gómez-López, G., Pisano, D.G., and Losada, A. (2012b). A unique role of cohesin-SA1 in gene regulation and development. EMBO J. *31*, 2090–2102. https://doi.org/10.1038/emboj.2012.60.

Romero-Pérez, L., Surdez, D., Brunet, E., Delattre, O., and Grünewald, T.G.P. (2019). STAG Mutations in Cancer. Trends Cancer *5*, 506–520. https://doi.org/10.1016/j.trecan.2019.07.001.

Rowe, H.M., Jakobsson, J., Mesnard, D., Rougemont, J., Reynard, S., Aktas, T., Maillard, P.V., Layard-Liesching, H., Verp, S., Marquis, J., et al. (2010). KAP1 controls endogenous retroviruses in embryonic stem cells. Nature *463*, 237–240. https://doi.org/10.1038/nature08674.

Schwalie, P.C., Ward, M.C., Cain, C.E., Faure, A.J., Gilad, Y., Odom, D.T., and Flicek, P. (2013). Co-binding by YY1 identifies the transcriptionally active, highly conserved set of CTCF-bound regions in primate genomes. Genome Biol. *14*, R148. https://doi.org/10.1186/gb-2013-14-12-r148.

Schwarzer, W., Abdennur, N., Goloborodko, A., Pekowska, A., Fudenberg, G., Loe-Mie, Y., Fonseca, N.A., Huber, W., Haering, C.H., Mirny, L., and Spitz, F. (2017). Two independent modes of chromatin organization revealed by cohesin removal. Nature *551*, 51–56. https://doi.org/10.1038/nature24281.

Seitan, V.C., Faure, A.J., Zhan, Y., McCord, R.P., Lajoie, B.R., Ing-Simmons, E., Lenhard, B., Giorgetti, L., Heard, E., Fisher, A.G., et al. (2013). Cohesin-based chromatin interactions enable regulated gene expression within preexisting architectural compartments. Genome Res. *23*, 2066–2077. https://doi.org/10.1101/gr.161620.113.

Soardi, F.C., Machado-Silva, A., Linhares, N.D., Zheng, G., Qu, Q., Pena, H.B., Martins, T.M.M., Vieira, H.G.S., Pereira, N.B., Melo-Minardi, R.C., et al. (2017). Familial STAG2 germline mutation defines a new human cohesinopathy. NPJ Genom. Med. *2*, 7–11. https://doi.org/10.1038/s41525-017-0009-4.

Sofueva, S., Yaffe, E., Chan, W.-C., Georgopoulou, D., Vietri Rudan, M., Mira-Bontenbal, H., Pollard, S.M., Schroth, G.P., Tanay, A., and Hadjur, S. (2013). Cohesin-mediated interactions organize chromosomal domain architecture. EMBO J. *32*, 3119–3129. https://doi.org/10.1038/emboj.2013.237.

Subramanian, A., Tamayo, P., Mootha, V.K., Mukherjee, S., Ebert, B.L., Gillette, M.A., Paulovich, A., Pomeroy, S.L., Golub, T.R., Lander, E.S., and Mesirov, J.P. (2005). Gene set enrichment analysis: a knowledge-based approach for interpreting genome-wide expression profiles. Proc. Natl. Acad. Sci. USA *102*, 15545–15550. https://doi.org/10.1073/pnas.0506580102.

Sun, Z., Yu, H., Zhao, J., Tan, T., Pan, H., Zhu, Y., Chen, L., Zhang, C., Zhang, L., Lei, A., et al. (2022). LIN28 coordinately promotes nucleolar/ribosomal functions and represses the 2C-like transcriptional program in pluripotent stem cells. Protein Cell *13*, 490–512. https://doi.org/10.1007/s13238-021-00864-5.

Tapial, J., Ha, K.C.H., Sterne-Weiler, T., Gohr, A., Braunschweig, U., Hermoso-Pulido, A., Quesnel-Vallières, M., Permanyer, J., Sodaei, R., Marquez, Y., et al. (2017). An atlas of alternative splicing profiles and functional associations reveals new regulatory programs and genes that simultaneously express multiple major isoforms. Genome Res. *27*, 1759–1768. https://doi.org/10.1101/gr.220962.117.

Vietri Rudan, M., Barrington, C., Henderson, S., Ernst, C., Odom, D.T., Tanay, A., and Hadjur, S. (2015). Comparative Hi-C reveals that CTCF underlies evolution of chromosomal domain architecture. Cell Rep. *10*, 1297–1309. https://doi.org/10.1016/j.celrep.2015.02.004.

Viny, A.D., Bowman, R.L., Liu, Y., Lavallée, V.P., Eisman, S.E., Xiao, W., Durham, B.H., Navitski, A., Park, J., Braunstein, S., et al. (2019). Cohesin Members Stag1 and Stag2 Display Distinct Roles in Chromatin Accessibility and Topological Control of HSC Self-Renewal

and Differentiation. Cell Stem Cell *25*, 682–696.e8. https://doi.org/10.1016/j.stem.2019.08.003.

Wendt, K.S., Yoshida, K., Itoh, T., Bando, M., Koch, B., Schirghuber, E., Tsutsumi, S., Nagae, G., Ishihara, K., Mishiro, T., et al. (2008). Cohesin mediates transcriptional insulation by CCCTC-binding factor. Nature *451*, 796–801. https://doi.org/10.1038/nature06634.

Winters, T., McNicoll, F., and Jessberger, R. (2014). Meiotic cohesin STAG3 is required for chromosome axis formation and sister chromatid cohesion. EMBO J. *33*, 1256–1270. https://doi.org/10.1002/embj.201387330.

Wutz, G., Várnai, C., Nagasaka, K., Cisneros, D.A., Stocsits, R.R., Tang, W., Schoenfelder, S., Jessberger, G., Muhar, M., Hossain, M.J., et al. (2017). Topologically associating domains and chromatin loops depend on cohesin and are regulated by CTCF, WAPL, and PDS5 proteins. EMBO J. *36*, 3573–3599. https://doi.org/10.15252/embj.201798004.

Xiao, T., Wallace, J., and Felsenfeld, G. (2011). Specific sites in the C-terminus of CTCF interact with the SA2 subunit of the cohesin complex and are required for cohesin-dependent insulation activity. Mol. Cell Biol. *31*, 2174–2183. https://doi.org/10.1128/MCB.05093-11.

Xie, S.Q., Leeke, B.J., Whilding, C., Wagner, R.T., Garcia-Llagostera, F., Low, Y., Chammas, P., Cheung, N.T.-F., Dormann, D., McManus, M.T., and Percharde, M. (2022). Nucleolar-based Dux repression is essential for embryonic two-cell stage exit. Genes Dev. *36*, 331–347. https://doi.org/10.1101/gad.349172.121.

Yao, R.-W., Xu, G., Wang, Y., Shan, L., Luan, P.-F., Wang, Y., Wu, M., Yang, L.-Z., Xing, Y.-H., Yang, L., and Chen, L.-L. (2019). Nascent Pre-rRNA Sorting via Phase Separation Drives the Assembly of Dense Fibrillar Components in the Human Nucleolus. Mol. Cell *76*, 767–783.e11. https://doi.org/10.1016/j.molcel.2019.08.014.

Yuan, B., Neira, J., Pehlivan, D., Santiago-Sim, T., Song, X., Rosenfeld, J., Posey, J.E., Patel, V., Jin, W., Adam, M.P., et al. (2019). Clinical exome sequencing reveals locus heterogeneity and phenotypic variability of cohesinopathies. Genet. Med. *21*, 663–675. https://doi.org/10.1038/s41436-018-0085-6.

Zuin, J., Dixon, J.R., van der Reijden, M.I.J.A., Ye, Z., Kolovos, P., Brouwer, R.W.W., van de Corput, M.P.C., van de Werken, H.J.G., Knoch, T.A., van IJcken, W.F.J., et al. (2014). Cohesin and CTCF differentially affect chromatin architecture and gene expression in human cells. Proc. Natl. Acad. Sci. USA *111*, 996–1001. https://doi.org/10.1073/pnas.1317788111.

Stem Cell Reports, Volume *18*

Supplemental Information

The N-terminus of *Stag1* is required to repress the 2C program by maintaining rRNA expression and nucleolar integrity

Dubravka Pezic, Samuel Weeks, Wazeer Varsally, Pooran S. Dewari, Steven Pollard, Miguel R. Branco, and Suzana Hadjur

Supplementary Figure 1. *Pezic et al.*

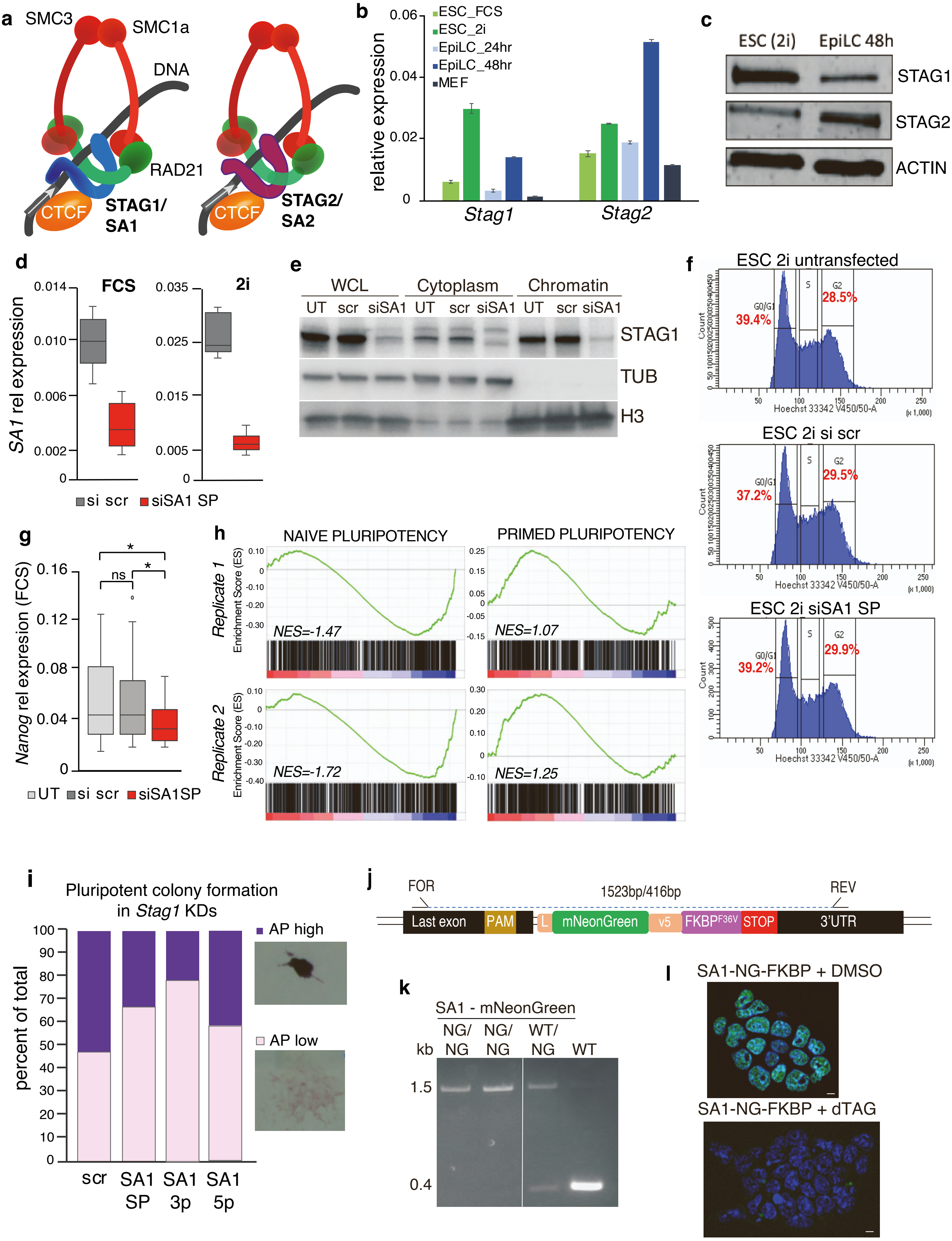

Supplementary Figure 2. *Pezic et al.*

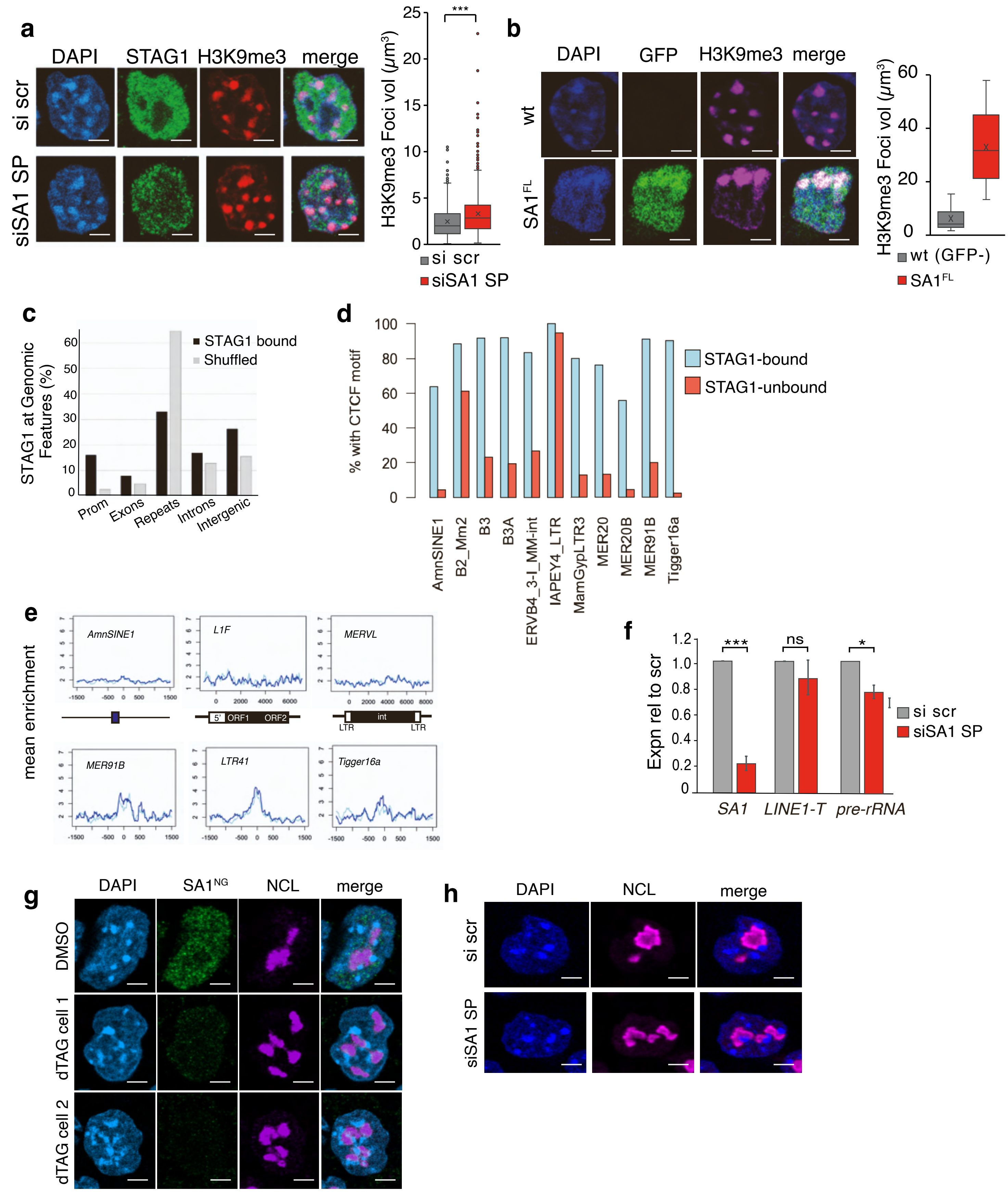

Supplementary Figure 3. *Pezic et al.*

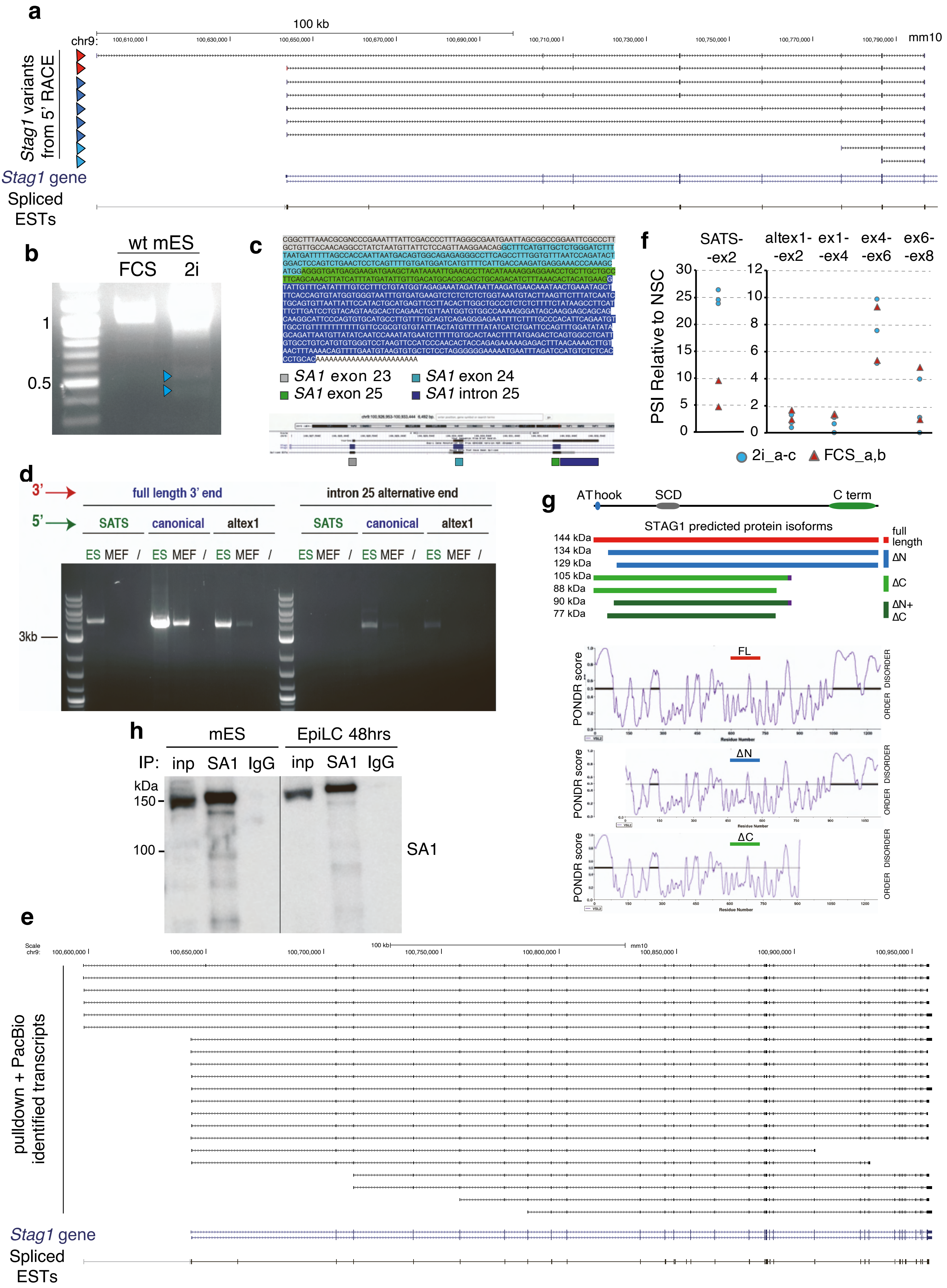

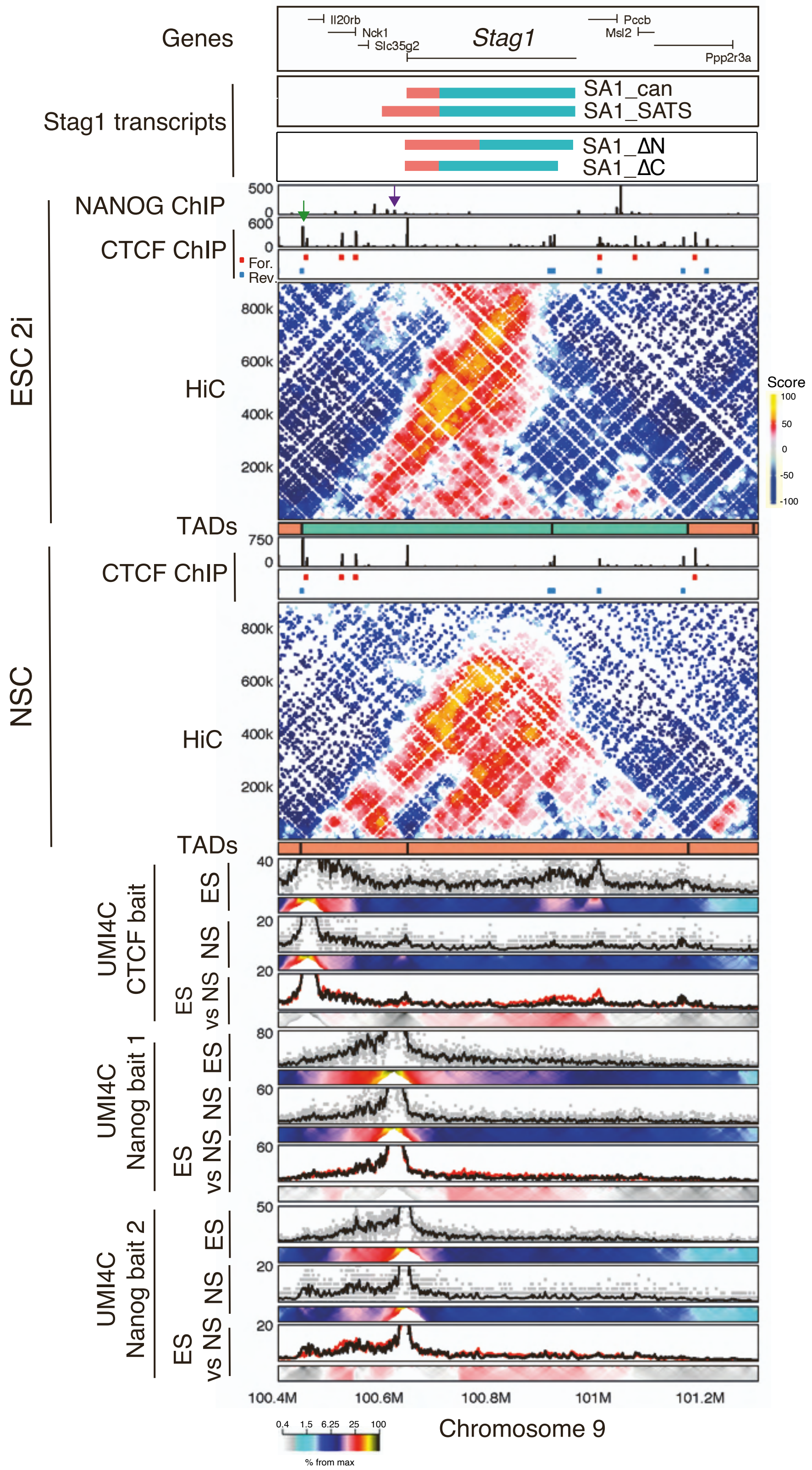

Genes
Il20rb
Nck1
Slc35g2
Stag1
Pccb
Msl2
Ppp2r3a
Stag1 transcripts
SA1_can
SA1_SATS
SA1_ΔN
SA1_ΔC
ESC 2i
NANOG ChIP
CTCF ChIP
For.
Rev.
HiC
TADs
Score
NSC
UMI4C CTCF bait
UMI4C Nanog bait 1
UMI4C Nanog bait 2
ES
NS
ES vs NS
% from max
100.4M
100.6M
100.8M
101M
101.2M
Chromosome 9

Supplementary Figure 5. *Pezic et al.*

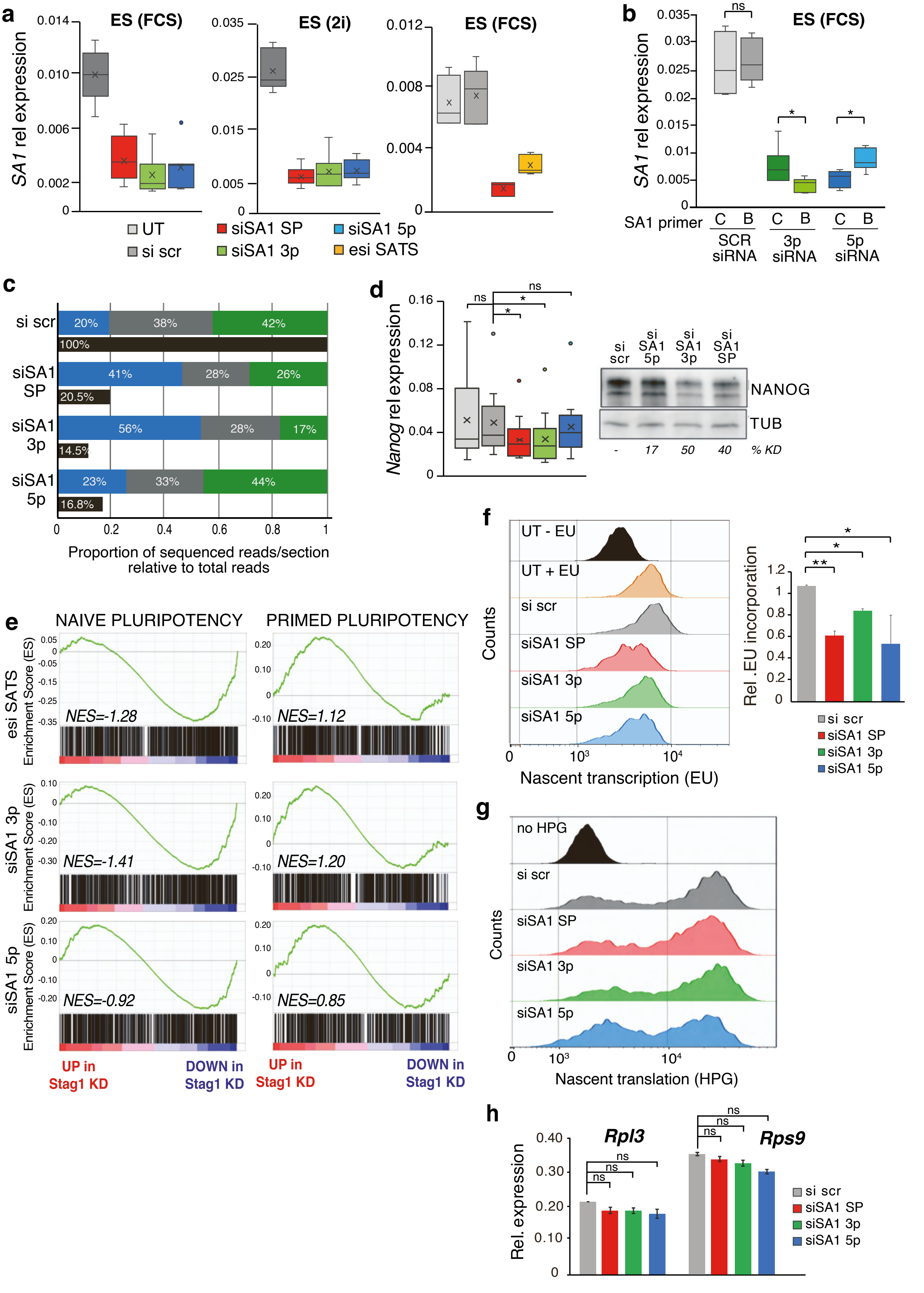

Supplementary Figure 6. *Pezic et al.*

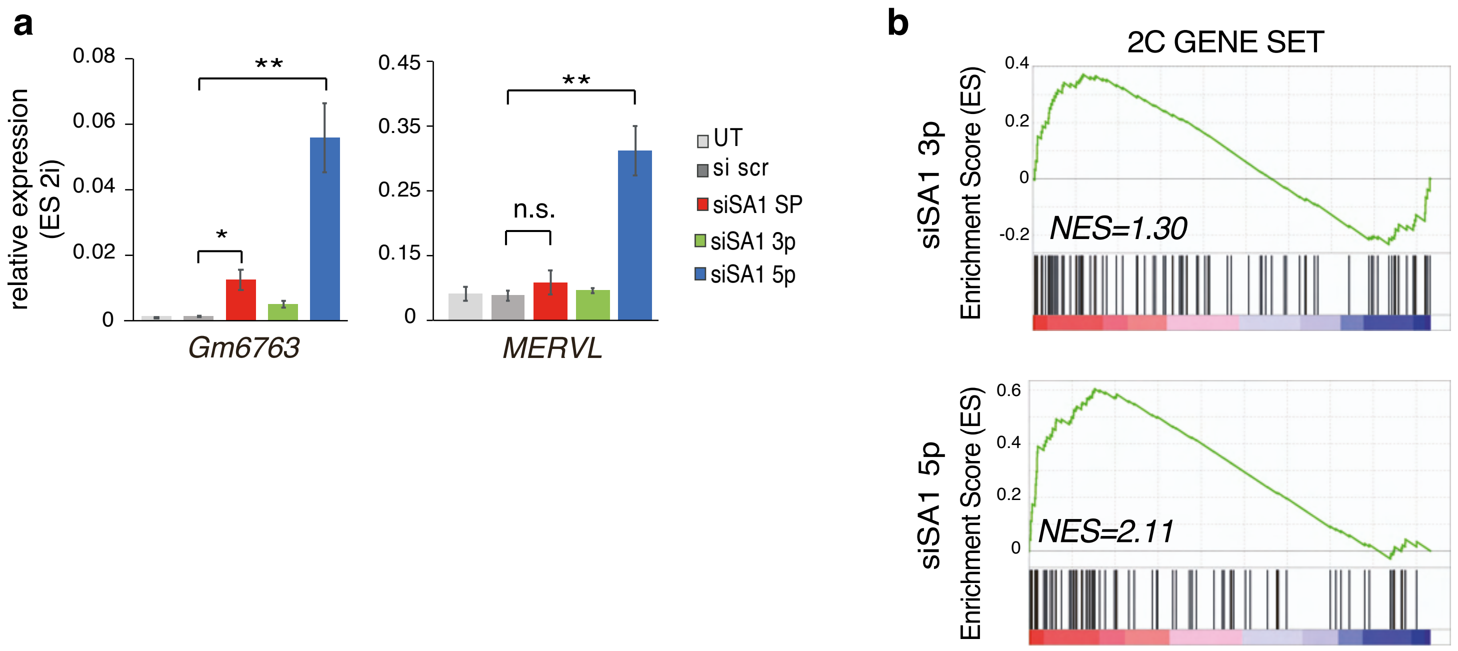

SUPPLEMENTARY FIGURE LEGENDS

Supplementary Figure 1. *Stag1* is required for pluripotency in mESCs. Related to Figure 1.

a) Cartoon of the cohesin complex including the core trimer subunits of SMC1a, SMC3 and RAD21 complexed with either STAG1 or STAG2.

b) Relative expression of *Stag1* and *Stag2* mRNA by qRT-PCR in 2i- (naïve) or FCS-grown mESC, EpiLCs and MEFs. Data is represented as mean ± SEM of two independent experiments and relative to *Actin* expression.

c) WCL from naïve (2i) mESC and EpiLCs, sorted for cells in the G1 phase and analysed by WB for levels of STAG1 and STAG2. ACTIN serves as a loading control.

d) Relative expression of *Stag1* mRNA by qRT-PCR in FCS- (left panel, n=20) or 2i-grown (right panel, n=19) mESCs upon treatment with si scr or si SA1, smartpool (SP). Whiskers and boxes indicate all and 50% of values respectively. Central line represents the median. Data is n=10 (FCS) and n=8 (2i) independent experiments.

e) WB analysis of STAG1 levels in WCL, cytoplasmic and chromatin fractions upon treatment with scrambled siRNAs (si Scr) or SA1 siRNAs (siSA1) for 24hr in naïve mESCs. Data as in Fig. 1c, but also including the cytoplasmic fraction. Tubulin (TUB) and H3 as controls.

f) Cell cycle analysis of Hoechst-stained 2i mESC after treatment with si scr or siSA1siRNAs for 24hrs. Shown are the percentages of cells in G1 or G2 phases. These are the same cells that were used for the RNA-sequencing experiments shown in Fig. 1.

g) Relative expression of *Nanog* mRNA by qRT-PCR in FCS-grown mESCs upon treatment with si scr or siSA1. Quantification and statistics as before. Data is from five independent experiments.

h) Enrichment score (ES) plots from GSEA using the naïve or primed gene sets as in Fig. 1 and RNA-seq from two additional siSA1 mESC biological replicates. The third replicate is shown in Fig. 1.

i) AP^{hi} colonies in mESCs (purple), as a percentage of all colonies (pink and purple) treated with the siRNA panel. Data are the average of three independent replicates.

j, k) Cartoon of the CRISPR/Cas9 targeting strategy to introduce a NeonGreen-v5-FKBP tag to the C-terminus of endogenous *Stag1*. FOR and REV primers used for genotyping. The leftmost NG/NG homozygous sample represents the 'B1 clone' used in the manuscript.

l) Representative confocal images of neon-green in $SA1^{NG\text{-}FKBP}$ mESC (clone B1) treated with DMSO or dTAG. Scale bar, 3 microns.

Supplementary Figure 2. STAG1 is localised to and impacts both euchromatin and heterochromatin compartments. Related to Figure 2.

a) Left, representative confocal images of IF to STAG1 and H3K9me3 in siRNA-treated mESC counterstained with DAPI. Right, Imaris quantification of the volume of H3K9me3 foci from siRNA treated mESC. Quantifications and statistical analysis were done as above. Data is from n>140 independent cells/condition in three biological replicates. Scale bar, 3 microns.

b) Left, representative confocal images of IF to GFP and H3K9me3 in mESCs expressing a dox-inducible GFP-tagged full-length STAG1 (SA1-FL) and counterstained with DAPI. Right, Imaris quantification of the volume of H3K9me3 foci from dox-inducible mESCs. Quantifications and statistical analysis as above. Data is from n>80 independent cells/condition in two replicates.

c) Percentage of STAG1 ChIP-seq peaks in mESCs (unique reads) at promoters, exons, transposable element repeats, introns and intergenic sequences.

d) Analysis of CTCF motifs contained within selected repeat elements and the percentage of STAG1 binding. NB. The majority of elements contain both a CTCF motif and STAG1.

e) STAG1 ChIP-seq (unique and multimapping reads) aligned to additional full-length repeat elements. Two STAG1 ChIP replicates are shown in blue alongside the INPUT in grey.

f) Relative expression of *Stag1*, *LINE1-T* and *pre-rRNA* by qRT-PCR in siRNA-treated mESCs. Shown are total RNA levels. Data is represented as mean ± SEM and statistical analysis as before. Data is from three independent experiments.
Representative confocal images of MFI of NCL assessed by IF in g) $SA1^{NG\text{-}FKBP}$ mESC (clone B1) treated with DMSO or dTAG (two different cells shown) and h) siRNA-treated mESC and counterstained with DAPI. Quantification of these is shown in Fig. 2k. Scale bar, 3 microns.

Supplementary Figure 3. Transcription-regulatory control of *Stag1* in mESC. Related to Fig 3.
a) Aligned *Stag1* transcript variants identified from 5'RACE in Fig. 3a. Arrows refer to the bands on the RACE gels which were cloned and sequenced. *NB,* the diversity of skipping events that all result in a functional loss of the 5' end of *Stag1*.
b) Over-exposure of 5'RACE gel shown in Fig. 3a (right) to show small RACE products (blue arrows).
c) Close-up of the 3' RACE sequence that identified a new alternative TTS in intron 25 (sequence shown in dark blue).
d) PCR mini-screen in naïve mESCs and MEFs using various combinations of forward (5') primers (SATS, canonical TSS, Alt exon 1 TSS) and reverse (3') primers (canonical TTS, Alt intron 25 TTS). *NB.* SATS is only expressed in ESC; canonical, full-length *Stag1* is more expressed in ESC compared to MEFs; and the alternative intron 25 TTS is most often expressed with a canonical TSS.
e) *Stag1* transcripts sequenced on the PacBio platform. Including many isoforms that were already discovered using RACE and PCR cloning methods above.
f) Percent Spliced In (PSI) calculations based on VAST-Tools analysis of RNA-seq from multiple 2i (blue) and FCS (red) datasets (see Methods). Data are shown relative to Neural stem cell (NSC) frequencies, highlighting the events that are ESC-specific.
g) Top, cartoon depicting functional domains within STAG1 protein, including the AT-hook (aa 3-58); Stromalin conserved domain (SCD, aa 296-381) and the C-terminus. Middle, the predicted STAG1 protein isoforms based on transcript analysis with estimated sizes for each isoform and colour coded according to the analysis in Fig 3d. Purple boxes in the 105kDa and 90kDa isoforms represent retained introns. Bottom, PONDR (Predictor of Natural Disordered Regions) analysis of STAG1 using VSL2 predictor showing consecutive stretches of disordered regions corresponding to the N- and C-terminus of STAG1 in its full-length (FL), N-terminal (ΔN) and C-terminal delta (ΔC) isoform groups.
h) Chromatin Immunoprecipitation of endogenous STAG1 in mESCs and EpiLCs. IgG was used as a control. *NB.* Both canonical and STAG1 isoform levels are reduced upon differentiation.

Supplementary Figure 4. Genome topology at the *Stag1* locus. Related to Figure 3.
Hi-C contact maps in naïve mESC and NSC of the 900kb region on chromosome 9 containing the *Stag1* topologically associated domain (TAD). TADs are denoted with a vertical line and as repressed (orange) or active (blue). Shown also are tracks for NANOG and CTCF ChIP-seq as well as a track indicating the directionality of CTCF binding sites (red, forward; blue, reverse). Aligned to the gene track are also the *Stag1* transcripts discovered above where red represents the untranslated regions and blue the coding body. UMI-4C-seq viewpoints are positioned to the leftmost CTCF site ('CTCF bait', vertical green arrow on ChIP track) and to a Nanog site 40 kb upstream of the *Stag1* canonical TSS ('Nanog bait', vertical purple arrow on ChIP track). For each bait, UMI information for each cell type is shown as well as the comparative plots where red represents an enrichment of contacts in ESC compared to NSC.

Supplementary Figure 5. Fluctuations in *Stag1* isoforms skews cell fates. Related to Figure 4.
a) Relative expression of *Stag1* mRNA by qRT-PCR using SA1_A primer in FCS- (n=7) or 2i-grown (n=6) mESC upon si scr or the si SA1 panel. Quantifications as before.

b) Relative expression of *Stag1* mRNA by qRT-PCR using two different primers located at either the 5' end (SA1_C) or the 3' end (SA1_B) of *Stag1* mRNA in mESCs (n=6) treated with scr, 3p or 5p siRNAs. While both siRNA sets reduce *Stag1* to a similar amount overall, the relative proportion of the residual 5' or 3' ends is significantly different in the 3p or 5p KDs. * $p<0.05$.
c) Data as in Fig. 4c shown here for the second biological replicate siRNA KD panel (same samples as the GSEA in Fig S5e).
d) Left, relative expression of *Nanog* mRNA by qRT-PCR in FCS-grown mESCs upon si scr or the si SA1 panel (n=13). Quantifications as before. *NB,* the modest, but different influence of the 5p and the 3p KDs on *Nanog* levels. Right, WB analysis of NANOG levels in siRNA treated mESC WCL. TUB is loading control. The percentage of KD of NANOG signal normalised to TUB is shown.
e) Enrichment score (ES) plots from GSEA using the naïve or primed gene sets as in Fig. 1f and RNA-seq data from the second set of mESCs treated with the siRNAs to SATS TSS, 3p and 5p.
f) Global analysis of nascent transcription by measuring EU-488 incorporation using Flow cytometry. Left, representative Flo-Jo analysis of EU incorporation in mESCs treated with the siRNA KD panel and controls. Right, quantification of the change in EU incorporation relative to si scr treated cells. Data are represented as the mean +/- SEM and are from three independent replicates. Statistical analysis using two-tailed t-test.
g) Global analysis of nascent translation by measuring HPG incorporation using Flow cytometry. Shown is representative Flo-Jo analysis of HPG incorporation in mESC treated with the siRNA KD panel. Quantifications of the data can be found in Fig. 5f.
h) Relative expression of *Rpl3* and *Rps9* mRNA by qRT-PCR in mESC upon si scr or the si SA1 panel. Data are represented as the mean +/- SEM and are from three independent replicates. Statistical analysis using two-tailed t-test.

Supplementary Figure 6. Loss of the *Stag1* N-terminus leads to conversion to totipotency.
a) Relative expression of 2C related genes by qRT-PCR in 2i-grown mESC after treatment with si scr or the si SA1 panel. Data are represented as mean +/- SEM from n=5 independent replicates.
b) Enrichment score (ES) plots from GSEA using 2C gene sets as in Fig. 6e and the replicate RNA-seq data from the siRNA treated mESC samples.

SUPPLEMENTARY METHODS and TABLES

GSEA - Broad Institute GSEA Preranked (v4.0.3) was used to determine the enrichment of curated genesets within our RNA-seq data. For each sample a ranked list was generated with genes ranked in descending order by their log2FC value using normalised expression scores from DEseq2. Log2FC per gene was calculated between the KD and its respective SCR using the following calculation: Log2(normalised_counts KD +1) - log2(normalised_counts SCR +1). In the case of experiments with multiple KD replicates, the average log2 normalised count was used. Three gene sets were assayed in this study, 'naïve pluripotency', 'primed pluripotency' and '2C signatures'. The naïve and primed pluripotency gene sets were curated in-house from (Fidalgo et al., 2016) where genes were selected if they had $\geq$2 fold change. The naïve and primed gene sets contained 661 and 580 genes respectively. The 2C signatures gene set (147 genes) was obtained from (Percharde et al., 2018). Gene sets were classed as having significant enrichment if the p-value was $\leq$0.05 and the normalised enrichment score (NES) exceeded +/- 1.

CRISPR-Mediated *Stag1* Knock-in Cell Line Generation - The guide RNA targeting Stag1 3' terminal coding region was designed using Tagin Software (http://tagin.stembio.org) and purchased from IDT. Lyophilised gRNA was rehydrated in RNA duplex buffer (100μM). The single stranded

oligodeoxynucleotides (ssODN) encoding mNeonGreen (mNG)-V5-FKBP12^{F36V} and the left and right homology arms was designed using the software tool ChopChop (https://chopchop.cbu.uib.no) and purchased as a High-Copy Amp-resistant plasmid from Twist Bioscience. 2.2μl gRNA (100μM) was mixed with 2.2μl tracrRNA ATTO 550nm (IDT) and annealed together. The RNA duplex was then incubated with 20μg S.p Cas9 Nuclease V3 (IDT) for 10min at room temperature and stored on ice prior to transfection. Linearised KI sequence was mixed with 100% DMSO and denatured at 95°C for 5min. The ssODN was plunged immediately into ice. The RNP complex was mixed with confluent 2i-grown ES cells re-suspended in P3 transfection buffer (Lonza) before being transferred to an electroporation microcuvette well (Lonza). Transfection was performed using a 4D Amaxa electroporator. Post-nucleofection, the cells were seeded into a fibronectin-coated 6 well plate with fresh ESC media. The media was changed daily for four days before being expanded into a T75 flask. Confluent ESC were FACS sorted for GFP+ population (BD FACS Aria Fusion Cell Sorter) and sparsely seeded into 10 cm plates. Clones were manually picked into 96 well plates and expanded for selection by v5 IF, genotyping and Sanger sequencing.

Dox-inducible STAG1-GFP isoform cell lines - Stag1 isoforms were cloned into pCW57.1 vector (Addgene 41393), modified using Gibson assembly to include an EGFP tag at the 3'end of the Gateway cassette, using Gateway recombination by LR clonase. For primers used to clone the isoforms see Supplementary Table S3. Plasmids were transfected into 2i-grown ESCs using Lipofectamine 3000 and cells grown in Puromycin-supplemented media (1μg/ml) for ten days to make stable lines. Isoform expression was induced using 2μg/ml Doxycycline for 24 hrs, and the population enriched for GFP-positive cells using FACS. For IF experiments, induction with Dox was for 48 hours.

VAST-TOOLS - To generate Percent Spliced In (PSI) scores, a statistic which represents how often a particular exon is spliced into a transcript using the ratio between reads which include and exclude said exon was used (Tapial et al., 2017). Paired-end RNA-seq datasets were submitted to VAST-TOOLS (v2.1.3) using the Mmu genome. Briefly, reads are split into 50nt words with a 25nt sliding window. The 50nt words are aligned to a reference genome using Bowtie to obtain unmapped reads. These unmapped reads are then aligned to a set of predefined exon-exon junction (EJJ) libraries allowing for the quantification of alternative exon events. The output was further interrogated using a script which searches all hypothetical EEJ combinations between potential donors and acceptors within Stag1. PSI scores could be obtained providing there was at least a single read within our RNAseq data that supported one of these potential events. Some datasets were combined to have enough reads for the analysis. See Table S1.

Table S1. Percent Spliced In (PSI) Values

Source of RNA-seq data	Name in Figure	SATS-exon2	AltEx1 - exon2	exon1 - exon4	exon4 - exon6	exon6 - exon8	exon21 - AltEx22
this study, 'ES 2i_Rep1_enriched'	2i_a	23.68	5.26	1.29	10.34	1.03	0
this study, 'ES 2i_Rep1'	2i_b	21.43	9.52	0	7.89	0	0
doi.org/10.1016/j.stemcr.2018.08.001	2i_c	22.08	2.60	2.13	5.36	3.56	0
doi:10.1016/j.cell.2017.09.043	FCS_a	8.62	12.07	2.63	9.73	0	0.81
this study, 'ES FCS_Rep2/3_CTL/SCR' combined	FCS_b	4.25	6.95	2.58	5.63	0.91	1.11
doi:10.1016/j.cell.2017.09.043	NSC (rel to)	0.98	7.14	1.92	1.05	0	0

Quantifying sectioned *Stag1* and PONDR - Stag1 was split into 5 sections; SATS, e1-e8, e12-e19, e20-e25, e26-e34. Using Kallisto (v0.46.1), raw RNAseq reads were used to quantify each section of Stag1. Kallisto was run in quant mode, using the –rf-stranded parameter, outputting a TPM per Stag1 section. TPMs were normalised to the total reads from the 3 sections analysed and expressed as a percent. Internally disordered regions were predicted using VSL2 predictor at http://www.pondr.com.

Immunofluorescence and Microscopy - mESCs were cultured on fibronectin or gelatin-coated cover glass in 6-well plates. Cells were fixed in 4% Paraformaldehyde for 5min and incubated in 0.1% Triton X-PBS for 10min before being washed and blocked in 10% FCS-PBS for 20min. Primary antibodies were diluted in 10% FCS, 0.1% Saponin (Sigma) and incubated overnight at 4°C. The next day, the cells were incubated with an Alexa fluorophore-conjugated secondary antibody diluted in 10% FCS, 0.1% Saponin for 1 hr at room temperature, washed and mounted on cover slides with ProLong Diamond Antifade Mountant with DAPI (Invitrogen). Z-stacks imaging of fixed cells was done using a LSM 880 confocal microscope (Zeiss) with a 63X oil objective. Analysis was performed using Imaris 9.6 (Oxford instruments). Live cell imaging was performed using a 3i Spinning Disc confocal microscope (Zeiss). Stag1-mNG-V5-FKBP12^{F36V} cells were seeded in an 8-chambered coverglass (Lab-Tek II) and DMSO or dTAG (500nM) were added for 24hr before imaging. Directly prior to imaging, cells were incubated with Hoechst 33342 (BD Pharmingen) for 45min, and then replaced with fresh 2i ESC media. Cells were imaged as confocal Z-stacks using DAPI and GFP lasers with a 63X objective and 1.4 Numerical Aperture.

Antibodies - STAG1/SA1, N-term epitope (Abcam, ab4455); STAG1/SA1, C-term epitope (Abcam, ab4457); STAG2/SA2 (Bethyl, A300-158A); SMC3 (Abcam, ab9263); NANOG (Abcam, ab70482); Tubulin/TUB (Sigma, T5168); ACTIN (Novus, Mab8929); H3 (Abcam, ab1791); v5 (Invitrogen, 14-6796-82); HP1a (Cell Signalling, 2616); Nucleolin/NCL (Abcam, ab22758); POLR2 (Covance, MMS-128P); H3K9me3 (Abcam, ab8898); H3K4me3 (Abcam, ab8580); Alexa488-anti-GFP (GFP) (ThermoFisher, A-21311); TRIM28 (ThermoFisher, MA1-2023).

Protein Lysates, Fractionations and Western blotting - Whole cell lysates (WCL) were collected by lysis in RIPA buffer (150mM NaCl, 1% NP-40 detergent, 0.5% Sodium Deoxycholate, 0.1% SDS, 25mM Tris-HCl pH 7.4, 1mM DTT) and sonicated at 4°C for x5 30 second cycles using Diagenode Bioruptor. Insoluble material was pelleted and the supernatant lysate was quantified using BSA Assay (Thermo Scientific). For cellular fractionations, a cellular ratio of $5x10^6$ cells/80μl buffer was maintained throughout the protocol. Cells were re-suspended in Cell Membrane Lysis Buffer (0.1% Triton X, 10mM HEPES pH 7.9, 10mM KCl, 1.5mM MgCl2, 0.34M sucrose, 10% glycerol, 1mM DTT), incubated on ice for 5min and centrifuged for 5min at 3700rpm to collect the cytoplasmic sample. The pellet was re-suspended in Nuclear Lysis Buffer (3mM EDTA, 0.2mM EGTA, 1mM DTT) and incubated on ice for 1 hr. Nuclear lysis was aided by sonication with a handheld homogeniser (VWR) for 10sec at 10min intervals. The nucleoplasmic supernatant and chromatin pellet were separated by centrifugation at 9000rpm for 10min at 4°C. The chromatin pellet was re-suspended in 160μl 2X Laemmli Buffer (Bio-Rad). Equal volumes of each fraction were used for WB. Cyto- and nucleoplasmic protein samples were diluted in 2X Laemmli Buffer and boiled for 5min, then loaded on a 4-20% SDS-PAGE gel (Bio-rad) or a 3-8% Tris Acetate gel (Invitrogen). Proteins were wet transferred onto a PDVF membrane (Millipore) and assessed with Ponceau Red (Sigma). The membrane was blocked with 10% milk and incubated with primary antibodies in 1% milk, 0.1% Tween-PBS overnight at 4°C. Membranes were imaged with SuperSignal West Femto Maximum Sensitivity (Thermo) on ImageQuant.

Table S2. Mass Spectrometry Analysis of STAG1 IP bands. Accession Q9D3E6

Band	Coverage	Peptide Sequence	Peptide AA position	PEP score
145 kDa	29.35%	HVESDVLEACSK	627-638	0.00005153
		ITDGSPSKEDLLVLR	752-766	0.00003838
140 kDa	27.34%	HDPQAEEALAKR	443-454	0.0002167
		ITDGSPSKEDLLVLR	752-766	0.0002349
130 kDa	19.27%	NMQNAEIIRK	141-150	0.0001146
		HTSTLAAMK	220-228	0.0005888
100 kDa	17.40%	HTSTLAAMK	220-228	0.01277
		HVESDVLEACSK	627-638	0.0003382
75 kDa	14.94%	HTSTLAAMK	220-228	0.001847
		LTSFHNAHDLTK	698-709	2.032E-15

Chromatin Co-Immunoprecipitation (co-IP) - Cells were re-suspended in 0.1% NP-40-PBS (1ml/1x10^7 cells) with 1X Protease Inhibitors (Roche) and 1mM DTT, and centrifuged at 1500rpm for 2min at 4°C. The pellet was re-suspended in Nuclear Lysis Buffer (3mM EDTA, 0.2mM EGTA, 1X Protease Inhibitors, 1mM DTT), vortexed for 30sec before being incubated on a rotator for 30min at 4°C and centrifuged at 6500g for 5min at 4°C to isolate the glassy chromatin pellet. This was re-suspended in High Salt Chromatin Solubilisation Buffer (50mM Tris-HCl pH 7.5, 1.5mM MgCl2, 300mM KCl, 20% glycerol, 1mM EDTA, 0.1% NP-40, 1mM Pefabloc, 1X Protease Inhibitors, 1mM DTT) with Benzonase (Sigma) (6U/1x10^7) and incubated on rotator for 30min at 4°C. Chromatin was digested with 3x 10sec sonication at 30% intensity with a Vibra-Cell probe. The supernatant was collected by centrifugation at 1300rpm for 30min at 4°C, and then diluted to 200mM KCl concentration. 30μl of Dynabeads (Invitrogen) were used per co-IP. Beads were washed 2x in 200mM KCl IP Buffer, re-suspended in IP Buffer with 10μg of the IP antibody, or an IgG-containing serum to match the species of the IP antibody and placed on rotator for 5h at 4°C. Beads were washed 3x in IP buffer and then incubated in 1mg chromatin lysate on a rotator overnight at 4°C. The beads were washed, re-suspended in 2X Laemmli Buffer (Bio-Rad) and boiled for 10min.

UMI-4C library preparation - 1x10^7 cells were fixed at RT for 10min in 1% formaldehyde and fixation was quenched with 0.125M Glycine for 5min. Cells were then lysed on ice in 10ml Lysis Buffer (10mM NaCl, 10mM Tris-HCl pH 8.0, 0.25% NP40, protease inhibitor) for 30min, followed by 10 strokes of douncing using a tight pestle. Nuclei were pelleted, 8min 700 rcf, washed in 1ml 1.2X DpnII buffer in Protein LoBind tubes (Eppendorf) and resuspended in 500 μl 1.2X DpnII buffer. 15ul of 10% SDS was added and incubated for 1hr at 37°C shaking at 650 rcf. 50ul of 20% TritonX was added to quench the SDS and incubated for 15 min at 37°C with shaking. 750U of DpnII was added and incubated overnight at 37°**C** with interval shaking. The next morning, nuclei were pelleted at 4°C by 650 rcf for 5 min and resuspended in 500μl 1X DpnII buffer. 500U DpnII was added and incubated for an additional four hours. The nuclei were washed twice in 100 μl of 1X T4 Ligase Buffer and resuspended in 200 μl Ligase Buffer**.** 6ul of T4 DNA Ligase was added and incubated for 3hr at 16°C**.** Nuclei were then pelleted, resuspended in 200 μl 1x fresh Ligase Buffer, 6μl of T4 DNA Ligase added, and incubated overnight at 16°C. Samples were treated with 20μl of ProtK (NEB Molecular Biology Grade), incubated for 3 hrs at 55°C and 5 hrs at 65°C to reverse crosslinks. Samples were treated with RNase A (PureLink, Invitrogen) for 1 hr at 37°C and DNA was extracted and precipitated

overnight. For library preparation, 3x5μg of ligated DNA was sonicated using Covaris (10% duty cycle, intensity 5, cycle burst 200, 70sec). Samples were end-repaired using DNA PolII Klenow Large Fragment (NEB), A-tailed using Klenow (exo-) (NEB), and Illumina indexed adapters ligated using Quick DNA Ligase (NEB). Reactions were denatured at 95°C for 3 min, placed on ice, and purified using 1.2X SizeSelect AmpPure beads to recover ssDNA. Libraries were amplified using GoTaq (Promega), with 20 cycles for PCR1 and 15 cycles for nested PCR2 on 50% material from 1st PCR. For custom UMI bait sequences, see Table S3.

Hi-C and UMI-4C-seq analysis

Hi-C libraries were analysed as previously described (Barrington et al., 2019). UMI-4C tracks were processed using the 'umi4cPackage' pipeline (v0.0.0.9000) (Schwartzman et al., 2016). Briefly, raw reads are parsed through the UMI-4C pipeline, those reads containing the bait and padding sequence are retained and de-multiplexed. Retained reads are split based on a match to the restriction enzyme sequence to create a segmented fastq file. The first 10 bases of read 2 are extracted and attached to the segments derived from each read pair. Mapping to mm10 is done with Bowtie2. Read pairs that have reverse complement segments are mapped to a restriction fragment ID, with the fragment ID, strand and distance from each end represented within a fragment-chain table. UMI filtering is used to determine the number of molecules supporting each ligation event. The resulting UMI-4C tracks are then imported into R, and data from multiple bait replicates can be merged by summing the molecule counts per ligated fragment, at which point contact intensity profiles and domainograms around the viewpoint can be generated. The contact intensity profile represents the mean number of ligations within a genomic window, with the resolution of the contact intensity profile being determined by the window size (set to 15 here). The domainogram reports the mean contact per fend at a series of window sizes, a stacked representation of contact intensity values in increasing window sizes from 10 to 300 fragment ends, their colour can be used to identify peak locations. ES and NSC contact profiles were compared after normalisation to correct for bias (see Schwartzman et al for further details). For the compared profiles, the total molecule count for restriction fragment ends for each are calculated at three ranges around the viewpoint. One profile is selected as reference and the second is scaled to the first using the ratio in total molecule counts between the two profiles. Below the contact profile is the profile resolution indicator, which shows the number of fends required to include at least 15 UMI molecules. The darker the colour, the larger the window size required. The domainogram at the bottom represents the log2 ratio between the domainogram values of the compared profiles and highlights locations where ESC has more contacts than NSC or vice versa.

REFERENCES

Barrington, C., et al. 2019. Enhancer accessibility and CTCF occupancy underlie asymmetric TAD architecture and cell type specific genome topology. Nature Communications 10, 2908. doi:10.1038/s41467-019-10725-9

Fidalgo, M., et al. 2016. Zfp281 Coordinates Opposing Functions of Tet1 and Tet2 in Pluripotent States. Cell Stem Cell 19, 355–369. doi:10.1016/j.stem.2016.05.025

Percharde, M., et al. 2018. A LINE1-Nucleolin Partnership Regulates Early Development and ESC Identity. Cell 174, 391–405.e19. doi:10.1016/j.cell.2018.05.043

Schwartzman, O., et al. 2016. UMI-4C for quantitative and targeted chromosomal contact profiling. Nat Meth 13, 685–691. doi:10.1038/nmeth.3922

Tapial, J., et al. 2017. An atlas of alternative splicing profiles and functional associations reveals new regulatory programs and genes that simultaneously express multiple major isoforms. Genome Res. 27, 1759–1768. doi:10.1101/gr.220962.117

Table S3. List of Primers used in this study, related to all Figures

PRIMER	Application	SEQUENCE
Actin B_fwd	qPCR	GGTGTTGAAGGTCTCAAACATG
Actin B_rev	qPCR	GAACATGGCATTGTTACCAACTG
Stag1_A_fwd	qPCR	GTACGGTCAGAATAGAGATGTTTCG
Stag1_A_rev	qPCR	GACACTGTCGAATCAGGACTCC
Stag1_C_fwd	qPCR	CCCTAATCTGGCTTTTCTAGAAGTAC
Stag1_C_rev	qPCR	CAGACATCCTGTCATCTTCACC
Stag2_fwd	qPCR	CCGAAATTCTTTGCTAGCTGG
Stag2_rev	qPCR	CCACATACTGTCACTGCTACTGC
Stag2_B_fwd	qPCR	CCGAAATTCTTTGCTAGCTGG
Stag2_B_rev	qPCR	CCACATACTGTCACTGCTACTGC
Smc3_fwd	qPCR	CAAGGATTTGGAGGATACCGAG
Smc3_rev	qPCR	CAACTCGAGCTTTGACTTCATTG
Smc1A_fwd	qPCR	CATGAGATGGAAGAGATTCGC
Smc1A_rev	qPCR	CCTTTGACAGTGGCAGTTTG
Nanog_fwd	qPCR	CGGACTGTGTTCTCTCAGGC
Nanog_rev	qPCR	CACCGCTTGCACTTCATC
Oct4_fwd	qPCR	CCCAAGGTGATCCTCTTCTGCTT
Oct4_rev	qPCR	GAGAAGGTGGAACCAACTCCCG
Dnmt3a_fwd	qPCR	GCTTCTCCGACTGTGGCC
Dnmt3a_rev	qPCR	CACCAAGACACAATTCGGC
Stag1_SATS_fwd	qPCR	GACACCTCTGTGACTAGTGAAGCC
Stag1_SATS_rev	qPCR	TGCTGGAGAAGCTATTCCACAG
L1spa-ORF1_fwd	qPCR	GAGAACACTGCTAAAGAGTTACAAGTCC
L1spa-ORF1_rev	qPCR	GGTCTAGTATGGTTTTGTTCATTTCC
MERVL-B_fwd	qPCR	TGGTGGTCGAGATGGAGGTTA
MERVL-B_rev	qPCR	CCGTGAATGGTGGTTTTAGCA
IAP_fwd	qPCR	GCACCCTCAAAGCCTATCTTAT
IAP_rev	qPCR	TCCCTTGGTCAGTCTGGATTT
Gm6763_fwd	qPCR	GCACCATACTGCAGACCAAAA
Gm6763_rev	qPCR	AGTGCACAGCAGATTTCTTCAAC
Dux_fwd	qPCR	AACTCCTCCTCCTTGATCAACTG
Dux_rev	qPCR	CTTCTCTCTGTGGCCAAAAGC
AW822073_fwd	qPCR	GTAGAAATTCTGGCAGCTGGG
AW822073_rev	qPCR	TTGATAGAGCAAGAGCTCCAGG

Gm4981_fwd	qPCR	GATAATAATGAAGTGCCTTCTGCAG
Gm4981_rev	qPCR	GTGAAGCCTAGTCCTAGTGTCCC
Stag1exon2-rev	5' RACE	TTGCTGGAGAAGCTATTCCACAGTACA
Stag1exon8-rev	5' RACE	CTCTTTACGTTTCTGAAGTAGTAACTCCAGTC
Stag1exon23-fwd	3' RACE	GCTGTTTGCCAACAGTGCCTATCTAATG
Stag1_SATS_5p-end	PCRminiscreen	ACTTCCGGTTCTAACTCCTTCCTC
Stag1_can-5p-end	PCRminiscreen	ATTGGCGTGTGGAAAATGC
Stag1_altex1-5p-end	PCRminiscreen	TGAAGGATGACAGCTACGCAC
Stag1_lastexon_rev	PCRminiscreen	TTCAGAACATAGGCATTCCAAATC
Stag1_intrn25_end_rev	PCRminiscreen	GTGCAGGGTGAGAGACATGG
Nanog_UMI4Cbait_nested	UMI-4C	AATGATACGGCGACCACCGAGATCTACACTCTTTCCCTACACGACGCTCTTCCGATCTCTCAGGTGCCAAAACCAACAG
Nanog_UMI4Cbait_PCR1	UMI-4C	GAAGCAGATGAGCACCAGACAC
CTCF_UMI4Cbait_nested	UMI-4C	AATGATACGGCGACCACCGAGATCTACACTCTTTCCCTACACGACGCTCTTCCGATCTACCTGGAGGCCAGCATAGAC
CTCF_UMI4Cbait_PCR1	UMI-4C	GGTGCTAACCTGGGCTTTG
Stag1canATG	Gateway cloning	GGGGAAGTTTGTACAAAAAAGCAGGC*TTCACC***ATGATTACTTCAGAGTTACCAGTGTTACAG**
Stag1canend	Gateway cloning	GGGGACCACTTTGTACAAGAAAGCTGGG**T***CGAACATAGGCATTCCAAATC*
intron25_end	Gateway cloning	GGGGACCACTTTGTACAAGAAAGCTGGGTCATTATCTATTTCTCTACCATACAGAAAGG
Stag1-exon5_exon6ATG_Kozak	Gateway cloning	GGGGAAGTTTGTACAAAAAAGCAGGCTTC*ACCATG*ATGTTTCGAAATATGCAGAACG
mSTAG1_e.33_FWD_2	CRISPR/Cas9 genotyping	ACTTCTTTGACTCTGCAGCTATCAT
mSTAG1_3' UTR_REV_2	CRISPR/Cas9 genotyping	AAACACACACACATCTGTACTGAGA
V5_FWD	CRISPR/Cas9 genotyping	CCTAACCCTCTCCTCGGTCT
Stag1_3'UTR_REV	CRISPR/Cas9 genotyping	CGGCGATTAGAACGAGCTGC
Stag1_3' gRNA_2	CRISPR/Cas9 gRNA	TTCTTCAGACTTCAGAACAT
